# Exclusive Se‐O Coordination and Fe‐doping Complementation: A Catalytic Strategy for Enhanced Sulfur Redox in Li‐S Batteries

**DOI:** 10.1002/advs.202513049

**Published:** 2025-11-29

**Authors:** Zhao Yang, Yu Wang, Jingchen Han, Tong Wu, Yongqing Fu, Qingsheng Wu, Ming Wen

**Affiliations:** ^1^ School of Chemical Science and Engineering Shanghai Key Laboratory of Chemical Assessment and Sustainability Tongji University Shanghai 200092 China; ^2^ Faculty of Engineering and Environment Northumbria University Newcastle upon Tyne NE99 UK

**Keywords:** bidirectional catalysis, Fe/Se co‐doping, Li─S batteries, polysulfides conversion, spinel Co_3_O_4_

## Abstract

Developing efficient electrocatalysts to accelerate redox kinetics and suppress lithium polysulfides (LiPSs) shuttling remains a key challenge for lithium‐sulfur batteries (LSBs). Although transition‐metal‐oxides exhibit strong adsorption for the LiPSs, their application is impeded by sluggish Li_2_S conversion. Herein, a catalytic strategy is proposed for enhanced sulfur redox in LSBs by complementing exclusive Se‐O coordination and Fe‐doping in spinel Co_3_O_4_ (Fe_0.1_Co_2.9_O_4_‐Se) electrocatalyst. This engineered intersecting‐porous nanoarchitecture, fabricated via an etching‐carbonization method, facilitates electron/mass transport and exposes abundant electroactive sites. Fe^3+^ substitution at octahedral Co^3+^ sites synergizes with exclusive Se‐O coordination, narrows Co_3_O_4_’s bandgap, and elevates the d‐band center, thereby enhancing conductivity and strengthening the LiPSs’ adsorption. Such a design promotes instantaneous nucleation of Li_2_S and reduces the bidirectional catalytic energy barrier for achieving superior catalytic activity, outperforming Se‐Fe_0.1_Co_2.9_O_4,_ where Se in oxygen‐vacancies‐sites coordinates with metal/oxygen ions. Consequently, the S/Fe_0.1_Co_2.9_O_4_‐Se cathode delivers exceptional cycling stability with an ultralow capacity decay rate of 0.1054% per cycle over 500 cycles at 0.5 C. In a pouch cell with a high sulfur loading (6.1 mg cm^−2^) and lean electrolyte (E/S = 10 µL mg^−1^), it retains a capacity of 4.8 mAh cm^−2^ after 40 cycles. This work provides a new catalytic strategy for the design of high‐performance LSBs electrocatalysts.

## Graphical abstract

Exclusive Se‐O coordination and Fe‐doping complementation significantly improve conductivity and modulate the d‐band centers, which strongly anchor polysulfides in bidirectional catalysis and reduce the energy barriers for the Li_2_S nucleation and dissociation, achieving high‐apacity retention, exceptional rate capability, and stable cycling capability.

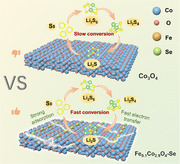

## 1 Introduction

Lithium‐sulfur batteries (LSBs) are gaining significant attention as the next‐generation transformative energy storage devices, owing to their high theoretical energy density (2600 Wh kg^−1^) and cost‐effective sulfur‐based electrochemistry.^[^ ^1^ ^]^ Nevertheless, their practical applications face critical obstacles due to sulfur insulativity, lithium polysulfides’ (LiPSs) shuttle effects, and sluggish sulfur redox kinetics.^[^ ^2^ ^]^ The shuttle effect, characterized by the LiPSs’ diffusion between electrodes, leads to severe loss of active material, lithium anode corrosion, and rapid capacity decay. Furthermore, the thermodynamically unfavorable nucleation of Li_2_S induces high overpotentials, which severely limit energy efficiency and rate capability.^[^ ^3^, ^4^ ^]^ To address these challenges, extensive effort has been made to develop various strategies, such as confining sulfur in conductive and porous carbon matrices, using a functional separator with tailored porous structures to physically or chemically trap the LiPSs, and designing efficient electrocatalysts to accelerate sulfur reduction reaction (SRR).^[^ ^5^, ^6^ ^]^ Among them, developing an efficient electrocatalyst is one of the key approaches to mitigate polysulfides’ shuttle effect, expedite redox kinetics, and enhance the LSBs’ cyclability.^[^ ^7^, ^8^ ^]^

Transition metal oxides (TMOs) can offer effective polar sites for capturing the LiPSs, via strong chemical interactions between metal cations or O^2−^ and LiPSs based on the Lewis acid‐base theory.^[^ ^9^, ^10^ ^]^ As one of the typical TMOs, spinel Co_3_O_4_ is a highly promising sulfur cathode catalyst due to its strong affinity to LiPSs, multivalent redox catalytic activity (Co^2+^/ Co^3+^), excellent charge‐discharge reversibility, good capability for nano‐engineering, and abundant natural resources.^[^ ^11^, ^12^, ^13^, ^14^ ^]^ More importantly, among 3d transition metal oxides (Mn, Fe, Co, Cu), Co_3_O_4_ shows its superior binding strength to the LiPSs (Co>Fe>Mn) and shuttle suppression capability.^[^ ^15^ ^]^ However, its practical application in sulfur conversion is limited by intrinsic poor conductivity and inadequate Li_2_S nucleation‐dissociation capabilities, especially under high‐sulfur‐loading or lean‐electrolyte conditions.^[^ ^16^, ^17^ ^]^ Therefore, it is becoming crucial to develop effective strategies to enhance Co_3_O_4_’s conductivity and catalytic activity.

Recently, adjustment of the coordination environment of active centers by doping engineering has demonstrated its great influence in improving the conductivity and catalytic activity of Co_3_O_4_ for high‐performance LSBs.^[^ ^18^ ^]^ As a promising strategy to tailor electronic configurations, optimize d‐band centers, and engineer defects, metal/non‐metal doping can improve charge carrier mobility, optimize catalytic thermodynamics/kinetics, and create extra active sites, ultimately boosting the LiPSs’ conversion efficiency.^[^ ^19^, ^20^ ^]^ For instance, metal cation doping, such as substituting Co^3+^ in Co_3_O_4_ with W^6+^/V^3+^, and non‐metal anion doping, such as incorporating Se into FeS_2_ or MoS_2_, have been demonstrated to reconfigure the electron structures, enhance conductivity, and accelerate the conversion process of LiPSs.^[^ ^12^, ^18^, ^21^, ^22^, ^23^, ^24^ ^]^ Despite these advances, the effectiveness of accelerating redox kinetics of the LiPSs and suppressing the shuttle effect through single‐doped TMOs is rather limited. Most current studies have been focused on the sulfur reduction reaction (SRR) process.^[^ ^12^, ^25^ ^]^ Moreover, for anion doping, a high‐temperature thermal treatment is commonly applied, but this process is costly and prone to forming non‐metal multi‐coordinated structures with low activity.^[^ ^26^, ^27^ ^]^ Notably, for most of these studies, the doping sites of cations or anions and their bonding environments are often not clearly elucidated. Simultaneous improvement of bidirectional sulfur redox processes by encompassing both SRR and sulfur evolution reaction (SER) through co‐doping metal cations and non‐metal anions in the TMOs has seldom been investigated. It is of vital importance to understand the structural changes triggered by metal/non‐metal co‐doping and the underlying mechanisms of adsorption and catalytic activities for both the SRR and SER.

To tackle these issues, in this work, a complementary catalytic strategy by integrating exclusive Se‐O coordination and Fe‐doping in spinel Co_3_O_4_ (denoted as Fe_0.1_Co_2.9_O_4_‐Se) is proposed to enhance bidirectional LiPSs redox catalysis in the LSBs. Fe_0.1_Co_2.9_O_4_‐Se nano‐dodecahedrons are precisely synthesized via an etching‐carbonization process, achieving numerous interconnected channels and abundant oxygen vacancies (O_v_). Such structures enable rapid ion/mass transport, enhanced sulfur accommodation capacity, and creation of abundant exposed active sites. Theoretical calculations reveal that the Fe_0.1_Co_2.9_O_4_‐Se induces effective electron transfer from the dopant sites to the adjacent oxygen atoms, generating electron‐enriched O atoms that effectively modulate the catalytic behavior. Furthermore, Fe doping narrows the bandgap and enhances conductivity, while the exclusive Se‐O coordination elevates the d‐band center and improves the adsorption energy and catalytic activity for the LiPSs. The complementation of these two factors lowers the energy barrier for the conversion of LiPSs in the Fe_0.1_Co_2.9_O_4_‐Se. Notably, such complementation promotes the instantaneous deposition of Li_2_S and enables a bidirectional catalytic process for Li_2_S redox. Fe‐doping preferentially enhances the capacity of Li_2_S deposition/dissolution, while Se coordination with O primarily reduces the initial overpotential of Li_2_S deposition/dissolution, thus achieving effective regulation of LiPSs. As a sulfur host, the S/Fe_0.1_Co_2.9_O_4_‐Se cathode demonstrates its exceptional polysulfide confinement and conversion efficiency, endowing the Li‐S battery with both enhanced rate capability and cycling stability (e.g., 0.1054% capacity decay per cycle over 500 cycles at 0.5 C). Practical feasibility is further validated via pouch cells, which achieve a high areal capacity of 4.8 mAh cm^−2^ over 40 cycles, highlighting its potential for energy storage applications.

## 2 Results and Discussion

### 2.1 Material Synthesis and Characterization

**Figure 1:**
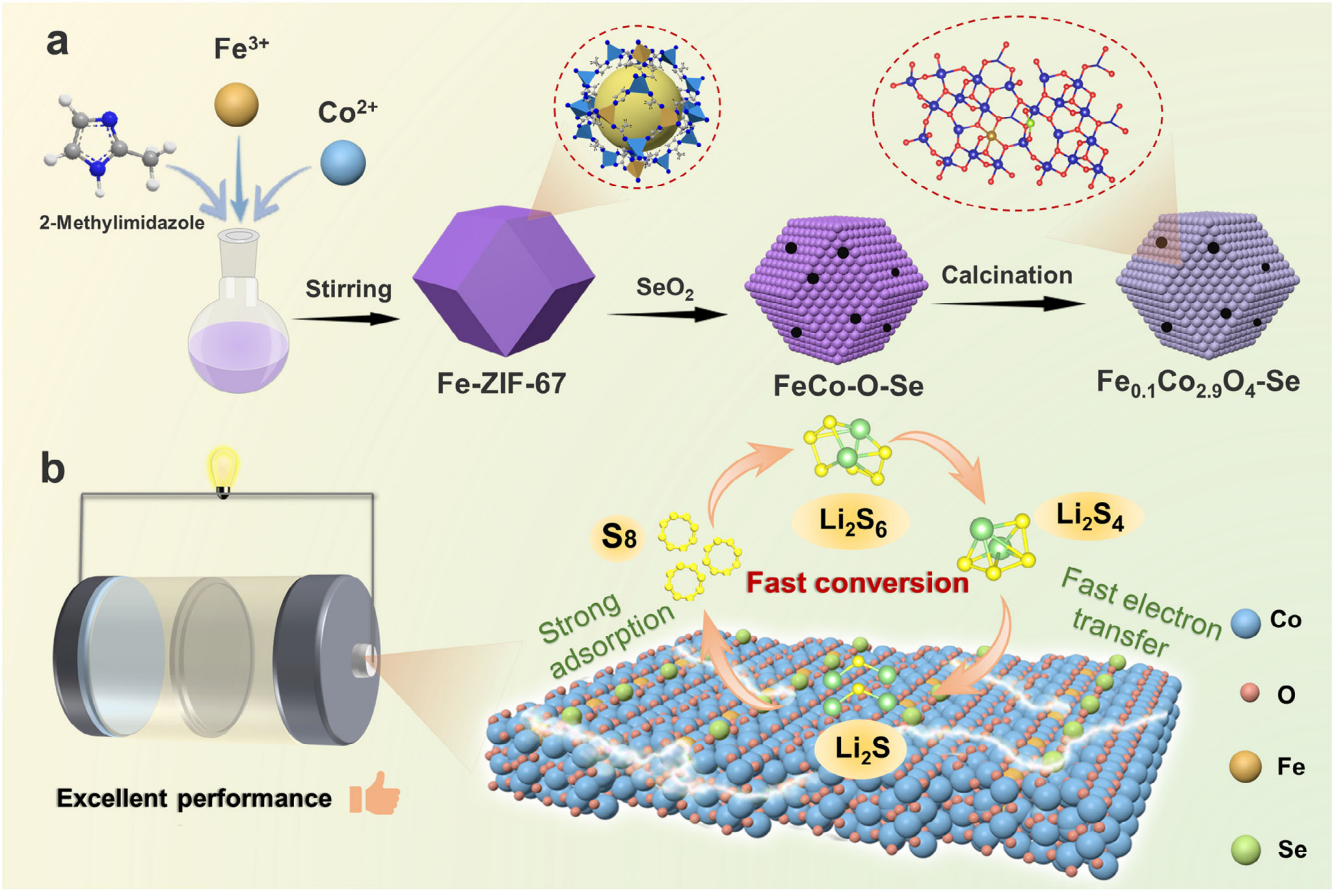
Schematic illustration of the synthesis route of Fe_0.1_Co_2.9_O_4_‐Se and the mechanism of its effect for LiPSs.

The fabrication procedure of the Fe_0.1_Co_2.9_O_4_‐Se catalyst is illustrated in **Figure** **1**a. Using Fe‐doped Co‐based metal‐organic framework (Fe/ZIF‐67) as the precursor, a synergistic method of selenic acid etching and controlled carbonization was developed to construct porous‐carbon supported Fe‐doped Co_3_O_4_ with Se‐O coordination. Initially, Fe‐Co dual‐coordinated metal‐organic framework (Fe/ZIF‐67) was synthesized by the coordinative self‐assembly of Fe^3^⁺ and Co^2+^ with 2‐methylimidazole ligands. Subsequently, H_2_SeO_3_, which was generated from the solvolysis of SeO_2_, gradually etched the Fe/ZIF‐67, and the released Co^2+^/Fe^3+^ ions reacted with SeO_3_^2−^ to form FeCo‐SeO_3_^2−^ (referred as FeCo‐O‐Se). The sequential reaction steps are listed as follows Equations (1)–(3):

(1) $$\mathrm{Se}O_{2}+H_{2}O\to H_{2}\mathrm{Se}O_{3}$$

(2) $$H_{2}\mathrm{Se}O_{3}\to 2H^{+}+{\mathrm{SeO}}_{3}^{2-}$$

(3) $$\mathrm{Fe}/\mathrm{ZIF}-67+{\mathrm{SeO}}_{3}^{2-}\to \mathrm{FeCo}-O-\mathrm{Se}+\mathrm{anionic}\;\mathrm{ligands}$$

The final stage involved thermal annealing of FeCo‐O‐Se under an Ar atmosphere at 550 °C. With the high‐volume ratio of water/ethanol, the matrix exhibited a relatively weak reduction capability toward SeO_3_^2−^, resulting in the formation of porous‐carbon supported Fe‐doped Co_3_O_4_ with Se‐O coordination (i.e., Fe_0.1_Co_2.9_O_4_‐Se). Crucially, our developed solvent engineering strategy enabled the precise regulation of SeO_2_’s solubility and evolution of Se valence state through adjusting the ethanol/water ratio, thereby controlling the Se coordination environment during the etching process. Remarkably, this methodology permits phase‐selective crystallization, yielding either (FeCo)_0.85_Se or heterostructured (FeCo)_0.85_Se/CoO composites (Figure S1, Supporting Information). The innovative selenic acid assisted etching strategy produces porous‐structured catalysts of Fe‐doped Co_3_O_4_ with Se‐O coordination environment and O_v_ in one step. This significantly increases the number of active sites and shortens the mass/electron transport paths, thereby enhancing the material's conductivity and catalytic activity, and demonstrating excellent catalytic performance in the catalytic conversion of sulfur cathodes in the LSBs (Figure 1b).

**Figure 2:**
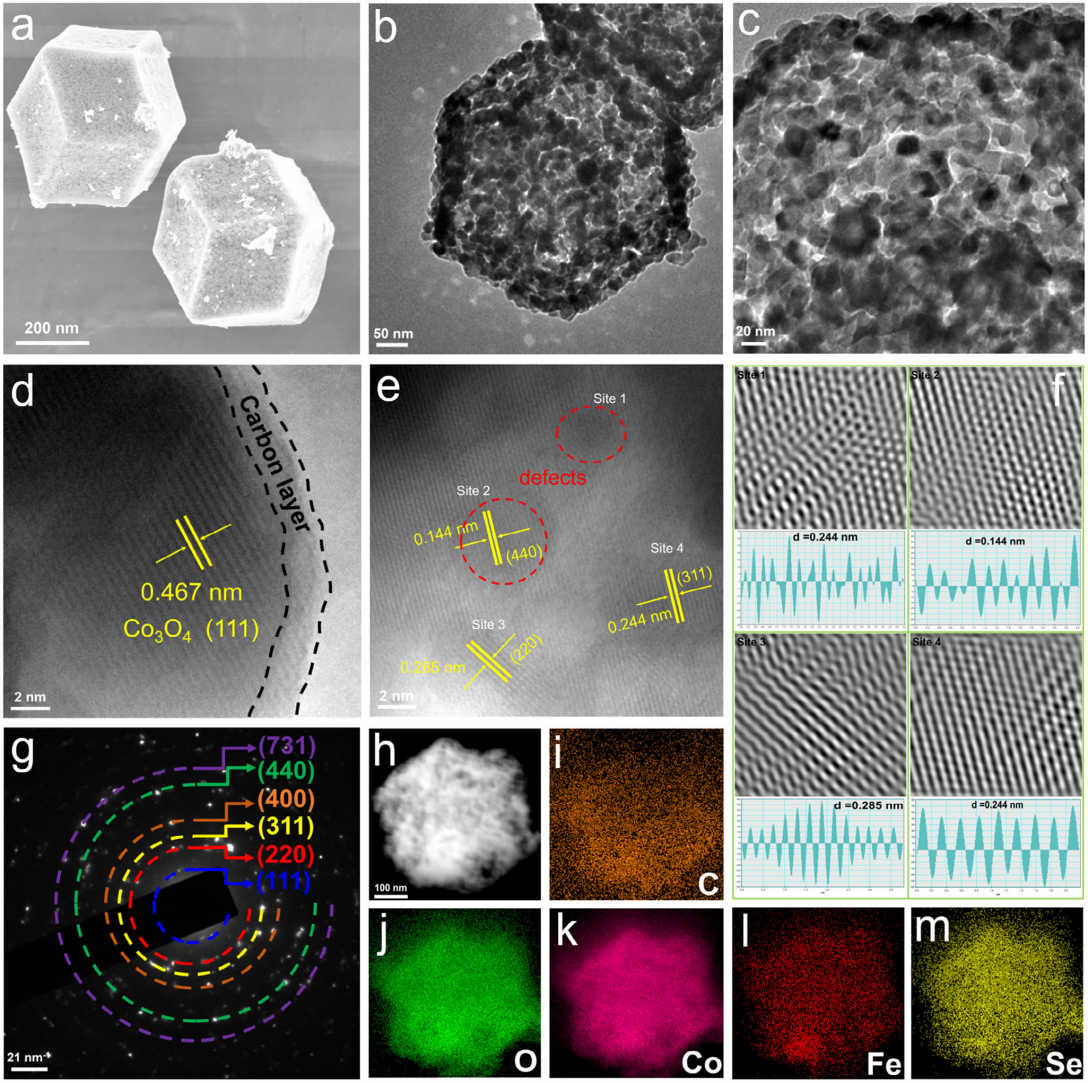
Morphology characterization of Fe_0.1_Co_2.9_O_4_‐Se. a) SEM image. b,c) TEM images. d,e) HRTEM images. f) The corresponding FFT images. g) SAED pattern. h–m) The elemental mapping images.

**Figure** **2** shows the morphology and microstructures of the prepared samples. Scanning electron microscope (SEM) images revealed that both ZIF‐67 and Fe/ZIF‐67 show well‐defined rhombic dodecahedral morphologies with their smooth surfaces, while iron doping induces a notable increase of average particle sizes from 300 to 500 nm (Figures S2 and S3, Supporting Information). X‐ray diffraction (XRD) analysis demonstrates the highly crystallized ZIF‐67 structures, which were remained unaltered upon Fe doping (Figure S4, Supporting Information). Post‐etching (1 h) of ZIF‐67 and Fe/ZIF‐67 with the selenic acid yielded Co‐O‐Se and FeCo‐O‐Se, where divergent architectural evolutions can be observed. The Co‐Se‐O has been transformed into a hollow structure, whereas the FeCo‐Se‐O has been developed into a partially etched porous structure (Figures S5 and S6, Supporting Information). Structure stability assessments showed a complete structural collapse in the Co_3_O_4_‐Se after the calcination process at 550 °C, contrasting with the preserved dodecahedral structure of Fe_0.1_Co_2.9_O_4_‐Se, which was inherited from its Fe‐ZIF‐67 precursor (Figure 2a and Figures S7–S10, Supporting Information). Additionally, Figure S11 (Supporting Information) illustrates the effects of non‐optimal Fe and SeO_2_ feeding ratios on the morphology and structures of the Fe_0.1_Co_2.9_O_4_‐Se. Their crystallinity is adversely affected by the increased amount of either Fe or SeO_2_. Moreover, an excessive amount of SeO_2_ induces a morphological transition from well‐defined dodecahedra to irregular nanosheets.

Transmission electron microscopy (TEM) images further unveiled the nanostructured features of the Fe_0.1_Co_2.9_O_4_‐Se, and those wrinkles textures and hierarchical pores were from the etching‐induced reconstruction process (Figure 2b). These pores not only significantly increase the number of exposed active sites but also enhance the material's specific surface areas, providing a larger reaction space for the storage and conversion of LiPSs. N_2_ adsorption‐desorption testing results (Figure S12, Supporting Information) reveal that the Fe_0.1_Co_2.9_O_4_‐Se has a superior specific surface area and the optimized pore size distribution, compared to those of Co_3_O_4_‐Se, Fe_0.1_Co_2.9_O_4_, and Co_3_O_4_. In particular, the Fe_0.1_Co_2.9_O_4_‐Se with micropores of ≈0.9 nm not only efficiently suppresses the dissolution of LiPSs through size exclusion (Li_2_S_4_ ≈1.1 nm), but also facilitates the efficient transport of Li^+^ ions (ionic diameter ≈0.64 nm).^[^ ^28^ ^]^ The microporous carbon matrix, which is engineered via the carbonization of organic ligand during the annealing process, effectively stabilizes and disperses active sites and enhances the conductivity. As shown in Figure 2c and Figure S13 (Supporting Information), the Fe_0.1_Co_2.9_O_4_‐Se nanoparticles with a size distribution of 17±7 nm are embedded within the porous carbon matrix, and further encapsulated by a 2 nm thickness carbon coating (Figure 2d). The formed carbon coating layer is beneficial to enhance the conductivity and structural stability and promote an effective charge transfer, thus can serve as a matrix to maintain the porous cavity structures during the electrochemical processes.^[^ ^29^ ^]^

High resolution TEM (HRTEM) image of Fe_0.1_Co_2.9_O_4_‐Se shows lattice spacings of 0.467, 0.144, 0.244, and 0.285 nm, which correspond to (111), (440), (311), and (220) planes of Co_3_O_4_, respectively (Figure 2e). Figure 2e shows the Fast Fourier transform (FFT) and inverse FFT patterns of regions 1‐4, which reveal that the lattice structures of the sites 3 and 4 are relatively intact, whereas those of the sites 1 and 2 exhibit a discontinuous pattern (Figure 2f). These defects are caused by Fe/Se co‐doping and the formation of O_v_ after annealing.^[^ ^30^ ^]^ The selected‐area electron diffraction (SAED) pattern shown in Figure 2g reveals the crystal planes of Co_3_O_4_. Furthermore, elemental mapping results obtained from the energy‐dispersive X‐ray spectroscopy (EDS) reveal the uniform distributions of Co, N, O, Fe, and Se in Fe_0.1_Co_2.9_O_4_‐Se (Figure 2h–m). The doping content of Fe in the catalyst is ≈1.166%, and the doping content of Se is ≈1.91%. The atomic ratio of Fe/Co is estimated to be 1:29, as listed in Table S1 (Supporting Information).

**Figure 3:**
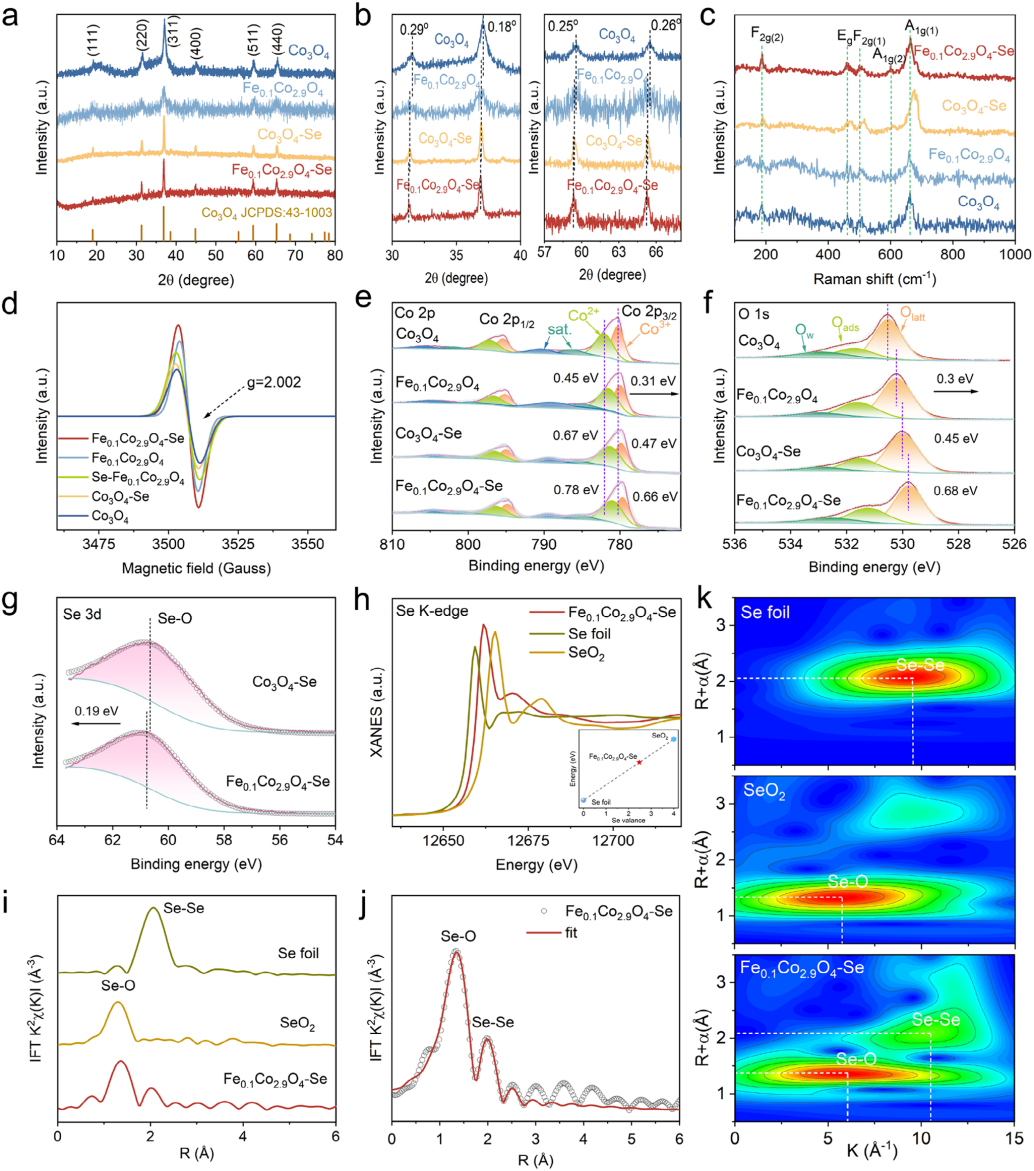
Structural characterizations of Fe_0.1_Co_2.9_O_4_‐Se, Co_3_O_4_‐Se, Fe_0.1_Co_2.9_O_4_, and Co_3_O_4_. a‐b) XRD patterns. c) Raman spectra. d) EPR spectra. High‐resolution XPS spectra of e) Co 2p, f) O1s, and g) Se 3d. h) Se K‐edge XANES spectra, and i) FT *K* ^2^‐weighted *χ*(*k*)‐function of EXAFS spectra. j) FT‐EXAFS fitting curve in R space of Fe_0.1_Co_2.9_O_4_‐Se at the Se K‐edges. k) Wavelet transforms for EXAFS spectra of Se foil, SeO_2,_ and Fe_0.1_Co_2.9_O_4_‐Se.

Crystalline structures of the prepared samples were characterized by XRD, and the obtained results are shown in **Figure** **3**a. After doping Fe and/or Se into Co_3_O_4_, all the observed diffraction peaks are similar to those of the spinel Co_3_O_4_ (JCPDS card, No. 43‐1003). There are no other diffraction peaks attributed to those of iron and selenium species, suggesting the low surface contents and effective dispersion of Fe and Se in the catalysts. Particularly, there are no characteristic peaks of cobalt selenides or selenates in the Fe_0.1_Co_2.9_O_4_‐Se, confirming the successful formation of the Fe/Se co‐doped Co_3_O_4_ rather than phase‐separated chalcogenide compounds.^[^ ^16^ ^]^ Detailed analysis in Figure 3b reveals that the diffraction peaks are shifted slightly to the lower‐angle side for the Fe_0.1_Co_2.9_O_4_‐Se, with their corresponding shifts of 0.29°, 0.18°, 0.25°, and 0.26° observed at 31.2° (220), 36.8° (311), 59.3° (511), and 65.2° (440), respectively. The peak shifts can be attributed to lattice expansion induced by the successful incorporation of Fe and Se doped into the spinel Co_3_O_4_,^[^ ^31^ ^]^ which is also consistent with the HRTEM results shown in Figure 2e. Furthermore, the peak intensities of Fe_0.1_Co_2.9_O_4_‐Se and Fe_0.1_Co_2.9_O_4_ are significantly lower compared to those of Co_3_O_4_. This reveals that Fe has replaced Co, and because of substitutional Fe‐doping or Se‐doping, structural defects such as lattice distortions and O_v_ have been generated.^[^ ^32^ ^]^

In Raman spectra (Figure 3c), a band at ≈190 cm^−1^ is attributed to the F_2g(2)_ vibrational mode of the CoO_4_ tetrahedron. Four bands at 470, 510, 610, and 680 cm^−1^ are assigned to the E_g_, F_2g(1)_, A_1g(1)_, and A_1g(2)_ vibrational modes of the CoO_6_ octahedron, respectively.^[^ ^33^, ^34^ ^]^ No additional peaks can be found for cobalt selenate or cobalt selenide, which is in good agreement with the XRD results. Compared to Co_3_O_4_, a slight redshift and intensity attenuation of the A_1g_ peak corresponding to CoO_6_ octahedron Co─O (683–678 cm^−1^) vibration in Fe_0.1_Co_2.9_O_4_ can be observed. This is due to the changes in the O─Co─O bond lengths and the creation of vacancies caused by Fe^3+^ substitution, both of which reduce the Co─O vibration frequency.^[^ ^35^ ^]^ Conversely, the A_1g_ peak of Co_3_O_4_‐Se exhibits a blue shift, suggesting that the Se‐induced ligand field has been strengthened through altering the oxygen coordination.^[^ ^36^ ^]^ Based on both the Raman and XRD analyses results, Fe and Se doped Co_3_O_4_ partially alter the Co─O─Co bond length and change the corresponding lattice parameters, achieving atom rearrangement of Co_3_O_4_ and significantly creating defect sites.^[^ ^16^, ^36^ ^]^

To further verify the reliability of defect introduction via Fe and Se doping, electron paramagnetic resonance (EPR) technology was employed to evaluate unpaired electrons on Co atoms in the materials.^[^ ^18^ ^]^ As the obtained results shown in Figure 3d, all the samples exhibit a characteristic signal at g = 2.002, corresponding to the unpaired electrons induced by the partial removal of O.^[^ ^36^ ^]^ The order of EPR peak intensities for all the samples is Fe_0.1_Co_2.9_O_4_‐Se > Fe_0.1_Co_2.9_O_4_ > Se‐Fe_0.1_Co_2.9_O_4_ > Co_3_O_4_‐Se > Co_3_O_4_, indicating that Fe doping results in superior O_v_ generation compared to that of Se doping. It is worth noting that Se‐doping does not reduce O_v_ in Fe_0.1_Co_2.9_O_4_‐Se and Co_3_O_4_‐Se samples, suggesting that Se does not fill the O_v_ sites. These results are completely different from that the Se‐doping significantly reduces O_v_ in Se‐Fe_0.1_Co_2.9_O_4_ when Se occupies the O_v_ sites.

Surface states and chemical environments of the Fe_0.1_Co_2.9_O_4_‐Se were further analyzed using X‐ray photoelectron spectroscopy (XPS).^[^ ^11^ ^]^ The survey spectrum (Figure S14, Supporting Information) confirms the coexistence of Fe, Co, Se, and O elements, consistent with the EDS results of elemental mapping and quantitative analysis (Table S2, Supporting Information). In the high‐resolution Co 2p spectrum (Figure 3e), the peaks at binding energies of 779.61, 780.94, 794.8, and 796.4 eV are attributed to Co^3+^2p_3/2_, Co^2+^2p_3/2_, Co^3+^2p_1/2_, and Co^2+^2p_1/2_, respectively, while the two peaks at binding energies of 785.6 and 789.4 eV are ascribed to the satellite peaks.^[^ ^37^ ^]^ Additionally, quantitative analysis of fitting results for the Co 2p spectra (Table S3, Supporting Information) shows that after Fe doping, the Co^2+^/Co^3+^ ratio is increased from 1.02 to 1.14, confirming that the introduced Fe^3+^ occupies the Co^3+^ octahedral (Co_oh_^3+^) sites of Co_3_O_4_. This observation can be further confirmed by the density function theory (DFT) calculation results shown in Figure S16 (Supporting Information). Such a site preference is energetically favorable as demonstrated in a previous study where the octahedral Fe^3+^ substitution exhibits a lower system energy than tetrahedral occupation.^[^ ^18^, ^34^ ^]^ Additionally, compared with that of Co_3_O_4_, the peaks of Co 2p in Fe_0.1_Co_2.9_O_4_, Co_3_O_4_‐Se, and Fe_0.1_Co_2.9_O_4_‐Se are negatively shifted by 0.31, 0.47, and 0.66 eV to the lower binding energy region.

The O 1s spectra show three components of lattice oxygen (O_latt_, 529.8 eV), surface‐adsorbed oxygen (O_ads_, 531.4 eV), and oxygen in adsorbed water (O_w_, 533.1 eV).^[^ ^38^ ^]^ As shown in Figure 3f, a shift of the O_latt_ peak toward the lower binding energy region can be observed after Se and Fe doping, indicating that electrons are migrated from the heteroatoms Fe and Se to Co_3_O_4_, which increases the electron densities of Co and O atoms. The O_ads_ peak at 531.4 eV originates from the O_2_^2−^ or O^−^ species, which were derived from the dissociated molecular oxygen adsorbed at O_v_.^[^ ^38^ ^]^ Compared with the ratios of O_ads_ in Co_3_O_4_ (20.81%), Fe_0.1_Co_2.9_O_4_ (27.57%), and Co_3_O_4_‐Se (21.39%), the corresponding value of the Fe_0.1_Co_2.9_O_4_‐Se is increased to 34.56% (Table S4, Supporting Information). This shows a significant increase in the O_v_ contents, which is correlated well with the EPR results. According to a previous study^[^ ^33^ ^]^ introduction of an appropriate concentration of O_v_ can improve the conductivity via regulating the electronic structure, thereby enhancing the electrocatalytic activity. The Fe 2p_3/2_ peaks are shifted positively by 0.25 eV (Fe^2+^) and 0.11 eV (Fe^3+^) as shown in Figure S17 (Supporting Information), demonstrating that the doping of Se leads to electron migration of Fe_0.1_Co_2.9_O_4_‐Se.^[^ ^21^, ^39^ ^]^ Concurrently, the Se 3d spectra analysis (Figure 3g) shows the exclusive Se─O bonding at 60.4 eV, with a 0.19 eV positive shift in Fe_0.1_Co_2.9_O_4_‐Se versus Co_3_O_4_‐Se, indicating that Se is mainly bound with O and more electrons are migrated due to the successful doping of the Fe element.^[^ ^29^, ^40^, ^41^, ^42^ ^]^ In the morphological and structural characterization of the Se‐Fe_0.1_Co_2.9_O_4_ sample (Figures S18 and S19, Supporting Information), however, for the Se 3d spectra exhibit a new peak at 54.3 eV, which corresponds to the Se‐metal bond,^[^ ^43^ ^]^ concomitant with the reduced O_v_ content. These results suggest that the Se has occupied the O_v_ sites and coordinates with metal/oxygen ions, which are quite different from the Se─O bonding configuration obtained in Fe_0.1_Co_2.9_O_4_‐Se.

X‐ray absorption spectroscopy (XAS) was employed to probe more detailed information about the electronic configurations and local coordination environment of Se species in the Fe_0.1_Co_2.9_O_4_‐Se. The obtained X‐ray absorption near‐edge structure (XANES) spectra of the Se K‐edge and reference standards are presented in Figure 3h. The absorption edge of Fe_0.1_Co_2.9_O_4_‐Se is located between the referential Se foil and SeO_2_ sample, suggesting that the Se species in the Fe_0.1_Co_2.9_O_4_‐Se possesses positive charges (0*<*n*<*4). Based on the linear relationship between the Se valence and pre‐edge position, the oxidation state of Se in Fe_0.1_Co_2.9_O_4_‐Se was determined to be + 2.5 (see the Inset of Figure 3h). The spectrum of Fourier‐transformed extended X‐ray absorption fine structure (FT‐EXAFS) for the Fe_0.1_Co_2.9_O_4_‐Se (Figure 3i) reveals two prominent peaks at 1.37 and 2.09 Å, which correspond to Se─O and Se─Se coordination shells, respectively.^[^ ^44^, ^45^ ^]^ Quantitative analysis through nonlinear least‐squares fitting (Figure 3j and Table S5, Supporting Information) showed that the Se─O and Se─Se bond distances are 1.63 ± 0.01 and 2.37 ± 0.01 Å, respectively, with an average oxygen coordination number of 1.4 ± 0.2 around the selenium centers. Complementary wavelet transform analysis of the EXAFS data (Figure 3k) identifies the dominant Se─O coordination in the Fe_0.1_Co_2.9_O_4_‐Se, without detectable Se‐metal interactions. This resolution‐enhanced technique confirms the structural consistency with the Fourier‐transform‐derived coordination environment.

Based on all the above results and discussions, a molecular structure model of the Fe_0.1_Co_2.9_O_4_‐Se was established, and the top view image is shown in Figure S20 (Supporting Information). Fe^3+^ dopants occupy the Co_oh_^3+^ sites in the Co_3_O_4_, whereas the selenium coordinates are exclusively with oxygen, excluding O_v_ via the Se─O bonds. This synergistic configuration optimizes the electronic structure of Fe_0.1_Co_2.9_O_4_‐Se, generating localized electron‐rich regions that enhance the LiPSs’ chemisorption and catalytic conversion. This coordination environment shows a sharp contrast to that of the Se‐Fe_0.1_Co_2.9_O_4_ composite, where the Se is located at O_v_ and coordinates with metal/oxygen ions.

### 2.2 DFT Calculations

**Figure 4:**
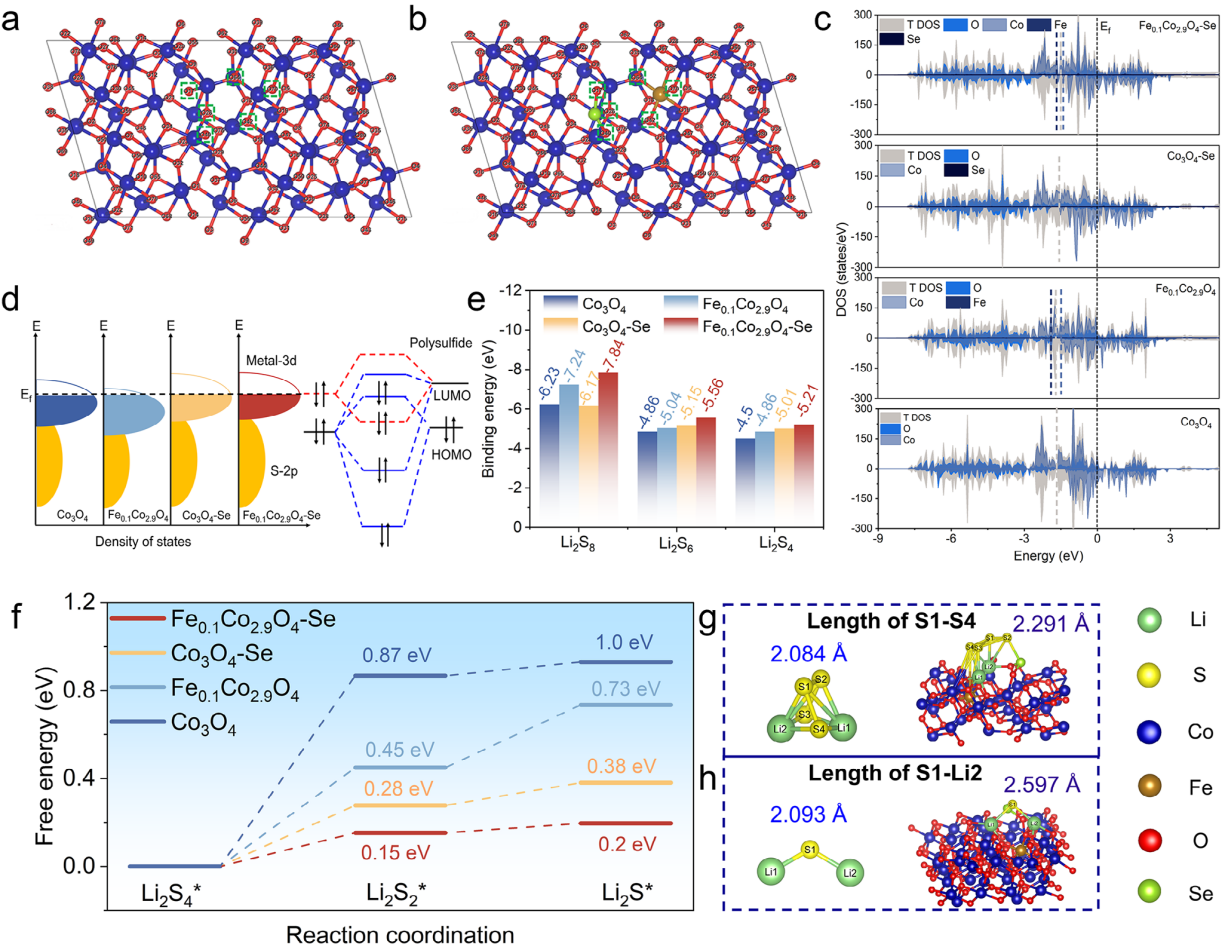
DFT calculation and analysis results. The Bader charge numbers of atoms of a) Co_3_O_4_ and b) Fe_0.1_Co_2.9_O_4_‐Se. c) Density of states. d) The orbital interactions between polysulfides and these catalysts. e) Calculated binding energies between LiPS molecules on the substrate. f) Gibbs free energy during the LPSs conversion reaction. g) The S‐S bond length of Li_2_S_4_ and h) the S‐Li bond length of Li_2_S on Fe_0.1_Co_2.9_O_4_‐Se substrate.

To understand the influences of Fe/Se co‐doping on the electronic structures of Fe_0.1_Co_2.9_O_4_‐Se and its effect for the adsorption‐catalysis of LiPSs, first‐principles calculations were performed to study the detailed redox process of LiPSs, including charge distribution, adsorption energy, and Gibbs free energy for the prepared samples. The most stable configurations with the lowest formation energies for the Fe_0.1_Co_2.9_O_4_‐Se, Co_3_O_4_‐Se, Fe_0.1_Co_2.9_O_4_, and Co_3_O_4_ were obtained, and the results are shown in Figure S21 (Supporting Information). The charge redistribution of O around the dopant was analyzed in detail by analyzing the Bader charges, which are shown in **Figure** **4**a,b. The obtained results reveal significant electron redistribution patterns induced by Fe/Se co‐doping. Theoretically, all the atoms in the Co_3_O_4_ model maintain a charge equilibrium state.^[^ ^19^ ^]^ The O atoms adjacent to the Fe modification are identified as O_42_, O_64_, and O_70_, respectively, and the charges on these three O atoms in the Co_3_O_4_ are 6.75 (O_42_), 6.88 (O_64_), and 6.64 (O_70_). Whereas the charges on these corresponding three O atoms in Fe_0.1_Co_2.9_O_4_‐Se are 6.79 (O_42_), 6.74 (O_64_), and 6.67 (O_70_), respectively. The O atoms adjacent to the Se modification are O_17_, O_26,_ and O_40_, and the charges on the three O atoms of 6.52 (O_17_), 6.86 (O_26_), and 6.4 (O_40_) in Co_3_O_4_ are changed to 6.89 (O_17_), 6.91 (O_26_), and 6.82 (O_40_) in Fe_0.1_Co_2.9_O_4_‐Se, respectively. Notably, these results show that Se has a stronger electron‐donating capability to O than Fe, so that the charge density of O atoms around the Se has been increased significantly. This charge redistribution indicates that the Fe/Se co‐doping induces localized electron accumulation at the adjacent O sites, which enhances the charge transfer capability and consequently improves the redox kinetics for the LiPSs.

To further elucidate the intrinsic mechanisms by enhancing redox activities of Co_3_O_4_ via Fe/Se co‐doping, the electronic structure evolution was systematically investigated through analysis of density of states (DOS) and the corresponding d‐band center values (Figure 4c and Figure S22, Supporting Information). Compared to Co_3_O_4_ and Co_3_O_4_‐Se, Fe_0.1_Co_2.9_O_4_ and Fe_0.1_Co_2.9_O_4_‐Se exhibit more pronounced hybridization bands and higher total DOS (TDOS) values at the Fermi level (E_f_ = 0). Furthermore, compared to Co_3_O_4_, the Fe_0.1_Co_2.9_O_4_ exhibits a smaller state gap at the Fermi level due to the formation of a new impurity band (Figure S23, Supporting Information), indicating that the incorporation of Fe enhances the electrical conductivity of the material, which can potentially accelerate the charge transfer capability with the LiPSs.^[^ ^21^, ^39^, ^46^ ^]^ Moreover, Fe/Se co‐doping leads to the upward shift of d‐band center of pristine Co_3_O_4_ from −1.63 to −1.57 eV, which can enhance the adsorption of LiPSs.^[^ ^47^ ^]^ What's more, because the upward shift of the d‐band center leads to a higher filling rate of the lowest unoccupied molecular orbital (LUMO) of the polysulfides, it becomes easier for the hybridization to occur between polysulfides and active sites.^[^ ^48^ ^]^ The interaction between LiPSs and Fe_0.1_Co_2.9_O_4_‐Se is also much stronger than that of other comparative samples (Figure 4d). To validate the above hypothesis, we systematically simulated the stable adsorption configurations of Li_2_S_x_ on different catalyst surfaces (Figures S24–S28, Supporting Information). As the results shown in Figure 4e, the Fe_0.1_Co_2.9_O_4_‐Se exhibits superior adsorption energies of 7.84, 5.56, and 5.21 eV for Li_2_S_8_, Li_2_S_6_, and Li_2_S_4_, respectively. These values are substantially higher than those of Co_3_O_4_‐Se (6.17, 5.15, and 5.01 eV), Fe_0.1_Co_2.9_O_4_ (7.24, 5.04, and 4.86 eV), and the pristine Co_3_O_4_ (6.23, 4.86, and 4.5 eV), confirming its exceptional chemical affinity for the LiPSs and effective suppression of LiPSs’ shuttle effect.

Further investigation into the catalytic activities of Fe_0.1_Co_2.9_O_4_‐Se was conducted by Gibbs free energy calculation. As the results shown in Figure 4f, the obtained Gibbs free energy data demonstrate that the process to reduce Li_2_S_4_ into Li_2_S requires an endothermic driving force. The Fe_0.1_Co_2.9_O_4_‐Se achieves remarkably lower energy barriers (0.15 and 0.05 eV) if compared to those of Co_3_O_4_‐Se (0.28 and 0.1 eV), Fe_0.1_Co_2.9_O_4_ (0.45 and 0.28 eV), and Co_3_O_4_ (0.87 and 0.13 eV). Figure 4g shows the molecular structure models of Li_2_S_4_ and Li_2_S_4,_ which are adsorbed on the Fe_0.1_Co_2.9_O_4_‐Se's surface. After adsorption, the bond length of S1‐S4 (Li_2_S_4_) is increased from 2.084 to 2.291 Å, which effectively weakens the S‐S bridge bonds, thereby promoting the reduction conversion of LiPSs. Figure 4h shows the molecular structure models of Li_2_S and Li_2_S, which are adsorbed on the Fe_0.1_Co_2.9_O_4_‐Se surface. The calculated results show that the S1‐Li2 (Li_2_S) bond is significantly elongated from 2.093 to 2.597 Å, indicating that the Fe_0.1_Co_2.9_O_4_‐Se can vigorously catalyze the dissolution of Li_2_S and promote the oxidation process of Li_2_S.^[^ ^37^ ^]^ Therefore, the designed Fe_0.1_Co_2.9_O_4_‐Se can promote the bidirectional conversion of sulfur, thereby accelerating reaction kinetics while improving overall conversion efficiency.

### 2.3 Nucleation and Dissociation Behavior of Li_2_S

Based on the above DFT calculations results, the structure‐activity relationship underlying the exceptional catalytic activity of Fe_0.1_Co_2.9_O_4_‐Se toward the LiPSs can be elucidated. To correlate the Fe/Se co‐doping strategy with the enhanced electrochemical behavior, we evaluated the LiPSs’ anchoring capabilities for all the prepared catalysts. Initial static adsorption tests revealed striking visual differences. When the equal catalyst masses were introduced into 5 mL of 2 mm Li_2_S_6_ solution, the Fe_0.1_Co_2.9_O_4_‐Se was completely decolorized from the original deep yellow solution within 3 h (Figure S29, Supporting Information), outperforming those of Co_3_O_4_‐Se and Fe_0.1_Co_2.9_O_4_ (light yellow) and notably much better than that of the Co_3_O_4_ (deep yellow). Quantitative ultraviolet‐visible analysis was performed for these studies, and the results show their absorbance intensities in the order of Fe_0.1_Co_2.9_O_4_‐Se < Co_3_O_4_‐Se < Fe_0.1_Co_2.9_O_4_ < Co_3_O_4_ < blank solution. The potential interaction between Fe_0.1_Co_2.9_O_4_‐Se and LiPSs was further revealed by the XPS analysis. As shown in Figure S30 (Supporting Information), after adsorbing the Li_2_S_6_, the Co 2p_3/2_ spectra exhibited a positive shift of 0.28 eV for the Co^3+^ peak and 0.60 eV for the Co^2+^ peak, indicating the transfer of electrons from Co to Li_2_S_6_. Concurrently, the O 1s peak was found to shift toward the higher binding energy side, revealing the electron donation from oxygen sites to Li_2_S_6_ and a strong interaction between Fe_0.1_Co_2.9_O_4_‐Se and LiPSs.^[^ ^37^, ^49^ ^]^ The enhanced adsorption stems from Fe/Se co‐doping induced electronic structure modulation, optimized local coordination environments, and abundant active sites for LiPSs immobilization offered by the porous structure.

**Figure 5:**
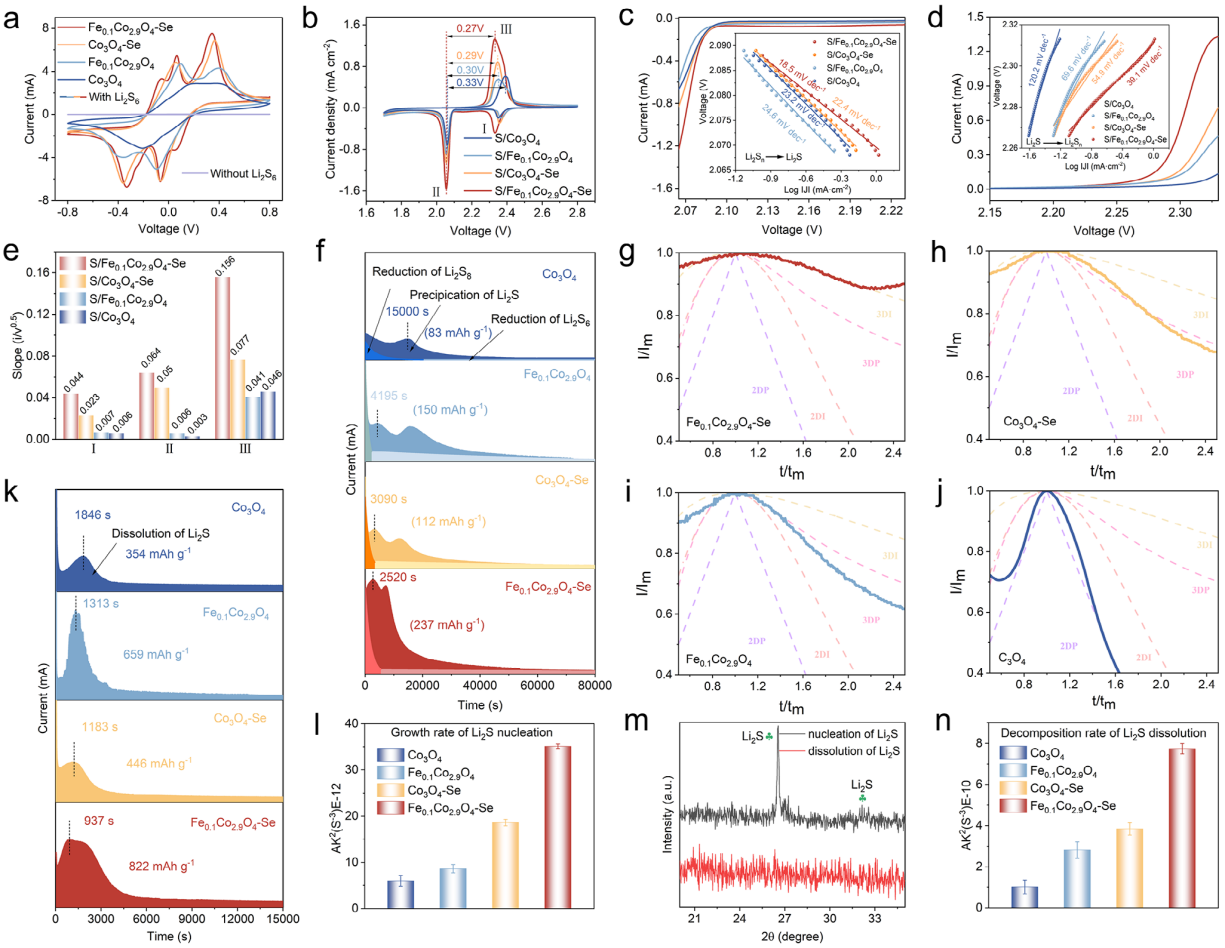
a) CV curves of different symmetrical cells. b) CV curves of different electrodes at 0.1 mVs^−1^. c, d) Corresponding Tafel plots of Peak II (c) and Peak III (d) in (b) for different electrodes. e) Comparison of the Tafel slope of the cathode with four catalysts. f) Potentiostatic discharge profiles for Li_2_S deposition. g‐j) Dimensionless current‐time transient for the Li_2_S nucleation process on various electrodes. k) Potentiostatic charge curves for Li_2_S decomposition. l) Corresponding calculated the growth rate of Li_2_S nucleation (Error bars represent standard deviation from three independent cells). m) XRD curve of Li_2_S nucleation and dissociation in S/Fe_0.1_Co_2.9_O_4_‐Se sample. n) Corresponding to the calculated decomposition rate of Li_2_S dissolution.

For high‐performance catalysts, both strong anchoring and superior catalytic activity for LiPSs are indispensable.^[^ ^24^ ^]^ To probe the electrocatalytic performance of the Fe_0.1_Co_2.9_O_4_‐Se and its conversion reaction kinetics for sulfur species, we constructed symmetrical cells with identical catalyst electrodes (in a sulfur‐free configuration) for evaluating the conversion rate of the Li_2_S_6_ electrolyte.^[^ ^50^ ^]^ **Figure** **5**a shows the obtained cyclic voltammetry analysis results, which reveal that all the electrodes of Co_3_O_4_‐Se, Fe_0.1_Co_2.9_O_4_, and Co_3_O_4_ exhibit merged redox peaks with attenuated current densities, indicating their sluggish LiPSs conversion kinetics. However, the Fe_0.1_Co_2.9_O_4_‐Se system demonstrates remarkably enhanced peak current densities and well‐defined redox couples, confirming that the Fe/Se co‐doping in Co_3_O_4_ remarkably accelerates the redox conversion kinetics of LiPSs.^[^ ^51^, ^52^, ^53^ ^]^ Notably, the Fe_0.1_Co_2.9_O_4_‐Se electrode maintains stable peak profiles with minimal potential shifts (ΔV < 28 mV) with increasing the scan rates (3–20 mV/s). These results show significant differences with those of the other three control samples (especially Co_3_O_4_, which shows significant peak broadening and current decay in Figure S31, Supporting Information). Analysis results from the electrochemical impedance spectroscopy (EIS) further corroborate the above analysis. The Fe_0.1_Co_2.9_O_4_‐Se symmetrical cell exhibits a smaller charge transfer impedance in the EIS spectrum if compared to those of the other control samples (Figure S32, Supporting Information). This result can be attributed to the Fe/Se co‐doping inducing electronic structure optimization, which facilitates simultaneous Li^+^ diffusion and charge transfer.^[^ ^54^, ^55^ ^]^

The S/Fe_0.1_Co_2.9_O_4_‐Se cathode was further prepared via the melt‐diffusion method (Experimental Section in Supporting Information). Post‐sulfur‐loading characterization confirmed that the structural integrity was preserved in the S/Fe_0.1_Co_2.9_O_4_‐Se, maintaining its original polyhedral morphology. XRD and thermogravimetric analysis (TGA) verify the high sulfur loading of over 70 wt.% (Figures S33–S36, Supporting Information).

To further explore the immobilization and reversible catalysis of LiPSs intermediates, especially the insoluble Li_2_S_2_ and Li_2_S, the electrochemical performance and reaction kinetics of cathodes were investigated in detail. Figure 5b shows the CV curves of coin cells assembled with different materials (including S/Fe_0.1_Co_2.9_O_4_‐Se, S/Co_3_O_4_‐Se, S/Fe_0.1_Co_2.9_O_4_, and S/Co_3_O_4_) as the cathode and a lithium foil as the anode, measured at a voltage window of 1.7–2.8 V and a scan rate of 0.1 mVs^−1^. All the curves exhibit two cathodic peaks and a single anodic peak. The cathodic peaks correspond to the reduction of solid S_8_ to soluble long‐chain LiPSs (Li_2_S_x_, 4≤x≤8) (peak I) and further reduction from soluble long‐chain LiPSs to short‐chain Li_2_S_2_/Li_2_S (peak II), while the anodic peak corresponds to the multi‐step oxidation of short‐chain LiPSs back to S_8_.^[^ ^56^, ^57^ ^]^ The S/Fe_0.1_Co_2.9_O_4_‐Se cathode exhibits much higher redox currents, and its oxidation peak is shifted toward lower potentials, if compared with those cathodes of S/Co_3_O_4_‐Se, S/Fe_0.1_Co_2.9_O_4_, and S/Co_3_O_4_, indicating the low electrochemical polarization and enhanced sulfur oxidation kinetics.^[^ ^37^ ^]^

Moreover, Tafel slope analysis was performed to determine the catalytic kinetics. As shown in Figure 5c, peak II represents the nucleation process of soluble LiPSs to Li_2_S. The S/Fe_0.1_Co_2.9_O_4_‐Se shows the lowest slope of 18.5 mV dec^−1^, whereas the values for other electrodes are 23.2 mV (S/Co_3_O_4_), 24.6 mV (S/Fe_0.1_Co_2.9_O_4_), and 22.4 mV (S/Co_3_O_4_‐Se). In Figure 5d, Peak III represents the dissociation of Li_2_S, and the Fe_0.1_Co_2.9_O_4_ also shows the lowest Tafel slopes of 39.1 mV dec^−1^ versus 120.2 mV (S/Co_3_O_4_), 69.9 mV (S/Fe_0.1_Co_2.9_O_4_), and 54.9 mV (S/Co_3_O_4_‐Se). These results indicate that the S/Fe_0.1_Co_2.9_O_4_‐Se possesses a superior bidirectional catalytic effect on the conversion of LiPSs, since a smaller Tafel slope implies a faster LiPSs conversion kinetic process. Furthermore, the conversion kinetics were evaluated based on the cyclic voltammetry curves obtained at different scan rates. Scan‐rate‐dependent CV studies (Figure S37, Supporting Information) validate that the S/Fe_0.1_Co_2.9_O_4_‐Se exhibits the highest redox current peak intensities and the lowest polarization compared with the other electrodes, demonstrating its excellent redox kinetics. These CV curves show a linear relationship between peak current (I_p_) and the square root of the scan rate (v^1/2^), representing the corresponding Li^+^ ion diffusion coefficient in the redox process.^[^ ^56^ ^]^ As can be seen from Figure 5e and Figure S38 (Supporting Information), the highest slope of S/Fe_0.1_Co_2.9_O_4_‐Se among all the samples in each redox process verifies its fastest Li^+^ ion diffusion rate and sulfur conversion kinetics,^[^ ^58^ ^]^ which can be confirmed by the EIS analysis (Figure S39, Supporting Information). The EIS results show the smallest charge transfer resistance and diffusion resistance in the high‐frequency region of S/Fe_0.1_Co_2.9_O_4_‐Se, which is beneficial for the electrode to obtain high reversible capacity and adequate cycle stability.^[^ ^54^, ^59^ ^]^

To assess the nucleation/dissociation kinetics of Li_2_S, chronoamperometry measurements were conducted using the Li_2_S_8_/TEG (0.25 m) solution as the electrolyte. As shown in Figure 5f, the S/Fe_0.1_Co_2.9_O_4_‐Se electrode exhibits a higher Li_2_S nucleation capacity of 237 mAh g^−1^ than those of the others, including S/Co_3_O_4_‐Se (112 mAh g^−1^), S/Fe_0.1_Co_2.9_O_4_ (150 mAh g^−1^), and S/Co_3_O_4_ electrodes (83 mAh g^−1^). More importantly, the S/Fe_0.1_Co_2.9_O_4_‐Se electrode showed the fastest rate and shortest Li_2_S nucleation time, if compared to those of the other electrodes. The constants of Li_2_S nucleation and dissociation rates were calculated according to the following equation (4):^[^ ^19^, ^60^ ^]^

(4) $$Ak^{2}=2/\left(\pi t_{m}^{3}\right)$$

where A is the nucleation rate, k is the growth rate, and t_m_ is the time taken to reach the peak current. A higher Ak^2^ value indicates faster Li_2_S nucleation and dissociation processes. As can be seen from Figure 5l, the obtained Ak^2^ value of Fe_0.1_Co_2.9_O_4_‐Se is significantly larger than those of Co_3_O_4_‐Se (3.8*10^−12^S^−3^), Fe_0.1_Co_2.9_O_4_ (2.8*10^−12^S^−3^), and Co_3_O_4_ (1.6*10^−12^S^−3^). Therefore, it can be confirmed that the Fe_0.1_Co_2.9_O_4_‐Se has achieved the fastest nucleation rate and the highest deposition capacity. These results clearly prove that the Fe_0.1_Co_2.9_O_4_‐Se catalyst has the best Li_2_S deposition kinetics, and can effectively reduce the overpotential of initial Li_2_S nucleation and achieve rapid Li_2_S growth kinetics. The morphologies of Li_2_S precipitation on the surface of different cathode materials were further examined using the SEM. As shown in Figure S40 (Supporting Information), the uniform Li_2_S was observed on the surface of Fe_0.1_Co_2.9_O_4_‐Se, whereas the Li_2_S was aggregated seriously on the surfaces of Co_3_O_4_‐Se, Fe_0.1_Co_2.9_O_4_, and Co_3_O_4_.

Fundamentally, the Li_2_S deposition mode is related to the deposition kinetics, which determines the electrochemical performance and reversibility of the battery.^[^ ^61^ ^]^ To unravel the influences of different catalysts on Li_2_S deposition in detail, we performed a dimensionless analysis of the current‐time curves based on the Scharifker‐Hills model.^[^ ^62^ ^]^ As is well known, the nucleation rates for the 2D progressive (2DP) and 2D instantaneous (2DI) processes are controlled by the crystal phase. In contrast, the ion diffusion predominantly determines the rates for 3D progressive (3DP) and 3D instantaneous (3DI) nucleation, potentially enabling a significant enhancement of the Li_2_S nucleation rate.^[^ ^63^ ^]^ As shown in Figure 5g–j, the nucleation of Li_2_S on Co_3_O_4_ electrodes exhibits a 2DP mode, that on the Fe_0.1_Co_2.9_O_4_ shows a 2DI mode for Li_2_S nucleation, and that on the Co_3_O_4_‐Se exhibits a mixed mode of 2DI and 3DP nucleation for Li_2_S. When co‐doped with both Fe and Se, the Fe_0.1_Co_2.9_O_4_‐Se exhibits a 3DI mode for Li_2_S nucleation. These results clearly indicate that Fe/Se co‐doping endows the Fe_0.1_Co_2.9_O_4_‐Se surface with high conductivity and abundant defect sites, which is beneficial to the radial growth of Li_2_S and a balanced lateral atomic diffusion. This further illustrates that the Fe_0.1_Co_2.9_O_4_‐Se catalyst not only increases the adsorption of LiPSs but also improves the redox reaction kinetics, promoting the deposition of Li_2_S.

Beyond the nucleation control, oxidative dissolution dynamics of Li_2_S critically determine the cycling stability of the battery by preventing active‐site passivation. Rapid oxidative dissolution of Li_2_S during the charging process can avoid the accumulation of dead Li_2_S and the severe blockage of active sites. As shown in Figure 5k, the Li_2_S's dissolution capacity for the Fe_0.1_Co_2.9_O_4_‐Se is 822 mAh g^−1^, which is significantly higher than those for the catalysts of Co_3_O_4_‐Se (446 mAh g^−1^), Fe_0.1_Co_2.9_O_4_ (659 mAh g^−1^), and Co_3_O_4_ (354 mAh g^−1^). Phase evolution revealed from the XRD measurement (Figure 5m) confirms that the distinct Li_2_S signals at two theta values of 27° and 31.5° (JCPDS No. 023‐0369) are completely disappeared after the dissolution stage, indicating that the reversible conversion reaction of Li_2_S has been promoted on the Fe_0.1_Co_2.9_O_4_‐Se. According to SEM images obtained from the Li_2_S post‐dissolution catalyst surfaces (Figure S41, Supporting Information), the Li_2_S dissolution on the Fe_0.1_Co_2.9_O_4_‐Se appears the most complete one, without showing the obvious Li_2_S particles. This is followed by Co_3_O_4_‐Se and Fe_0.1_Co_2.9_O_4_, wherein the Li_2_S dissociation ability on the Co_3_O_4_ is much worse. The calculated Ak^2^ values (Figure 5n) further verify the superiority Li_2_S dissolution kinetics of the Fe_0.1_Co_2.9_O_4_‐Se (8×10^−12^ S^−3^), which is greater than those of the Co_3_O_4_‐Se (3.8×10^−12^S^−3^), Fe_0.1_Co_2.9_O_4_ (2.6×10^−12^S^−3^), and Co_3_O_4_ (1.5×10^−12^S^−3^). It is also worth noting that, in the process of Li_2_S deposition/dissolution, the Fe‐doping is beneficial in improving the capacity of Li_2_S deposition/dissolution, whereas the Se─O coordination is more conducive in reducing the initial overpotential of Li_2_S deposition/dissolution and increasing the reaction rate, if comparing with those using the Co_3_O_4_‐Se and Fe_0.1_Co_2.9_O_4_ electrodes. Therefore, the Fe/Se co‐doping enables the Fe_0.1_Co_2.9_O_4_‐Se catalyst with excellent bidirectional catalytic performance. This characteristic can be further validated by the potentiostatic intermittent titration technique (PITT) measurement results. As the results shown in Figures S42 and S43 (Supporting Information), whether during the discharge or charge process, the Fe_0.1_Co_2.9_O_4_‐Se exhibits a higher peak current and an earlier peak time if compared to those of S/Co_3_O_4_‐Se, S/Fe_0.1_Co_2.9_O_4_, and S/Co_3_O_4_, indicating its faster reaction rate for the Li_2_S deposition/dissolution.

### 2.4 Electrochemical Performance

**Figure 6:**
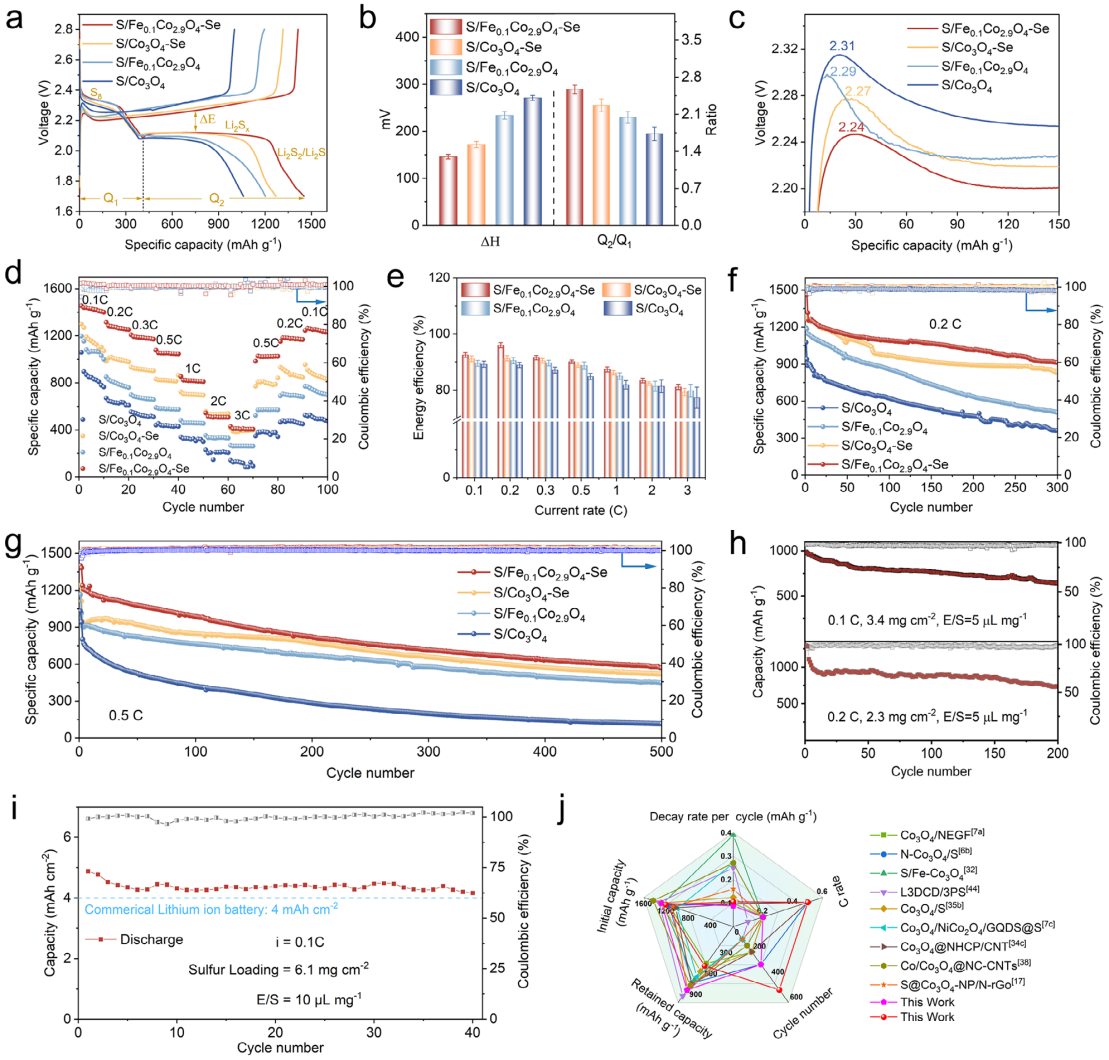
a) GCD curves of different electrodes at a current rate of 0.1 C. b) Polarization voltage and Q_2_/Q_1_ ratio (Error bars denote the standard deviation from measurements for cathodes obtained from three independent coin cells), and c) enlarged charge profiles for S/Fe_0.1_Co_2.9_O_4_‐Se, S/Co_3_O_4_‐Se, S/Fe_0.1_Co_2.9_O_4_, and S/Co_3_O_4_ based Li─S batteries. d) Rate performance and e) corresponding energy efficiency of several electrodes. Cycling stability of all electrodes at f) 0.2 C and g) 0.5 C. h) Cycling performance of the S/Fe_0.1_Co_2.9_O_4_‐Se‐based Li─S battery with high‐sulfur loading and low E/S ratio. i) Cycling performance of Li─S pouch cell assembled with S/Fe_0.1_Co_2.9_O_4_‐Se‐based Li─S battery at 0.1 C. j) Radar chart of S/Fe_0.1_Co_2.9_O_4_‐Se cells in comparison with the reported representatives.

Electrochemical performance of the S/Fe_0.1_Co_2.9_O_4_‐Se catalyst as the LSBs’ cathode was evaluated using asymmetric coin cells. **Figure** **6**a shows the first cycle galvanostatic charge‐discharge profiles recorded at a current density of 0.1C (1 C = 1675 mAh g^−1^) within the operational voltage window of 1.7–2.8 V (vs Li^+^/Li). The polarization potential (ΔE), determined by the voltage gap between the oxidation plateau and the secondary reduction plateau for the Li‐S redox reactions, demonstrates a significant improvement in the S/Fe_0.1_Co_2.9_O_4_‐Se electrode.^[^ ^64^ ^]^ With a remarkably low ΔE value of 146 mV, which is substantially smaller than those of the comparative electrodes, the S/Fe_0.1_Co_2.9_O_4_‐Se exhibits enhanced catalytic activity toward the LiPSs conversion. Here Q_1_ and Q_2_ represent the capacities generated at the dual discharge plateaus at 2.3 and 2.1 V, respectively. The Q_2_/Q_1_ ratio can be served as a key indicator for the LiPSs’ conversion efficiency, where a higher ratio value reflects its better catalytic capability.^[^ ^18^ ^]^ As illustrated in Figure 6b, the S/Fe_0.1_Co_2.9_O_4_‐Se electrode achieves an exceptional Q_2_/Q_1_ ratio of 2.57, outperforming those of the control samples. This performance enhancement is further corroborated by the minimized charging overpotential of S/Fe_0.1_Co_2.9_O_4_‐Se electrode (Figure 6c), suggesting the accelerated electrochemical kinetics during the oxidation of Li_2_S. The results of rate capability (Figure 6d) and the corresponding galvanostatic charge‐discharge profiles (Figure S44, Supporting Information) demonstrate the excellent reversible capacity of S/Fe_0.1_Co_2.9_O_4_‐Se. The S/Fe_0.1_Co_2.9_O_4_‐Se maintains its specific capacities at values of 1432, 1283, 1192, 1077, 853, 544, and 429 mAh g^−1^ with the increased current densities from 0.1 to 3 C. In particular, a distinctly high voltage plateau can be obtained even at 3 C, confirming its rapid LiPS conversion kinetics. Upon reverting to 0.1 C, the specific capacity value of the electrode has been recovered to 1243 mAh g^−1^, demonstrating its outstanding reversibility and stability. If compared with those results of the S/Se‐Fe_0.1_Co_2.9_O_4_ (Figure S45, Supporting Information), for the Fe_0.1_Co_2.9_O_4_‐Se, the Se atoms can be selectively coordinated with oxygen (but not with O_v_), and thus provide more effective catalytic conversion of LiPSs than those of Se located in the O_v_ sites to coordinate with metal/oxygen ions. This difference can be further quantified through Gibbs free energy calculations (Figures S46 and S47, Supporting Information), where the rate‐determining step from Li_2_S_4_ reduction to Li_2_S exhibits lower energy barriers on Fe_0.1_Co_2.9_O_4_‐Se (0.15/0.05 eV) versus Se‐Fe_0.1_Co_2.9_O_4_ (0.47/0.06 eV). All the above results indicate that the Se─O coordination optimizes the catalytic surfaces for the LiPSs’ redox process.

Energy efficiency (E) is a critical parameter for evaluating the conversion efficiency of energy storage devices (E = ∫ UI dt), especially when they are required to operate at high current densities.^[^ ^37^ ^]^ As depicted in Figure 6e, the S/Fe_0.1_Co_2.9_O_4_‐Se electrode demonstrates its superior energy efficiency, attributed to its minimized polarization potential. Long‐term cycling stability was evaluated at 0.2 C (Figure 6f), where the S/Fe_0.1_Co_2.9_O_4_‐Se cathode maintains a value of 920 mAh g^−1^ after 300 cycles with an ultralow capacity fade rate of 0.089 % per cycle. This performance significantly surpasses those of the control group electrodes, including S/Co_3_O_4_‐Se (849 mAh g^−1^, 0.107%), S/Fe_0.1_Co_2.9_O_4_ (521 mAh g^−1^, 0.181%), and S/Co_3_O_4_ (364 mAh g^−1^, 0.199%). When subjected to extended cycling at 0.5 C (Figure 6g), the S/Fe_0.1_Co_2.9_O_4_‐Se battery delivers an initial capacity of 1206 mAh g^−1^, stabilizing at 570 mAh g^−1^ after 500 cycles (0.1054% fade/cycle). In contrast, the capacities of S/Co_3_O_4_‐Se, S/Fe_0.1_Co_2.9_O_4_, and S/Co_3_O_4_ electrodes become decayed quickly to 520, 450, and 117 mAh g^−1^ after 500 cycles, respectively, with their corresponding capacity decay rates per cycle of 0.103, 0.116, and 0.170 %, respectively. This enhanced stability is mainly originates from the dual functionality of Fe_0.1_Co_2.9_O_4_‐Se, which can suppress the LiPSs’ shuttle effect and accelerate the conversion kinetics, thereby significantly mitigating the loss of active material. Remarkably, under a high current of 1 C (Figure S48, Supporting Information), the S/Fe_0.1_Co_2.9_O_4_‐Se achieves a near‐unity coulombic efficiency (≈100%) with a capacity fade rate of 0.1171%, after cycling over 500 cycles. The S/Fe_0.1_Co_2.9_O_4_‐Se electrode shows an initial peak capacity of 637 mAh g^−1^ at a current density of 2 C, and then maintains a capacity retention rate of 87% after 300 cycles. In comparison, the capacity retention rates for those electrodes of S/Fe_0.1_Co_2.9_O_4_, S/Co_3_O_4_‐Se, and S/Co_3_O_4_ are merely 57%, 58%, and 64%, respectively (Figure S49, Supporting Information). The results demonstrate the outstanding catalytic activity of the Fe_0.1_Co_2.9_O_4_‐Se.

Post‐cycling (500 cycles at 0.5 C) SEM characterization (Figure S50, Supporting Information) reveals that there are dramatic structural differences. The cathode surface of S/Fe_0.1_Co_2.9_O_4_‐Se‐based battery is relatively flat without showing obvious cracks, and its corresponding lithium anode displays only slight corrosion. In stark contrast, the batteries made using the other electrode materials in this study exhibit severe roughening of the cathode and cracking of the anode, both of which are correlated to their inferior electrochemical performance.^[^ ^65^ ^]^ It clearly indicates that the S/Fe_0.1_Co_2.9_O_4_‐Se cathode can effectively suppress the shuttle effect.

To further demonstrate the superior catalytic activity of S/Fe_0.1_Co_2.9_O_4_‐Se for the practical applications of LSBs, the S/Fe_0.1_Co_2.9_O_4_‐Se electrode was studied with a sulfur loading of 3.4 mg cm^−2^ and an electrolyte‐to‐sulfur (E/S) ratio of 5 µL mg^−1^. Results shown in Figure 6h reveal that it can achieve a specific capacity of 645 mAh g^−1^ after 200 cycles, with a capacity decay rate of 0.164 % per cycle. The exceptional cycling stability under a high sulfur loading was further validated using the assembled pouch cells. With a sulfur loading of 6.1 mg cm^−2^ and an E/S ratio of 10 µL mg^−1^, the S/Fe_0.1_Co_2.9_O_4_‐Se‐based pouch cell exhibited stable cycling over 40 cycles at 0.1 C, delivering a remarkable areal capacity of 4.8 mAh cm^−2^. Notably, this value significantly surpasses the areal capacity (≈4 mAh cm^−2^) of those commercial lithium‐ion batteries, demonstrating its excellent sustained power supply capability for electronic devices (Figure 6i and Figure S51, Supporting Information). We have compared this value against those of Co_3_O_4_‐based LSBs reported in the past three years (Figure 6j). The S/Fe_0.1_Co_2.9_O_4_‐Se in this study exhibits distinct advantages in terms of cycle number, C rate, initial capacity, capacity retention, and capacity decay rate per cycle, as shown in Figure 6j and Table S6 (Supporting Information). Thus, the Fe_0.1_Co_2.9_O_4_‐Se effectively addresses the dual challenges for suppression of LiPSs’ shuttle effect and enhancement of reaction kinetics, providing a good method for improving sulfur utilization.

### 2.5 In Situ Experiment of S_8_ and Li_2_S Reaction

**Figure 7:**
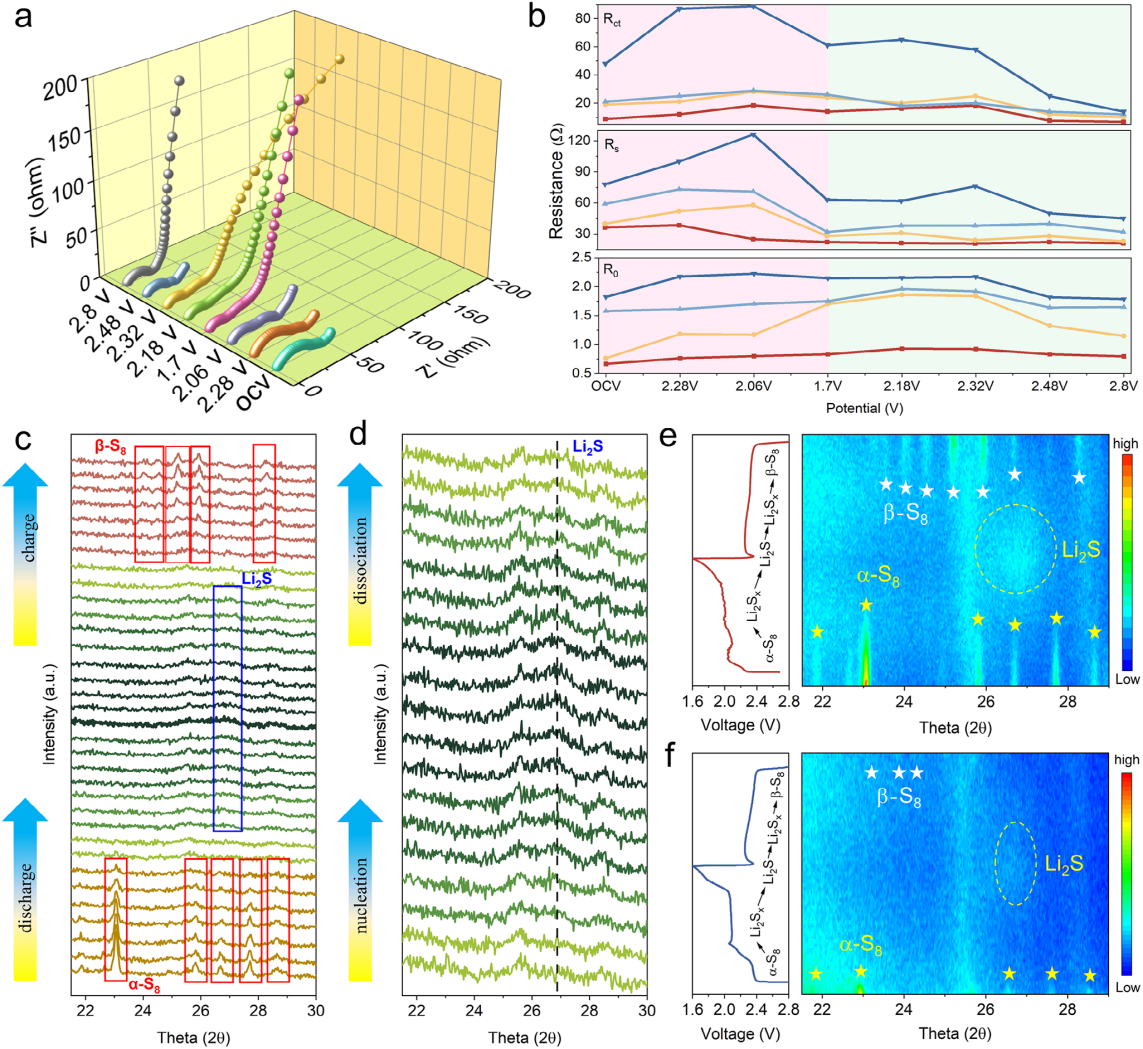
a) In situ EIS spectra of S/Fe_0.1_Co_2.9_O_4_‐Se cathode. b) The change of R_0_, R_s_, R_ct_ with different discharge/charge stages. c) In situ XRD patterns of S/Fe_0.1_Co_2.9_O_4_‐Se in the first cycle at 0.1 C rate. d) Enlarged in situ XRD curve of S/Fe_0.1_Co_2.9_O_4_‐Se in the nucleation and dissociation region of Li_2_S. In situ XRD contour plots for e) S/Fe_0.1_Co_2.9_O_4_‐Se cathode and f) S/Co_3_O_4_ cathode.

To determine the underlying mechanisms governing the divergent electrochemical performances and particularly the LiPSs diffusion‐deposition dynamics, the EIS was performed to evaluate the resistance of the electrodes, interfaces, and electrolyte (**Figures** **7**a and S52, Supporting Information). All the EIS plots consist of a high‐frequency intercept, a high‐frequency semicircle, a high‐to‐medium frequency semicircle, and a low‐frequency sloping tail. These represent the external cell connection and internal resistance of the electrolyte (R_0_), the solid electrolyte interface resistance (R_s_), the charge transfer resistance (R_ct_), and the diffusion impedance, respectively.^[^ ^66^ ^]^ As illustrated in Figure 7b, the R_0_ evolution during the discharge process exhibits distinct patterns for various electrodes. For those of the S/Co_3_O_4_‐Se, S/Fe_0.1_Co_2.9_O_4_, and S/Co_3_O_4_, R_0_ displays a characteristic trend of initial increase but then a decrease trajectory, correlating with the formation and conversion of soluble LiPSs in the electrolyte.

As the discharge proceeds, the initial R_0_ is increased due to the increase of electrolyte's viscosity induced by the conversion from S_8_ into the soluble LiPSs (i.e., Li_2_S_x_, 4≤x≤8). The subsequent R_0_ reduction reflects the decrease of viscosity as the insoluble Li_2_S_2_/Li_2_S becomes precipitated, and the charging process changes to the opposite direction. However, the S/Fe_0.1_Co_2.9_O_4_‐Se electrode maintains a stable R_0_ value throughout cycling, indicating its effective suppression of LiPSs’ dissolution through strong chemisorption in the electrolyte.^[^ ^67^ ^]^ The changes in values of R_s_ and R_ct_ are linked to the formation and decomposition of Li_2_S_2_/Li_2_S. As the reduction of S_8_ proceeds, the insulating Li_2_S_2_/Li_2_S becomes accumulated and covers the electrode's surface, thereby increasing the charge transfer resistance. Whereas the S/Fe_0.1_Co_2.9_O_4_‐Se electrode exhibits minor changes in its R_s_ and R_ct_ values if compared to those of the S/Co_3_O_4_‐Se, S/Fe_0.1_Co_2.9_O_4_, and S/Co_3_O_4_ electrodes. This can be attributed to the uniform deposition and rapid conversion of Li_2_S for the S/Fe_0.1_Co_2.9_O_4_‐Se, avoiding the formation of dead sulfur.^[^ ^66^ ^]^ All the above results have demonstrated the efficient adsorption and catalytic conversion effect of S/Fe_0.1_Co_2.9_O_4_‐Se for LiPSs.

To gain deeper insights into phase evolution dynamics, in situ XRD was used to monitor real‐time sulfur speciation during the redox processes catalyzed by S/Fe_0.1_Co_2.9_O_4_‐Se (Figure 7c,d). A series of sharp α‐S_8_ crystal diffraction peaks (JCPDS No. 78‐1889) can be obtained in the S/Fe_0.1_Co_2.9_O_4_‐Se cathode during the initial stage of the first cycle. However, these are gradually decreased and then totally disappeared as the discharge process is progressed. Subsequently, a characteristic peak of Li_2_S (JCPDS No. 77‐2145) has appeared at ≈27°, indicating the effective conversion of α‐S_8_ to Li_2_S. During the following charging process, the Li_2_S peak becomes gradually disappeared, and the β‐S_8_ peaks can be observed at the end of charging process, demonstrating the reversibility of solid‐liquid conversion in the Li─S battery. Figure 7e shows the corresponding XRD contour evolution plot, clearly displaying the identifiable signals of sulfur species from the S/Fe_0.1_Co_2.9_O_4_‐Se electrode. In contrast, under the same charge‐discharge conditions, the S/Co_3_O_4_ cathode (Figure 7f) exhibits the reduced Li_2_S and β‐S_8_ signals, indicating its lower utilization of the active material and poor sulfur conversion.^[^ ^68^ ^]^

## 3 Conclusion

In conclusion, a novel Fe‐doped Co_3_O_4_ with Se‐O coordination porous nano‐electrocatalyst (Fe_0.1_Co_2.9_O_4_‐Se) has been successfully prepared via an etching‐carbonization process. The unique porous structure ensures rapid ion/mass transport, exposes abundant active sites, and provides sufficient sulfur storage space. Experimental results demonstrate that such the Fe/Se co‐doping strategy induces 3D instantaneous nucleation of Li_2_S on Fe_0.1_Co_2.9_O_4_‐Se, accelerates the ion/electron transfer of Li_2_S, and exhibits strong adsorption and bidirectional catalysis during the sulfur redox process. Theoretical calculations reveal that this behavior arises from the redistribution of the electronic structure of Co_3_O_4_ caused by Fe and Se co‐doping, resulting in a narrowed bandgap and an upward shift of the d‐band center. Therefore, the conductivity of Fe_0.1_Co_2.9_O_4_‐Se is enhanced, the adsorption energy for LiPSs is improved, and the energy barrier for LiPSs conversion is significantly reduced. In addition, experiments and theoretical investigations reveal that the Se coordinates with O excluding O_v_ via Se‐O bonds in the Fe_0.1_Co_2.9_O_4_‐Se structure exhibits a much lower energy barrier for the LiPSs’ conversion if compared to the Se located in O_v_ sites to coordinate with metal/oxygen ions structure in Se‐Fe_0.1_Co_2.9_O_4_. Consequently, the S/Fe_0.1_Co_2.9_O_4_‐Se cathode exhibits a superior electrochemical performance of LSBs. It delivers an impressive initial capacity of 1432 mAh g^−1^ at 0.1 C with an ultralow capacity decay rate of 0.1054% per cycle over 500 cycles at 0.5 C. More importantly, using the pouch cell and with a high sulfur loading (6.1 mg cm^−2^) and a limited electrolyte (E/S = 10 µL mg^−1^), it maintains a remarkable areal capacity of 4.8 mAh cm^−2^ after 40 cycles. This work establishes a novel paradigm for electronic structure engineering, providing valuable insights for designing high‐performance LSBs electrocatalysts with great practical potential.

## Conflict of Interest

The authors declare no conflict of interest.

## Supporting information



Supporting Information

## Data Availability

The data that support the findings of this study are available from the corresponding author upon reasonable request.
