# Supplementary material for: Exclusive Se‐O Coordination and Fe‐doping Complementation: A Catalytic Strategy for Enhanced Sulfur Redox in Li‐S Batteries
Source: Adv Sci (Weinh). 2025 Nov 29;13(9):e13049. doi: 10.1002/advs.202513049 (PMC12904079; doi:10.1002/advs.202513049)
Supplement: Supplementary file 1 — Supporting Information [file ADVS-13-e13049-s001.pdf]

Supporting Information for

Exclusive Se-O Coordination and Fe-doping Complementation: A Catalytic Strategy for Enhanced Sulfur Redox in Li-S Batteries

*Zhao Yang, Yu Wang, Jingchen Han, Tong Wu, Yongqing Fu**, *Qingsheng Wu, and Ming Wen**

Z. Yang, Y. Wang, J. Han, Prof. T. Wu, Prof. Q. Wu, Prof. M Wen
School of Chemical Science and Engineering, Shanghai Key Laboratory of Chemical Assessment and Sustainability, Tongji University
Shanghai 200092, China
E-mail: m_wen@tongji.edu.cn

Prof. Richard Y.Q. Fu
Faculty of Engineering and Environment
Northumbria University
Newcastle upon Tyne NE99, UK
E-mail: richard.fu@northumbria.ac.uk

I Experimental Section

1. Chemicals

Sulfur powder (S, ≥99.5%), ethanol (C_2H_5OH, ≥99.7%), and methanol (CH_3OH, ≥99.5%) were purchased from Sinopharm Chemical Reagent Co., Ltd. Iron (III) nitrate nonahydrate ($Fe(NO_3)_2·9H_2O$, 99%), cobalt nitrate hexahydrate ($Co(NO_3)_2·6H_2O$, 99%), selenium dioxide (SeO_2, 99%), 2-methylimidazole ($C_4H_6N_2$, 2-MIM, 98%), and N-methyl pyrrolidone (NMP) were purchased from Aladdin Reagent Co. Ltd. (China). Ketjen Black, polyvinylidene fluoride (PVDF), lithium sulfide (Li_2S), 1,2-dimethoxyethane (DME), 1,3-dioxolane (DOL), and Lithium bistrifluoromethane sulfonimide (LiTFSI) were purchased from KeLuDe (Dongguan, China). All the chemical reagents were used in their as-received states without further purification.

2. Synthesis of the Fe/ZIF-67 and ZIF-67

Typically, 2-methylimidazole (6.49 g) was dissolved in methanol (200 mL) to form the solution A. Co ($NO_3)_2·6H_2O$ (2.93 g) was dissolved in methanol (200 mL) to form the solution B. Then, the solution A was rapidly transferred to the solution B under magnetic stirring. The resulting mixed solution was stirred at room temperature for 8 h, and the precipitate is collected by centrifugation at 10000 rpm, washed with methanol for 5 times, and dried in a vacuum oven overnight to obtain the product of ZIF-67. The synthesis process of Fe/ZIF-67 was the same as that of ZIF-67, except that the solution B is 200 mL of methanol dissolved with 2.62 g of Co ($NO_3)_2·6H_2O$ and 0.404 g of Fe ($NO_3)_2·9H_2O$.

3. Synthesis of the FeCo-O-Se and Co-O-Se

100 mg of Fe/ZIF-67 and ZIF-67 were dissolved in 40 mL of methanol and sonicated to form solution A. 50 mg of SeO_2 was dissolved in 80 mL of water and sonicated to form solution B. The solution B was added into the solution A, stirred for 2 min, and allowed to stand for 1 h. The precipitate is collected by centrifugation at 10000 rpm, washed, and vacuum-dried at 60 °C overnight. The resulting products were named as FeCo-O-Se and Co-O-Se, respectively.

4. Synthesis of $Fe_{0.1}Co_{2.9}O_4$-Se, Co_3O_4-Se, $Fe_{0.1}Co_{2.9}O_4$, and Co_3O_4

In brief, FeCo-O-Se and Co-O-Se were placed in a ceramic boat. Then, the samples

were heated from room temperature to 550 ºC at a rate of 2 ºC min^{-1} and kept at that temperature for 2 h under Ar. After naturally cooling down to room temperature, black powders were formed, which were denoted as $Fe_{0.1}Co_{2.9}O_4$-Se and Co_3O_4-Se, respectively. Fe/ZIF-67 and ZIF-67 were placed in a ceramic boat and heated from room temperature to 550 °C at a rate of 2 ºC min^{-1}, then held at that temperature for 0.5 h under an Ar atmosphere, and cooled down to room temperature. Subsequently, they were heated in air to 350 ºC at a rate of 2 ºC min^{-1} and held for 2 h, then cooled down to room temperature to obtain $Fe_{0.1}Co_{2.9}O_4$ and Co_3O_4, respectively.

5. Synthesis of the Se-$Fe_{0.1}Co_{2.9}O_4$

$Fe_{0.1}Co_{2.9}O_4$ and Se powder were placed in separate ceramic boats, with the Se powder placed upstream and the $Fe_{0.1}Co_{2.9}O_4$ placed downstream. The mixture was heated to 350 ºC at a heating rate of 2 ºC min^{-1} under an Ar atmosphere and reacted for 2 h to synthesize Se-$Fe_{0.1}Co_{2.9}O_4$.

6. Synthesis of the S/$Fe_{0.1}Co_{2.9}O_4$-Se, S/Co_3O_4-Se, S/$Fe_{0.1}Co_{2.9}O_4$, S/Co_3O_4 and S/Se-$Fe_{0.1}Co_{2.9}O_4$

Sulfur was incorporated by utilizing a melting diffusion process. To obtain the S/$Fe_{0.1}Co_{2.9}O_4$-Se, the prepared $Fe_{0.1}Co_{2.9}O_4$-Se was mixed with sulfur (99.98%, Sigma-Aldrich) in a 4:1 ratio and heated in a glass bottle at 155 °C Ar for 12 h. The S/Co_3O_4-Se, S/$Fe_{0.1}Co_{2.9}O_4$, S/Co_3O_4 and S/Se-$Fe_{0.1}Co_{2.9}O_4$ was also prepared using the same method, except using different catalyst materials.

7. Materials Characterization

The morphology of the synthesized materials was examined using a scanning electron microscope (SEM, JEOL S4800, Japan). A transmission electron microscope (TEM, JEOL, JEM-2100EX) was used to investigate the internal crystal structures of the as-prepared samples. High-angle annular darkfield scanning TEM (HAADF-STEM) images and elemental mappings were collected using an equipment of JEM-ARM200F at an acceleration voltage of 200 KV. Crystalline structure analysis was performed using a Bruker D8 Focus X-ray diffractometer (XRD) equipped with Cu Kα radiation (40 KV/40 mA). The Raman spectra were obtained using a RENISHAW Via Raman Microscope with a laser wavelength of 325 nm. The N_2 adsorption/desorption isotherms

were obtained using the Ankersmid Belsorp-Max method, and the Brunauer-Emmett-Teller (BET) method was used to assess the surface area and pore size distribution, respectively. Surface elemental compositions and binding energies were determined by X-ray photoelectron spectroscopy (XPS; Thermo Scientific K-Alpha) using a monochromatic Al Kα X-ray source.

8. DFT calculations

First-principles calculations were performed within the plane-wave density functional theory (DFT) framework as implemented in the Quantum Espresso.[1] The exchange-correlation effects were described using the Perdew-Burke-Ernzerhof generalized gradient approximation (GGA-PBE).[2] To properly incorporate long-range van der Waals (vdW) interactions, the DFT-D2 dispersion correction method by Grimme was employed.[3] The bottom two atomic layers of the (311) surface models were fixed to mimic the bulk-like behavior. In structural relaxation, the energy cutoff for the plane-wave basis expansion and convergence threshold for the force on each relaxed atom were set to be 50 Ry and 0.02 eV $Å^{-1}$, respectively. A 2×2×1 Monkhorst-Pack k-point set-up was used for the optimization of adsorbed Li_xS_y and a vacuum slab of 16 Å was used for surface isolation to prevent interaction between two units. The free energy of adsorbates was calculated using $G=E_{DFT}+E_{ZPE}-TS$, where E_{DFT} is the electronic energy calculated by DFT, E_{ZPE} is the zero-point energy (ZPE), T and S are the temperature and entropy, respectively.

9. Lithium polysulfide visualized adsorption tests

All the samples were dried at 25 ºC for 8 h before the adsorption tests. Li_2S_6 solution was prepared by dissolving an appropriate amount of sulfur and Li_2S in a 1,3-dioxolane/1,2-dimethoxyethane (DOL/DME) solution with a volumetric ratio of 1:1. The solution was then stirred at 80 ºC in an Ar-filled glove box for overnight to produce a Li_2S_6 solution (0.5 M). The Li_2S_6 solution was then diluted to 5 mM with equal volumes of DOL/DME, prepared for the polysulfide adsorption tests. Subsequently, 10 mg of each material was added to separate vials containing 5 mL of the 5 mM Li_2S_6 solution. Changes in the supernatant color were recorded over time.

10. Symmetrical cell assembly and measurements

Symmetric electrochemical cells were assembled using two identical electrodes. The electrode was prepared by mixing $Fe_{0.1}Co_{2.9}O_4$-Se, Co_3O_4-Se, $Fe_{0.1}Co_{2.9}O_4$, or Co_3O_4, Ketjen Black, and polyvinylidene fluoride (PVDF) with a mass ratio of 8:1:1 in NMP followed by coating the slurry onto an Al foil. The Celgard 2500 was used as the separator. 0.5 M Li_2S_6 electrolyte was prepared by dissolving appropriate amounts of sulfur and Li_2S in the blank electrolyte (1 M LiTFSI in DOL/DME (1:1 by volume)) and stirred at 70 ºC in an Ar-filled glove box for overnight. 40 μL and 0.5 M Li_2S_6 electrolyte was dropped into the CR2032 coin cell. Cyclic voltammetry (CV) was performed at 5 mV s^{-1} between -0.8 to 0.8 V using an electrochemical workstation (Chenhua, China, CHI600E).

11. Li_2S nucleation and dissolution tests

Components of S and Li_2S (with their molar ratio of 7:1) and 1.0 M LiTFSI were dissolved in a tetraethylene glycol dimethyl ether solution by stirring for 24 h to obtain the Li_2S_8 solution (0.2 mol L^{-1}). $Fe_{0.1}Co_{2.9}O_4$-Se, Co_3O_4-Se, $Fe_{0.1}Co_{2.9}O_4$, and Co_3O_4 were used as the working electrodes. 20 μL of Li_2S_8 solution was deposited onto the working electrode, and then 20 μL of a 1.0 M LiTFSI solution without Li_2S_8 was dropped onto the lithium anode. The batteries were discharged to 2.06 V at 0.112 mA and then kept potentiostatically at 2.05 V for the Li_2S to be nucleated and grown until the current was below 10^{-5} A. The nucleation rate of Li_2S on the substrates was calculated based on the Faraday's Law.

12. potentiostatic intermittent titration technique tests

The coin cells were assembled using $Fe_{0.1}Co_{2.9}O_4$-Se, Co_3O_4-Se, $Fe_{0.1}Co_{2.9}O_4$, and Co_3O_4 as cathodes, lithium foil (with a diameter of 16 mm) as the anode, and polypropylene as the separator. The Li_2S_8 solution consisted of 40 μL of 0.5 mol L^{-1} was used as catholyte. The anode side was supplemented with 5 μL of blank electrolyte (1.0 mol L^{-1} LiTFSI in DOL/DME [v/v = 1:1]). The total sulfur content in each cell was controlled to be approximately 0.64 mg. Potentiostatic intermittent titration technique (PITT) tests were conducted using a charge-discharge system (New Wei, China). For the discharge PITT evaluation process, the aforementioned cells were first left to stand at open-circuit voltage for 2 h, with the cutoff condition for each step set as a current

less than 30 μA. For the charge PITT evaluation process, the cells were first discharged at a constant current of 0.05 C to 1.7 V, followed by a constant-voltage discharge at 1.7 V for 4 hours to ensure that all electroactive polysulfides were converted to Li_2S. Subsequent steps were carried out in a manner analogous to the discharge PITT procedure, except that the set voltage was gradually increased.

13. Electrochemical characterizations

Standard CR2032-type coin cells were assembled in an Ar-filled glove box with oxygen and moisture content below 1 ppm. The sulfur composite (S/$Fe_{0.1}Co_{2.9}O_4$-Se, S/Co_3O_4-Se, S/$Fe_{0.1}Co_{2.9}O_4$, and S/Co_3O_4), Ketjen Black, and PVDF were well-mixed with a mass ratio of 8:1:1 in NMP to form a slurry. The composite electrodes were fabricated by coating the slurry on the aluminum foil with a mass loading around 1.2 mg cm^{-2}. The lithium foil was used as a counter and reference electrode, while the Celgard 2500 was used as the separator. The electrolyte was prepared by dissolving 1.0 M LiTFSI and 2 wt.% $LiNO_3$ in the mixed solvent of 1,3-dioxolane (DOL) and 1,2-dimethoxyethane (DME) with a volume ratio of 1:1. 40 μL electrolyte was dropped in the CR2032 coin cell. Land 2001A battery testing system was used for the cycling and rate performance between 1.7 and 2.8 V (vs. Li^+/Li). CV and electrochemical impedance spectroscopy (EIS) measurements were tested using the CHI600E electrochemical workstation.

14. Theoretical equations for Current-Time Transients of four classical electrochemical deposition models

2D instantaneous (2DI) nucleation and 2D progressive (2DP) nucleation processes were fitted according to the Bewick, Fleischman, and Thirsk (BFT) models; 3D instantaneous (3DI) nucleation and 3D progressive (3DP) nucleation processes were fitted according to the Scharifker-Hills models (SH). The corresponding formula are as follows (1)(2)(3)(4):

$$\text{2DI:} \frac{j}{j_m} = \left(\frac{t}{t_m}\right)\left\{\exp\left[\frac{t^2-t_m^2}{2t_m^2}\right]\right\} \quad (1)$$

$$\text{2DP:} \frac{j}{j_m} = \left(\frac{t}{t_m}\right)^2\left\{\exp\left[\frac{-2(t^3-t_m^3)}{3t_m^3}\right]\right\} \quad (2)$$

$$3DI: \frac{j}{j_m} = \left(\frac{1.9542}{t/t_m}\right)^{1/2} \left\{1\text{-exp}\left[1.2564\left(\frac{t}{t_m}\right)\right]\right\} \qquad (3)$$

$$3Dp: \frac{j}{j_m} = \left(\frac{1.2254}{t/t_m}\right)^{1/2} \left\{1\text{-exp}\left[2.3367\left(\frac{t}{t_m}\right)^2\right]\right\} \qquad (4)$$

II Supporting Figures

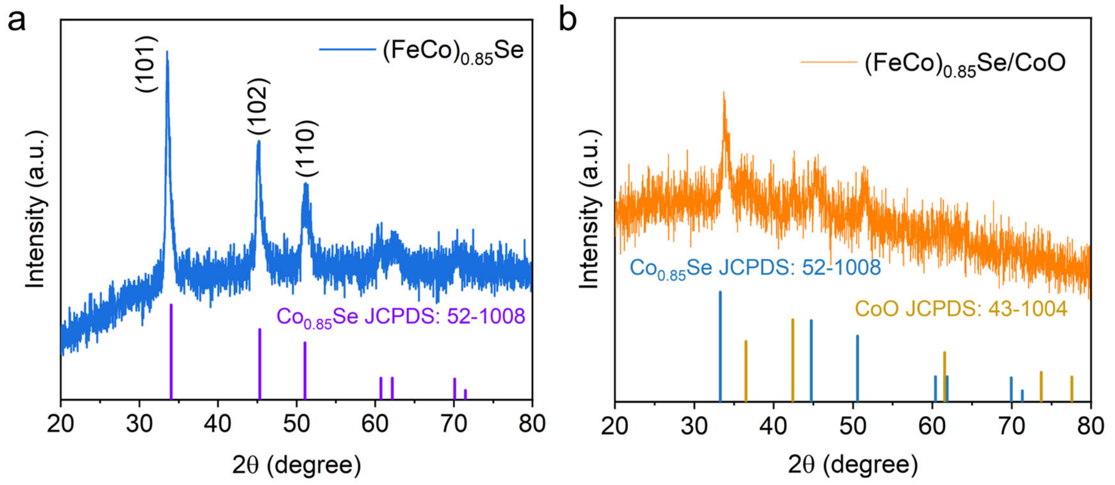

Figure S1. a-b) XRD patterns of the product obtained by regulating the volume ratio of ethanol and deionized water.

Note: By changing the volume ratio of ethanol to water, the polarity of the solvent can be controlled, thereby changing the concentration and hydrolyzability of SeO_3^{2-} in the solution. This change will affect the etching rate of the MOF and the chemical environment of Se, thus determining the composition of the final product based on the reduction ability of SeO_3^{2-} during the final carbonization process.

In Figure S1 a), when the volume ratio of ethanol to water is 3:1, the limited solubility of SeO_2 due to the weak polarity of the reaction solution results in a lower concentration of SeO_3^{2-} and a slower etching rate. This leads to the retention of more carbon matrix after 1 hour of etching, which reduces SeO_3^{2-} to Se^{2-} during calcination and forms $(FeCo)_{0.85}Se$ with the metal. In Figure S1 b), when the volume ratio of ethanol to water is 1:1, the etching rate of Fe-ZIF-67 is moderate. After 1 hour of etching, the residue of organic ligands decreases, and the reducing ability weakens. Therefore, during carbonization, SeO_3^{2-} is not fully reduced, thus forming a heterojunction of $(FeCo)_{0.85}Se/CoO$.

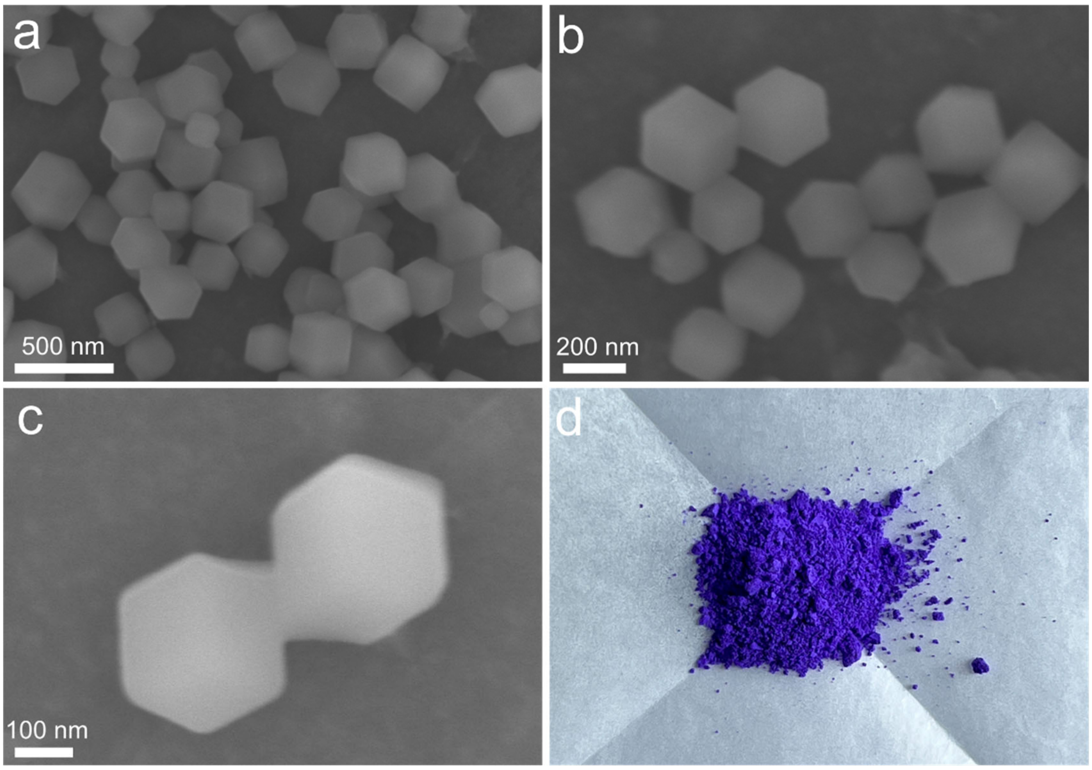

Figure S2. a-c) SEM images of ZIF-67 in different magnifications. d) Photograph of the real material of ZIF-67.

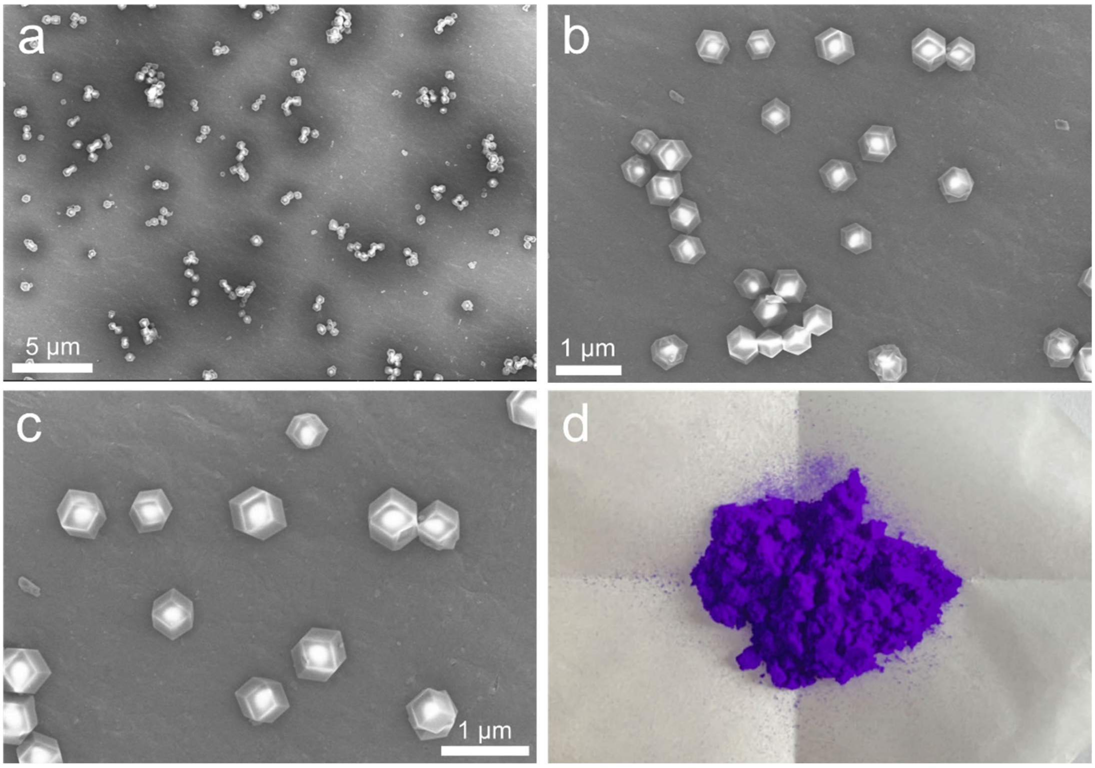

Figure S3. a-c) SEM images of Fe/ZIF-67 in different magnifications. d) Photograph of the real material of Fe/ZIF-67.

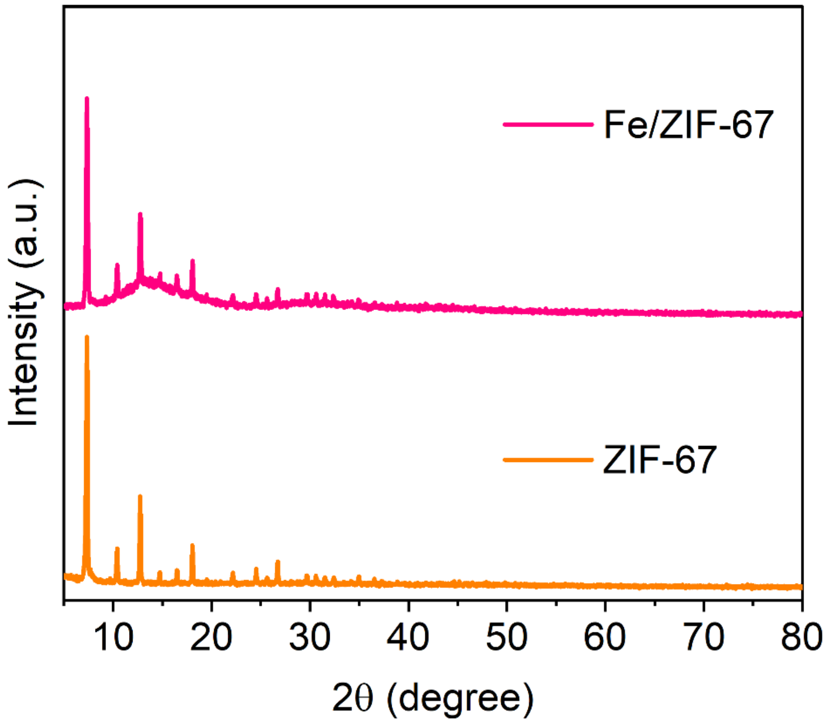

Figure S4. XRD patterns of ZIF-67 and Fe/ZIF-67.

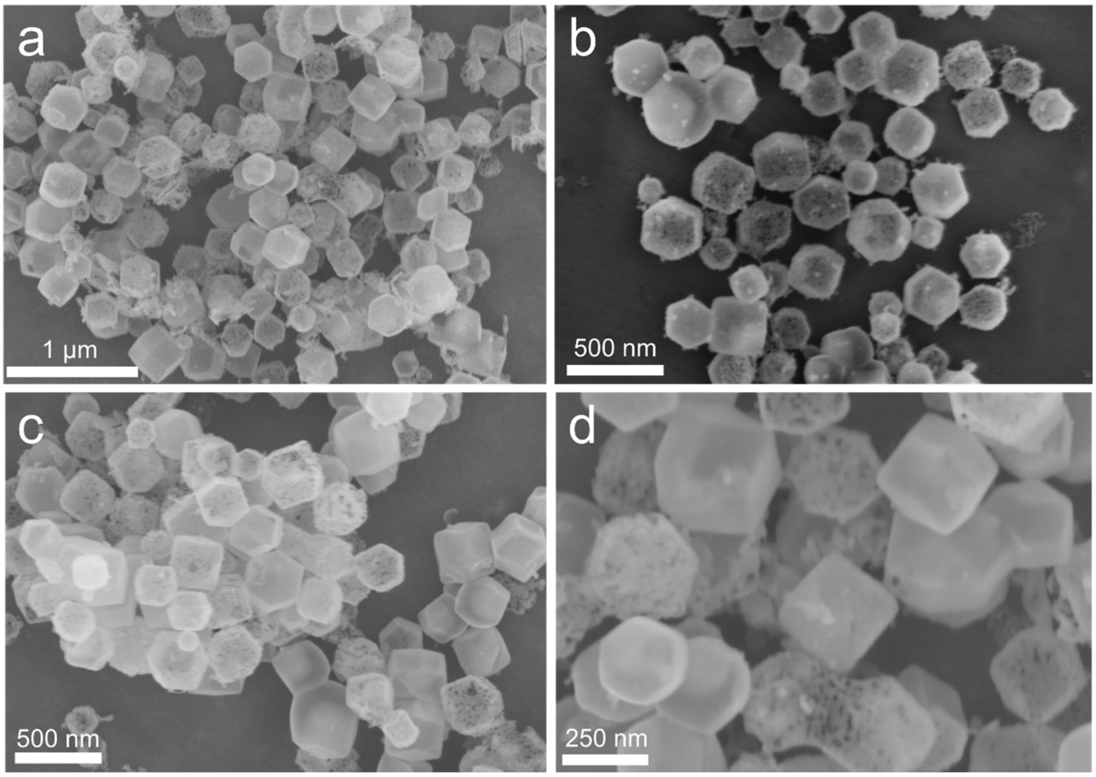

Figure S5. a-d) SEM images of Co-O-Se in different magnifications.

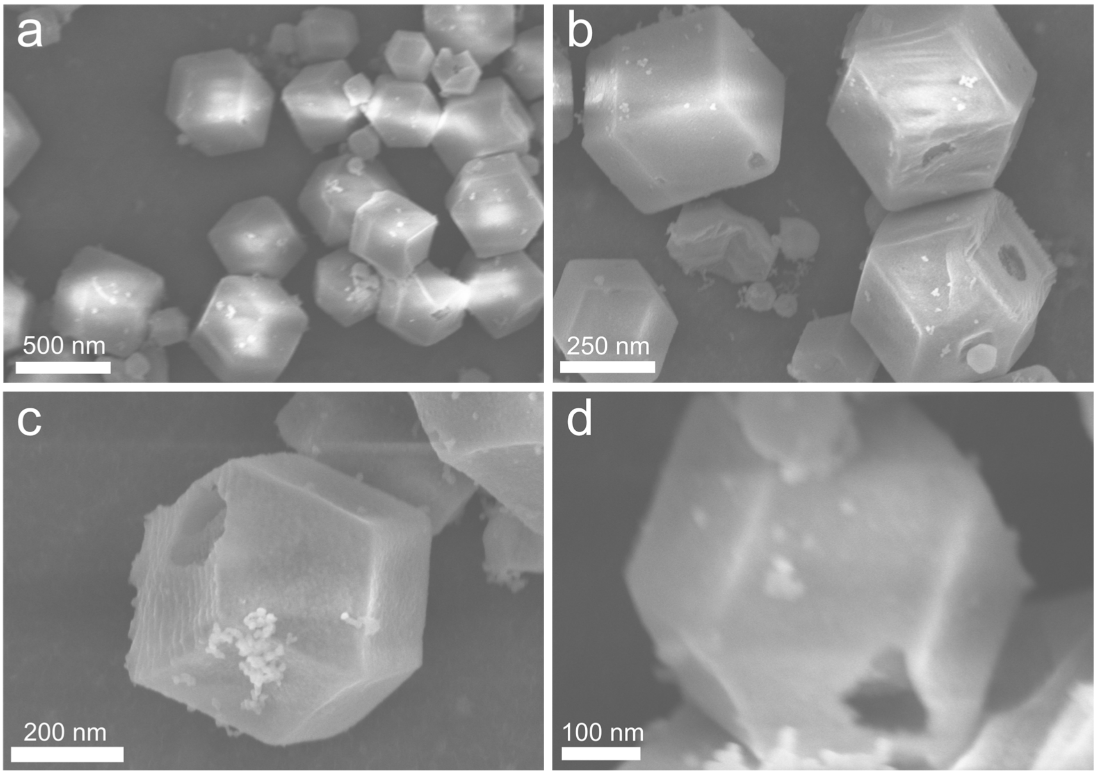

Figure S6. a-d) SEM images of FeCo-O-Se in different magnifications.

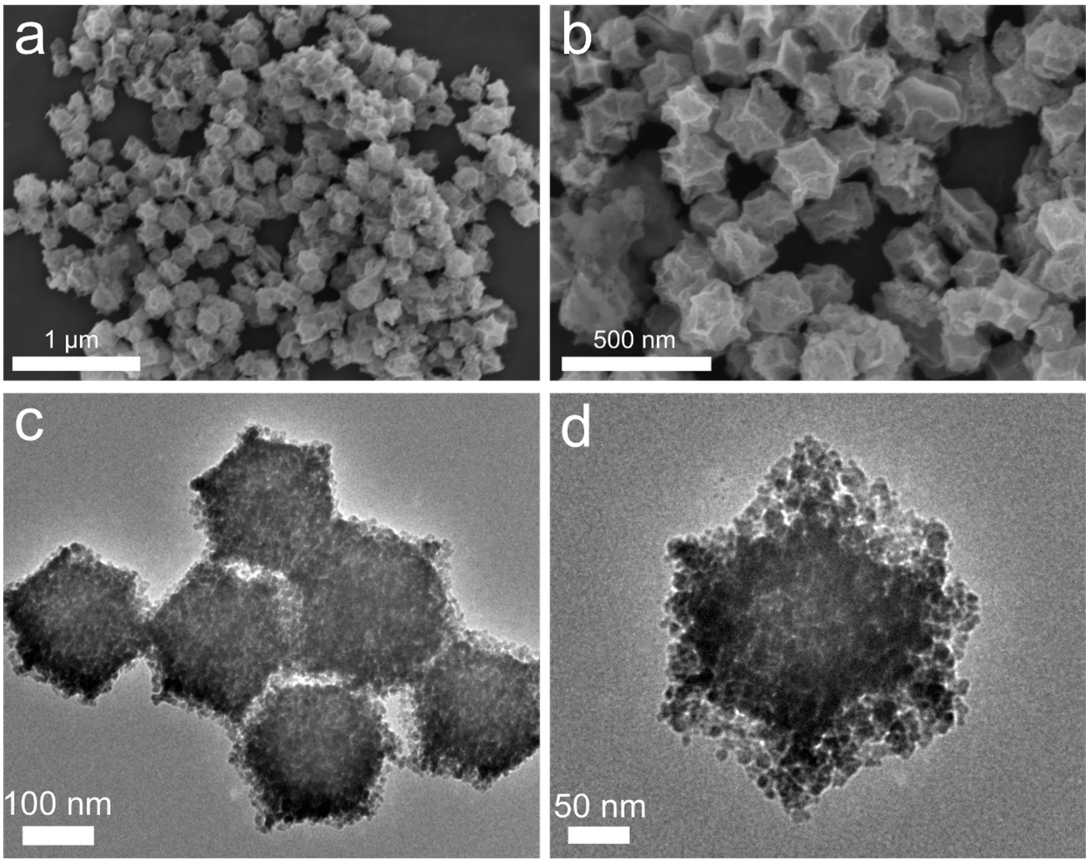

Figure S7. a-b) SEM and c-d) TEM images of Co_3O_4.

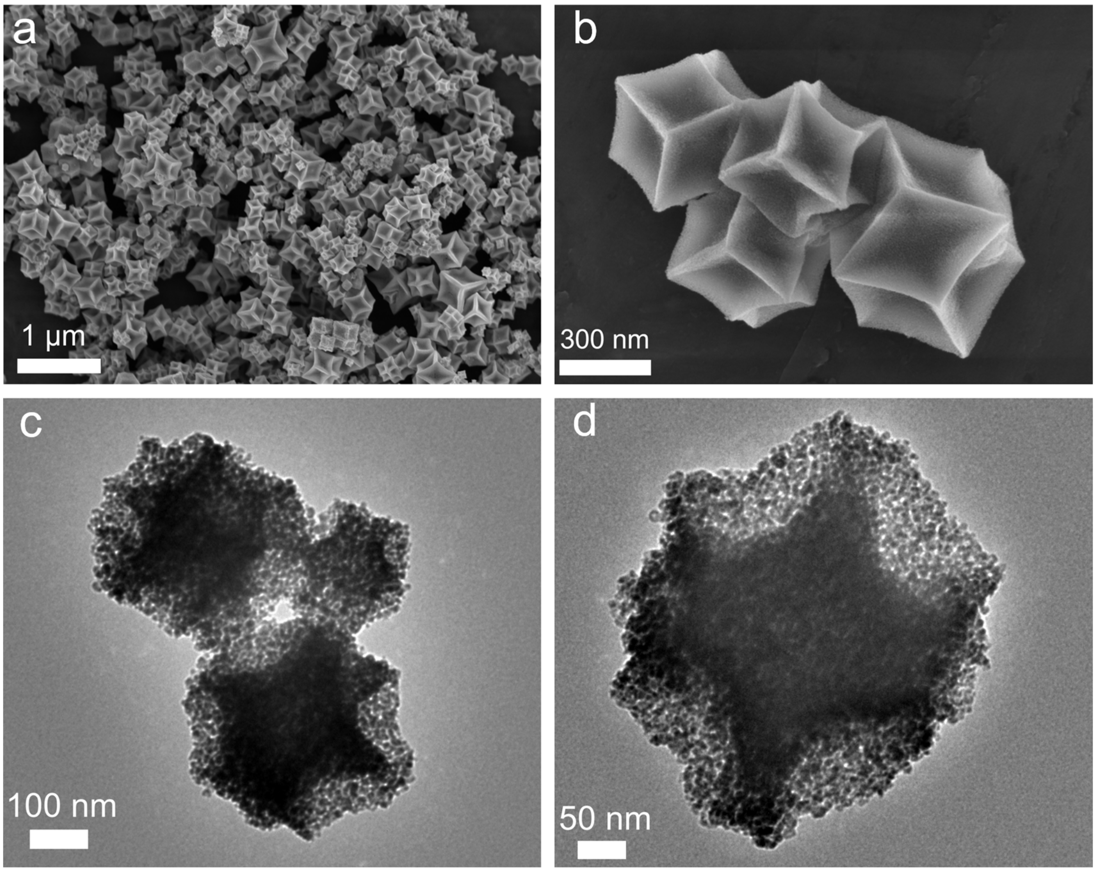

Figure S8. a-b) SEM and c-d) TEM images of $Fe_{0.1}Co_{2.9}O_4$.

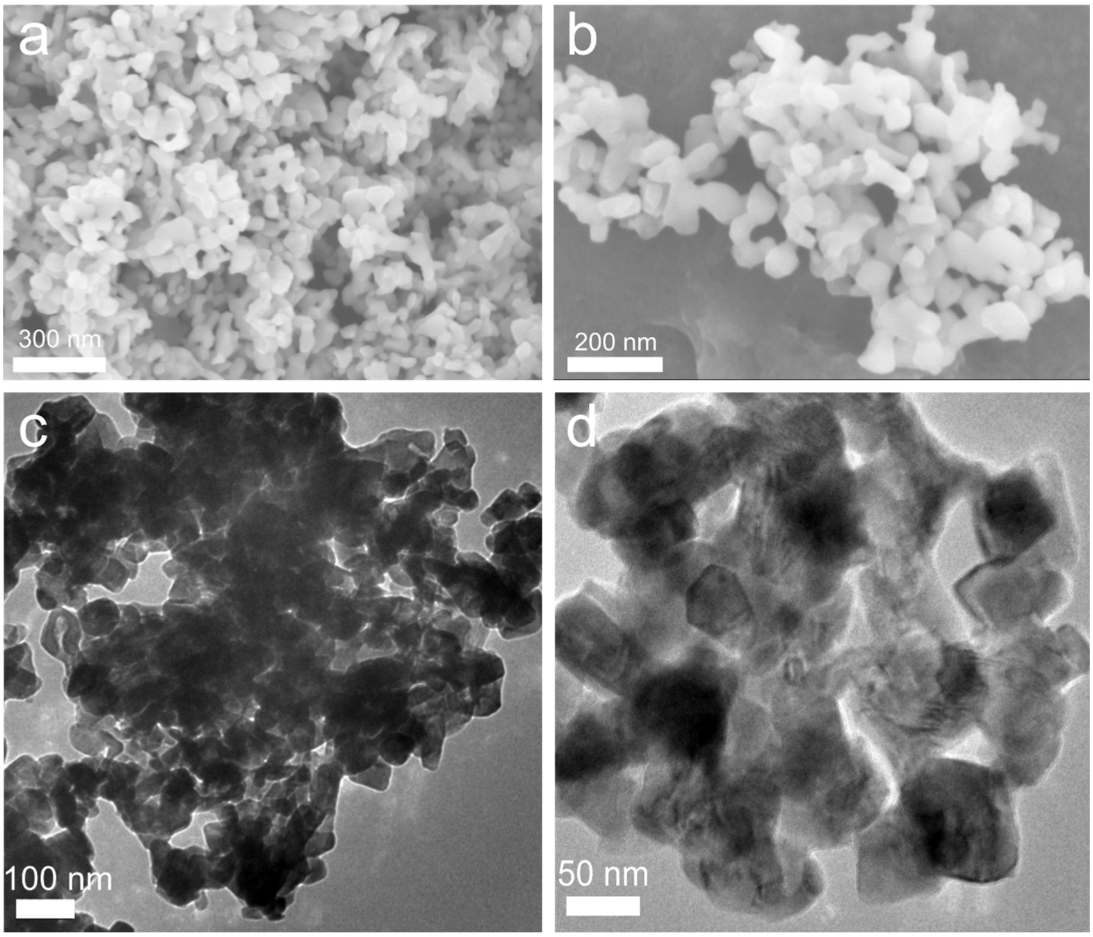

Figure S9. a-b) SEM and c-d) TEM images of Co_3O_4-Se.

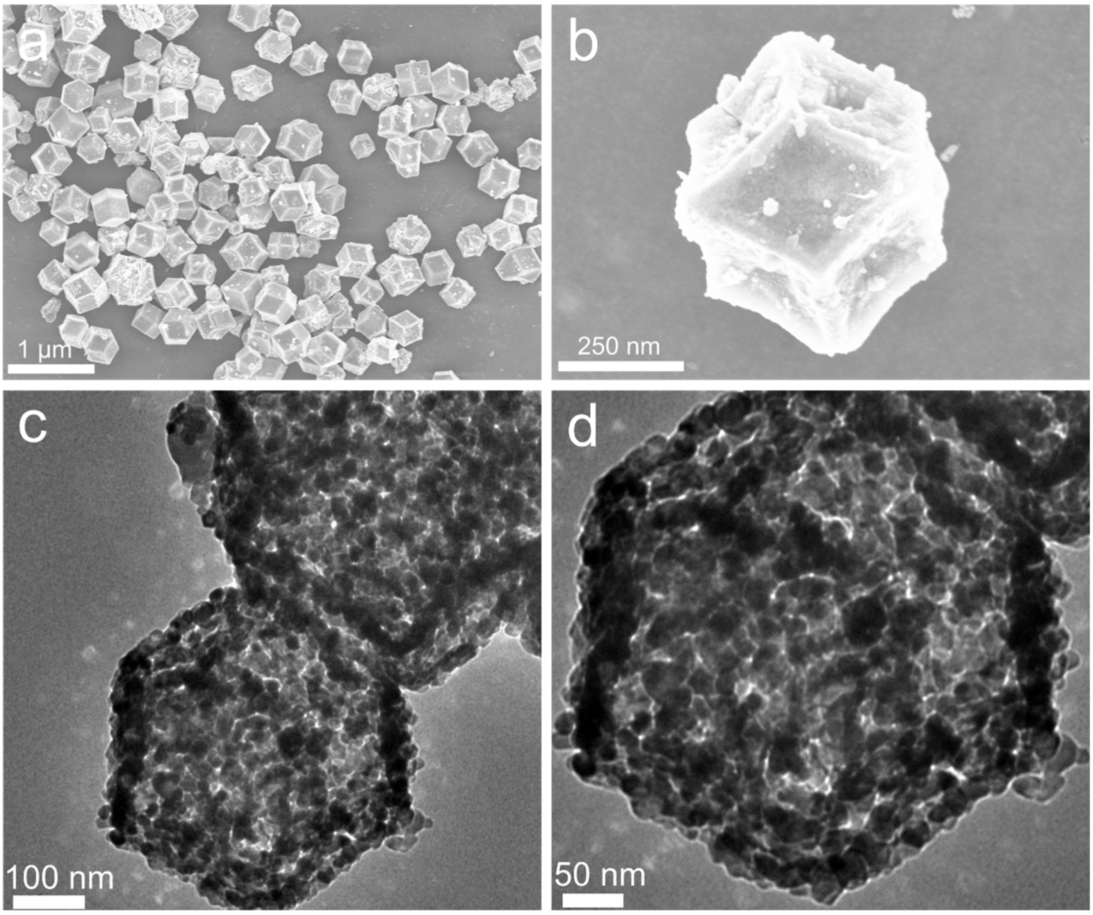

Figure S10. a-b) SEM and c-d) TEM images of $Fe_{0.1}Co_{2.9}O_4$-Se.

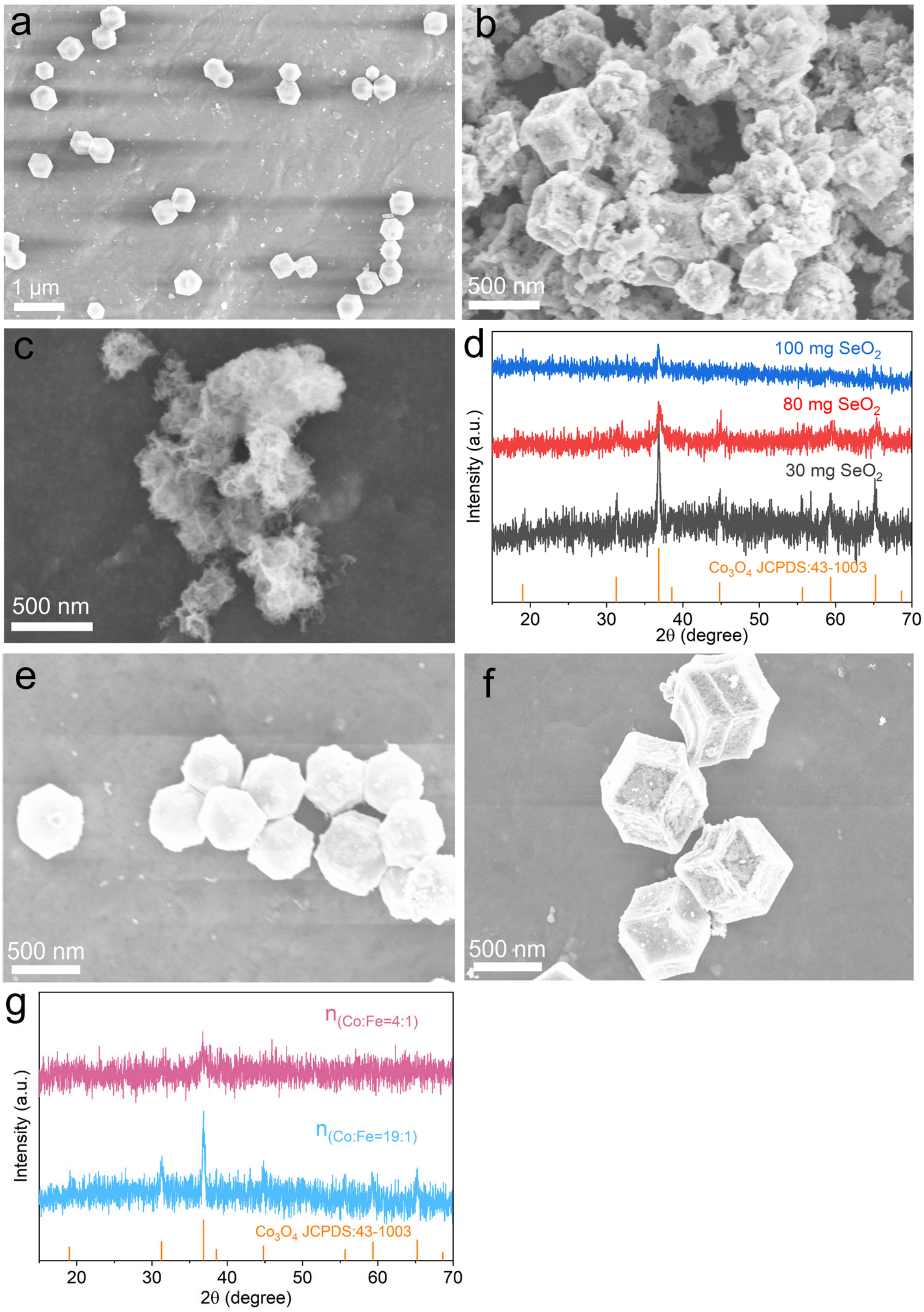

Figure S11. The synthesis procedures are the same as those of $Fe_{0.1}Co_{2.9}O_4$-Se, except using the different amounts of SeO_2 and Fe. SEM images of those with SeO_2 contents of a) 30 mg, b) 80 mg, c) 100 mg. d) XRD patterns of $Fe_{0.1}Co_{2.9}O_4$-Se synthesized with the above different SeO_2 contents. e) SEM images of $Fe_{0.1}Co_{2.9}O_4$-Se with different molar amounts (n) of Fe and Co added: e) n (Co:Fe = 19:1), f) n (Co:Fe = 4:1). g) XRD patterns of $Fe_{0.1}Co_{2.9}O_4$-Se synthesized with the above two different Co:Fe molar ratios.

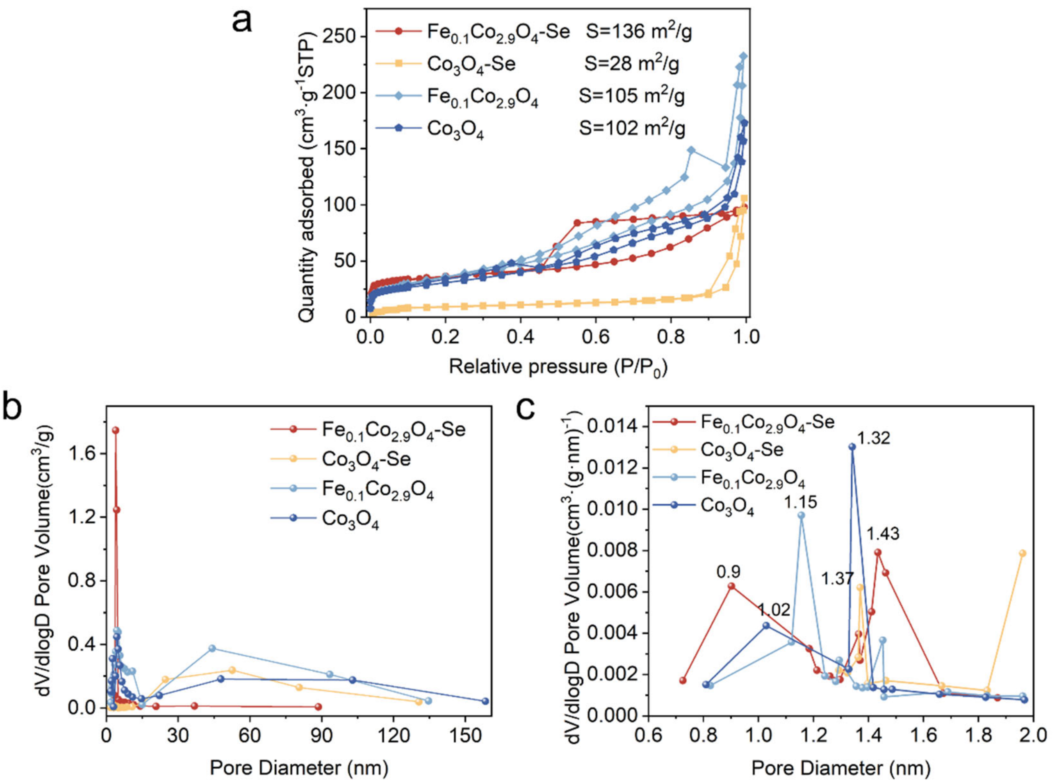

Figure S12. a) Specific surface areas, b) full pore size distributions, and c) micropore pore size distributions of the prepared samples.

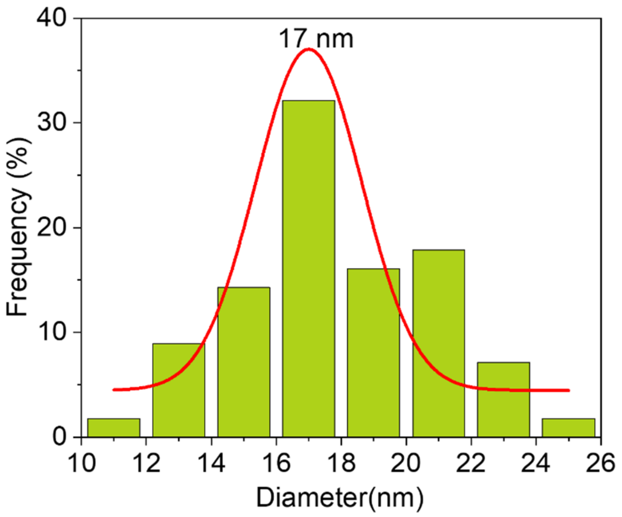

Figure S13. Size distribution histogram of $Fe_{0.1}Co_{2.9}O_4$-Se nanoparticles dispersed within the porous dodecahedral carbon.

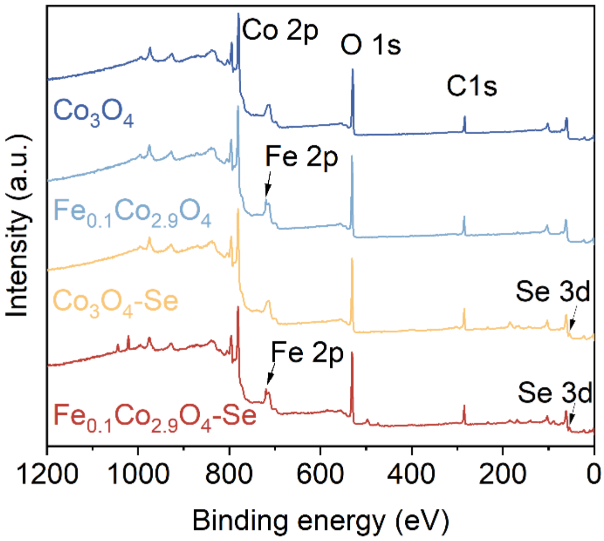

Figure S14. XPS survey spectra of the prepared samples.

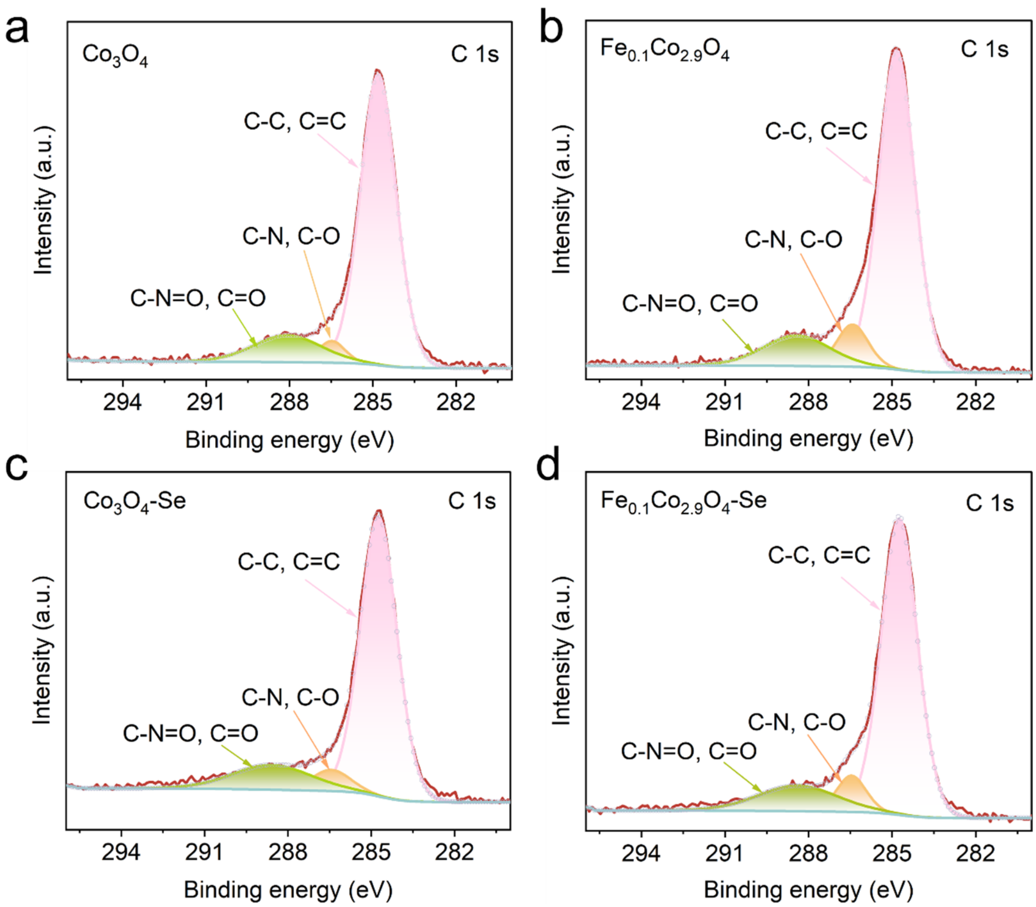

Figure S15. a-d) High-resolution XPS spectra of C1s for the prepared samples.

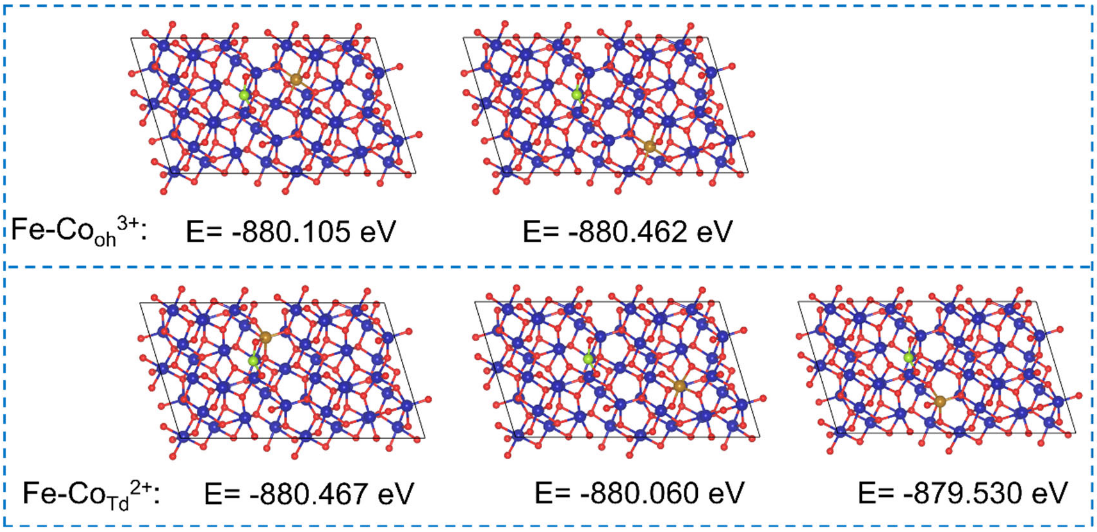

Figure S16. Configurations of Fe occupying the octahedral (Fe-Co_{Oh}^{3+}) and tetrahedral (Fe-Co_{Td}^{2+}) sites of Co_3O_4, and their corresponding free energies.

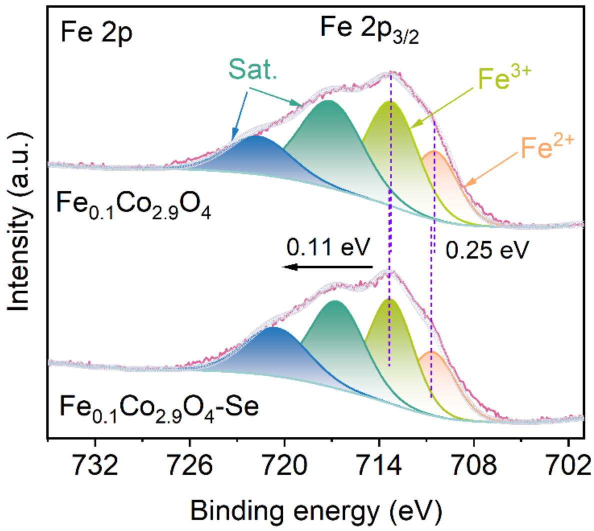

Figure S17. High-resolution XPS spectra of Fe 2p in $Fe_{0.1}Co_{2.9}O_4$-Se and $Fe_{0.1}Co_{2.9}O_4$.

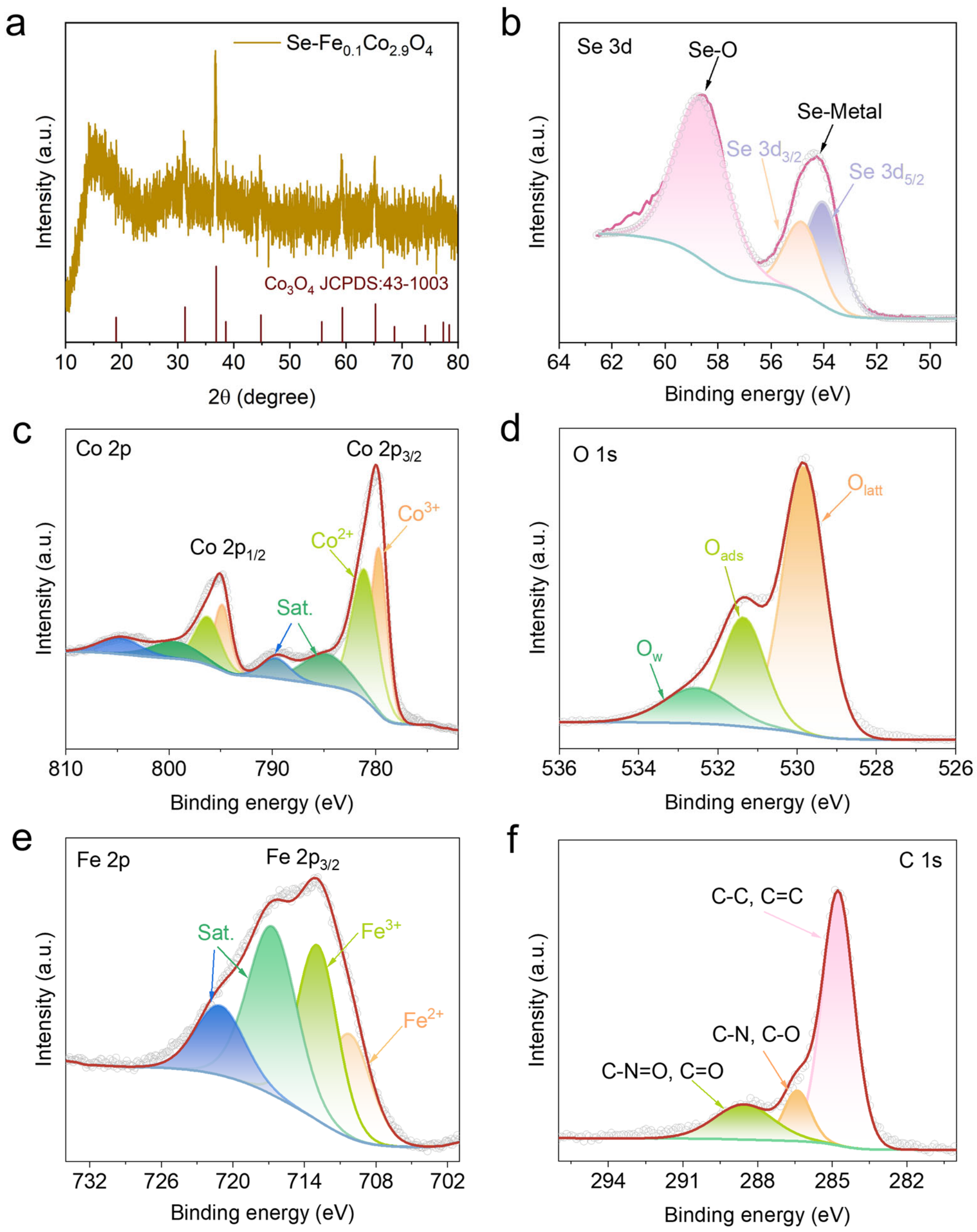

Figure S18. a) XRD pattern and b-f) high-resolution XPS spectra of Se 3d, Co 2p, O1s, Fe 2p and C1s in Se-$Fe_{0.1}Co_{2.9}O_4$.

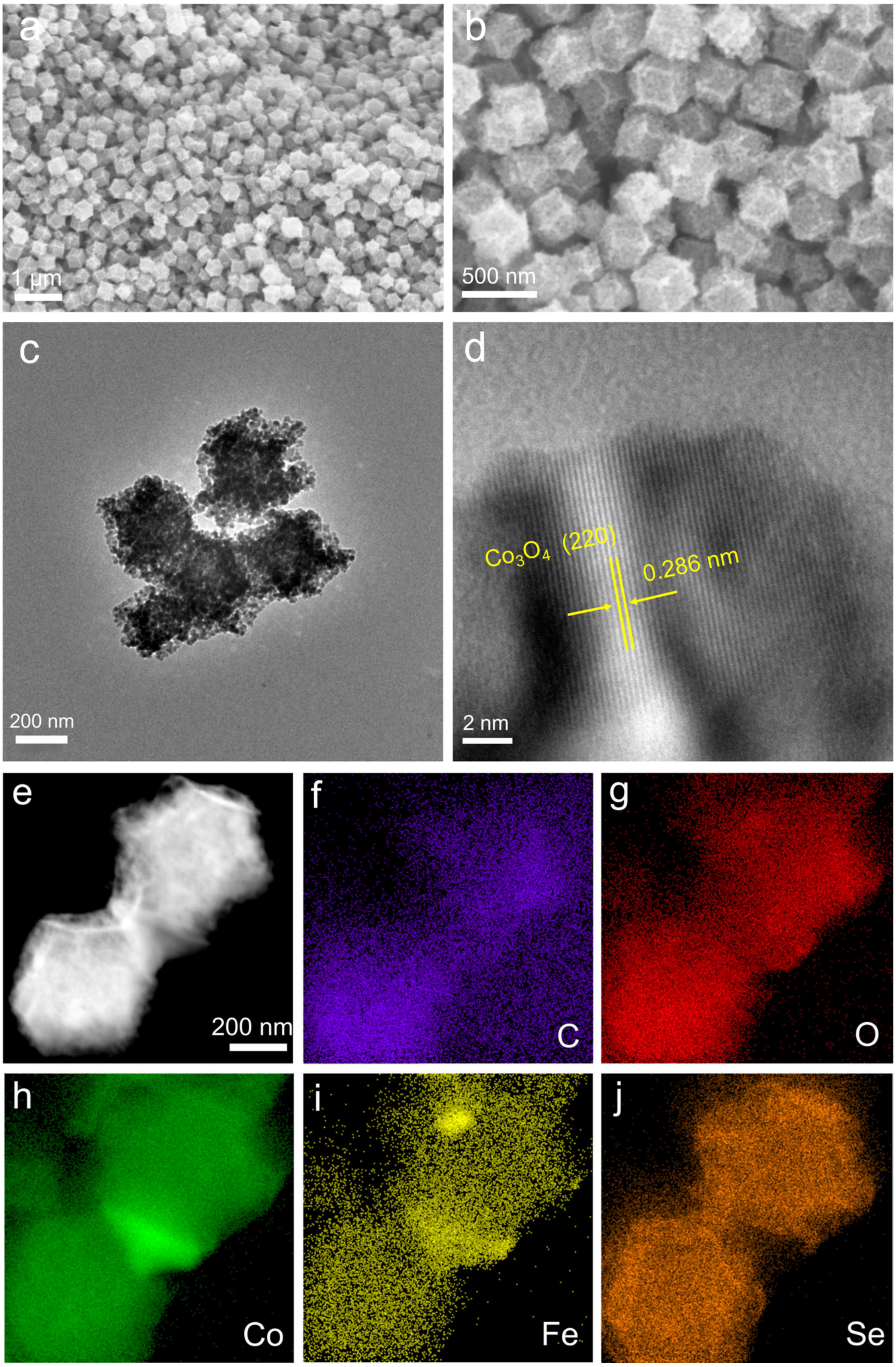

Figure S19. Morphology characterization of Se-$Fe_{0.1}Co_{2.9}O_4$. a-b) SEM images. c) TEM image. d) HRTEM image. e-j) The elemental mapping images.

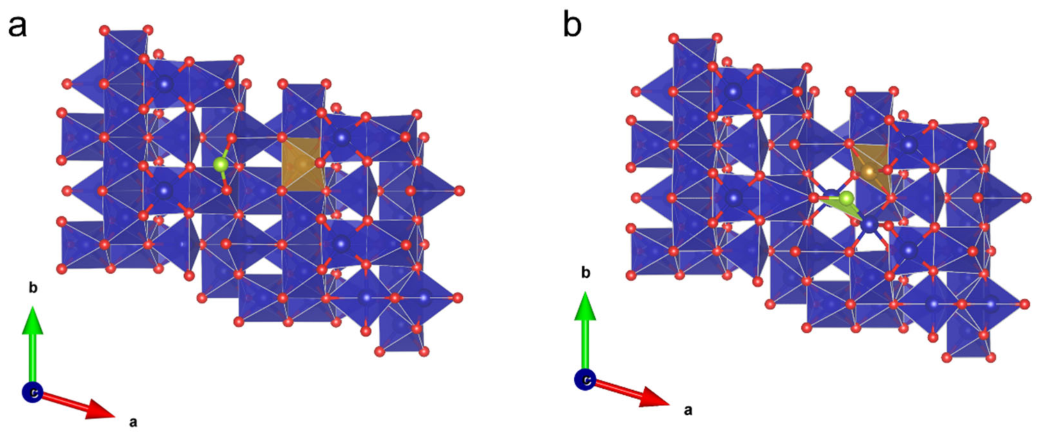

Figure S20. Top views of molecular structure diagrams. a) $Fe_{0.1}Co_{2.9}O_4$-Se. b) Se-$Fe_{0.1}Co_{2.9}O_4$.

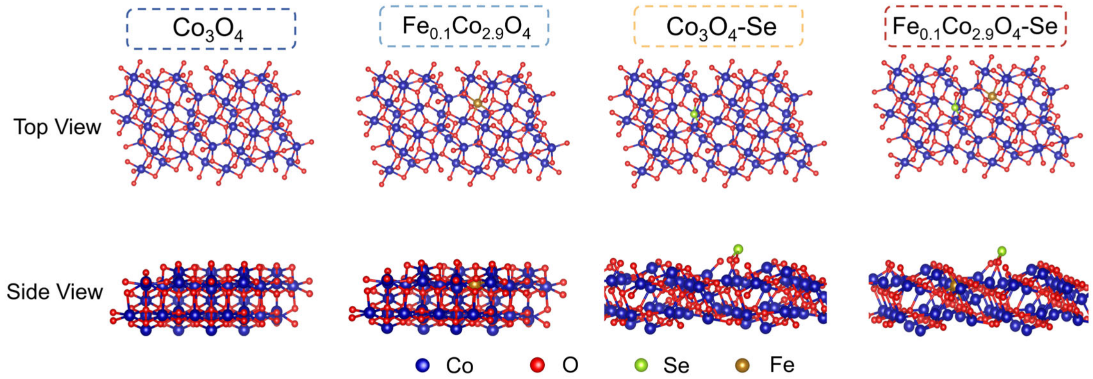

Figure S21. Top and Side views for the structures of the prepared samples.

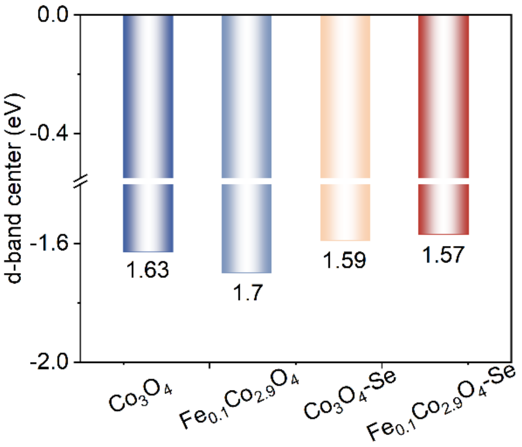

Figure S22. The d-band electrons parameters for the prepared samples.

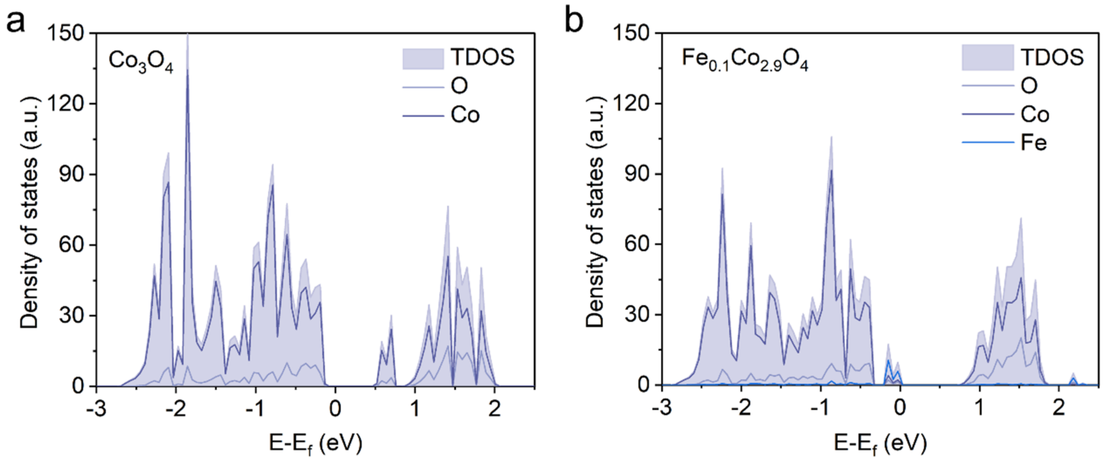

Figure S23. Density of states results of a) Co_3O_4, b) $Fe_{0.1}Co_{2.9}O_4$.

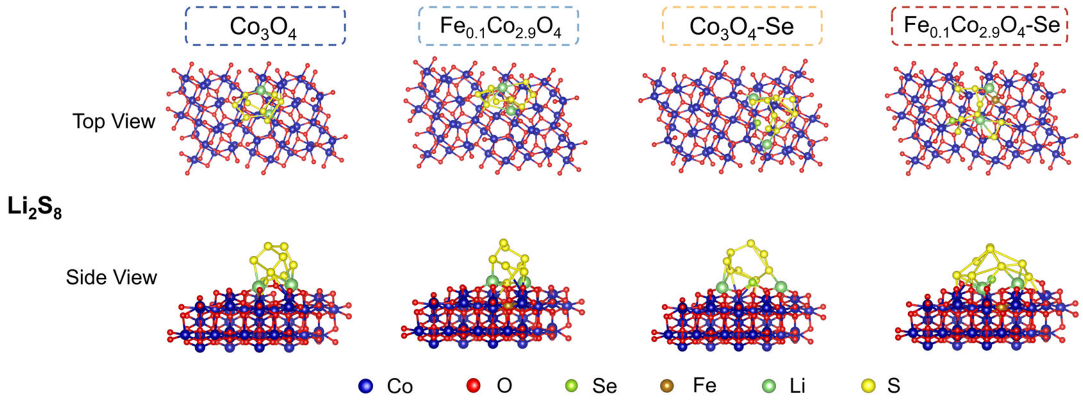

Figure S24. Top and side views for the Li_2S_8 adsorption configurations on the prepared samples.

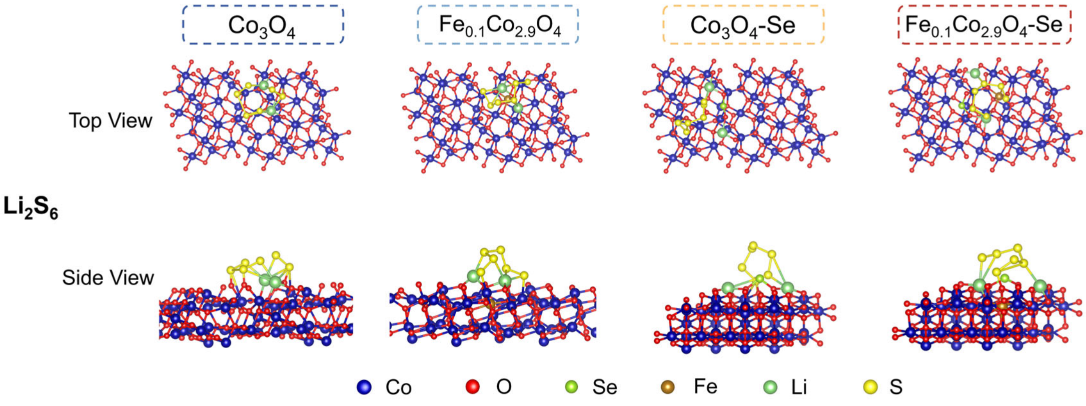

Figure S25. Top and side views for the Li_2S_6 adsorption configurations on the prepared samples.

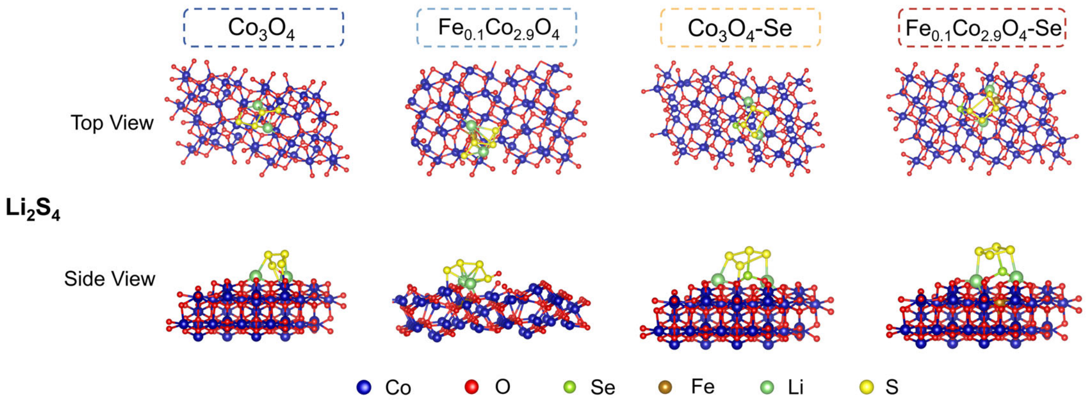

Figure S26. Top and side views for the Li_2S_4 adsorption configurations on the prepared samples.

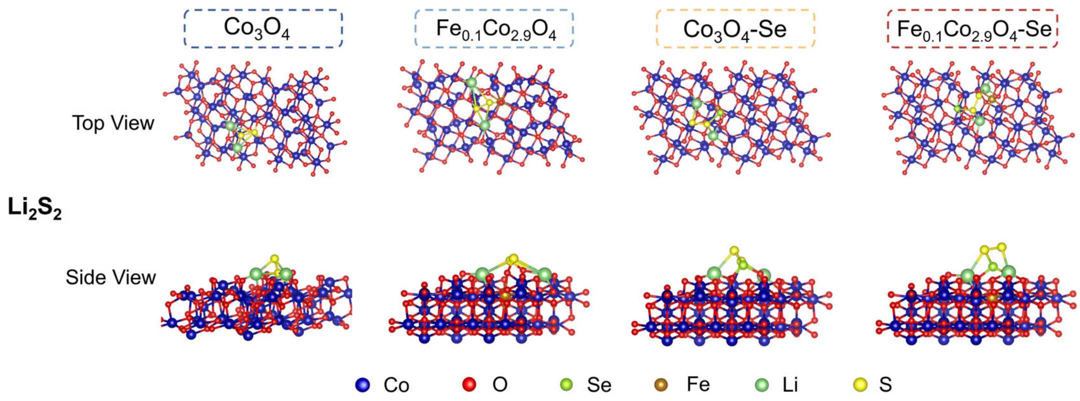

Figure S27. Top and side views for the Li_2S_2 adsorption configurations on the prepared samples.

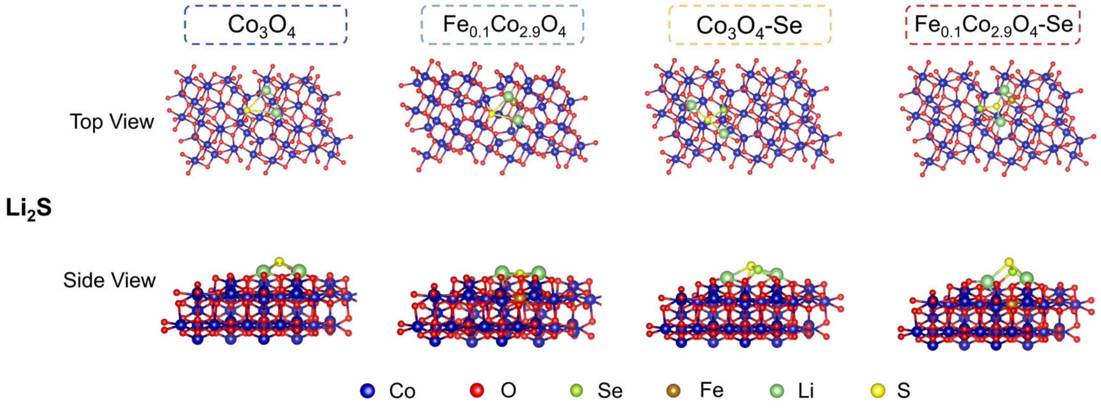

Figure S28. Top and side views for the Li_2S adsorption configurations on the prepared samples.

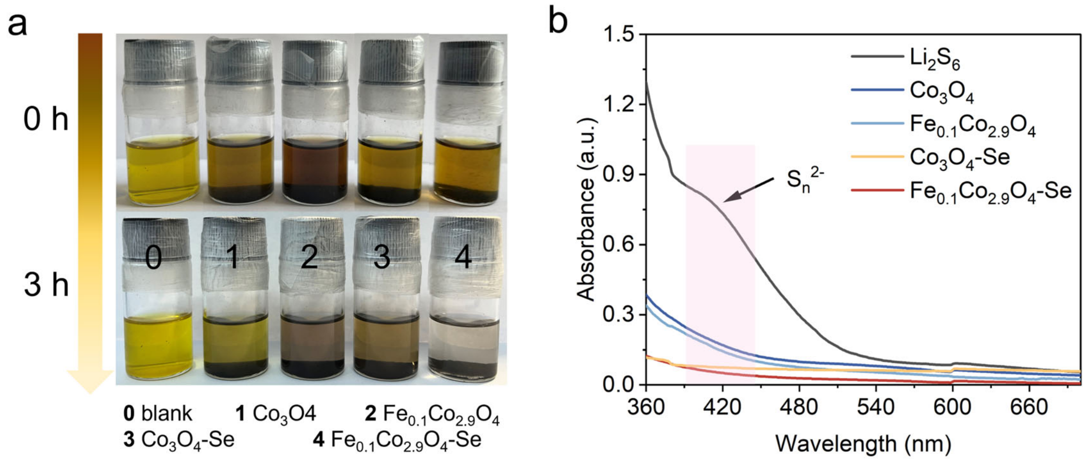

Figure S29. a) Digital images of the different samples after being kept in the Li_2S_6 solution for 2 h (the blank solution is 0.5 M Li_2S_6 solution). b) UV-vis absorption spectra of the absorbed Li_2S_6 solution.

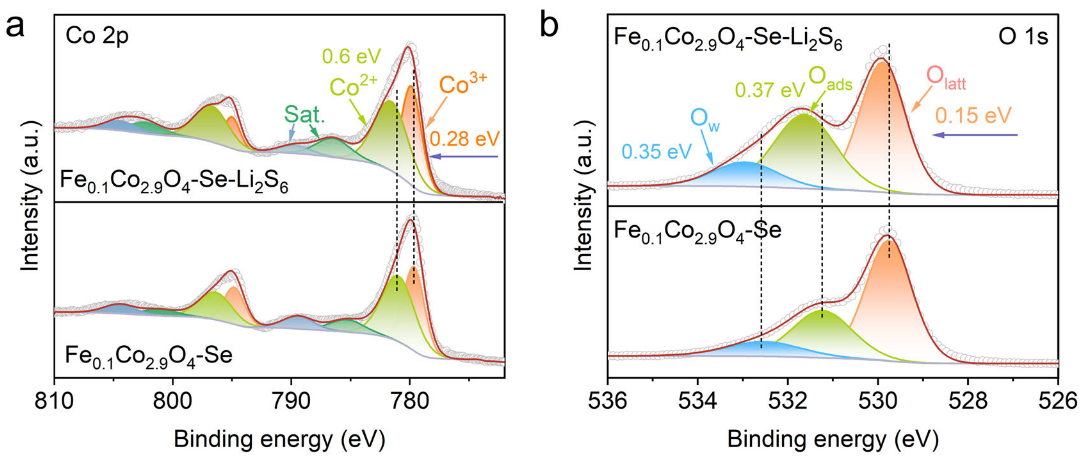

Figure S30. a) Co 2p and b) O 1s high-resolution spectra of $Fe_{0.1}Co_{2.9}O_4$-Se before and after absorbing Li_2S_6.

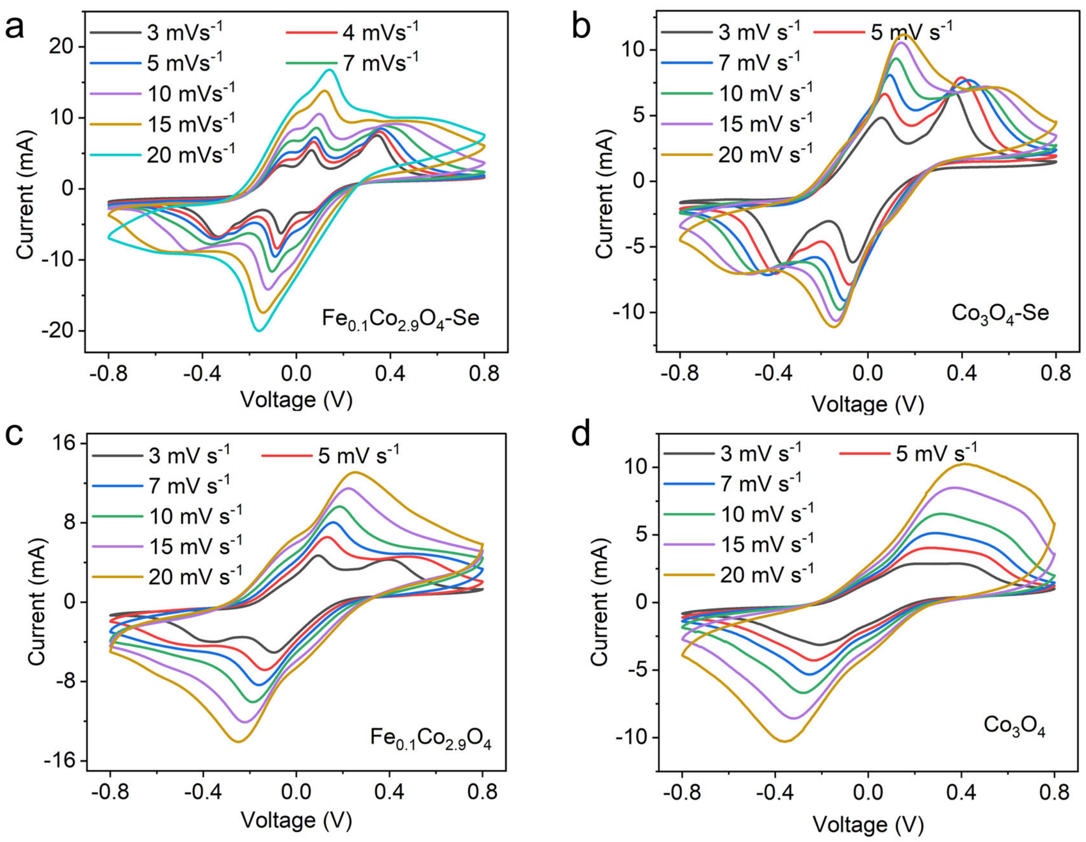

Figure S31. CV tests results of symmetric cells for $Fe_{0.1}Co_{2.9}O_4$-Se, Co_3O_4-Se, $Fe_{0.1}Co_{2.9}O_4$, and Co_3O_4.

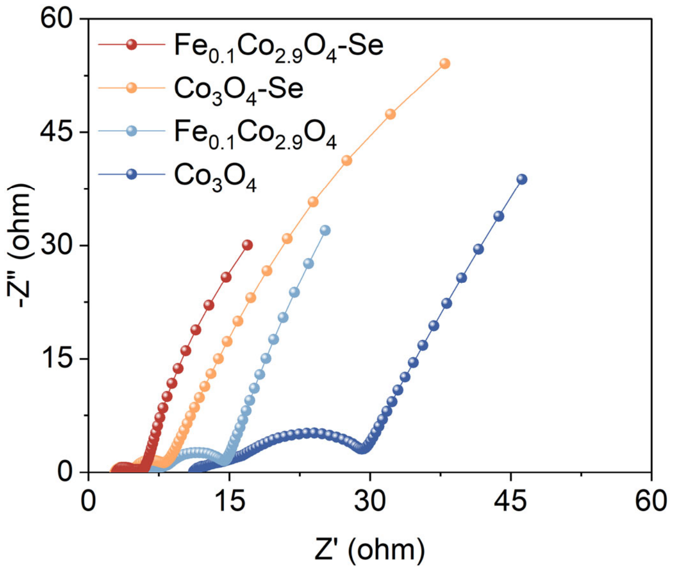

Figure S32. Nyquist plots of symmetric cells.

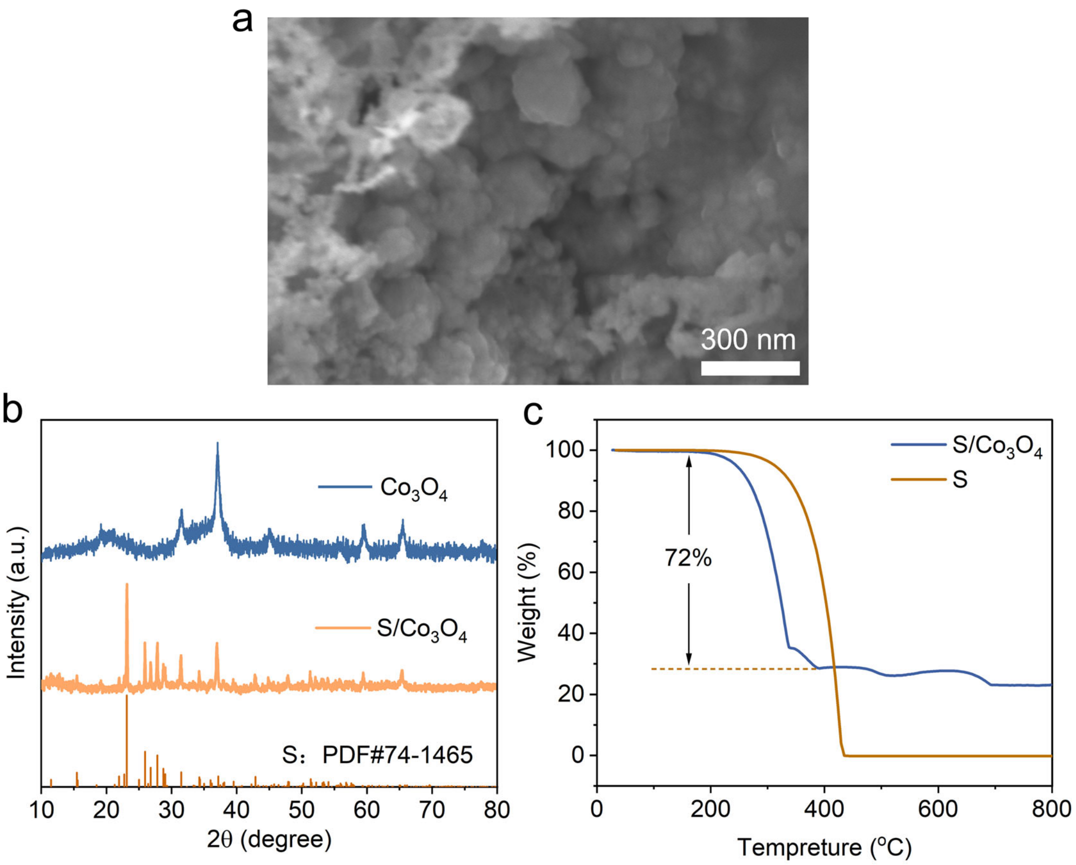

Figure S33. Characterization results of S/Co_3O_4. a) SEM image, b) XRD pattern, and c) TGA curve.

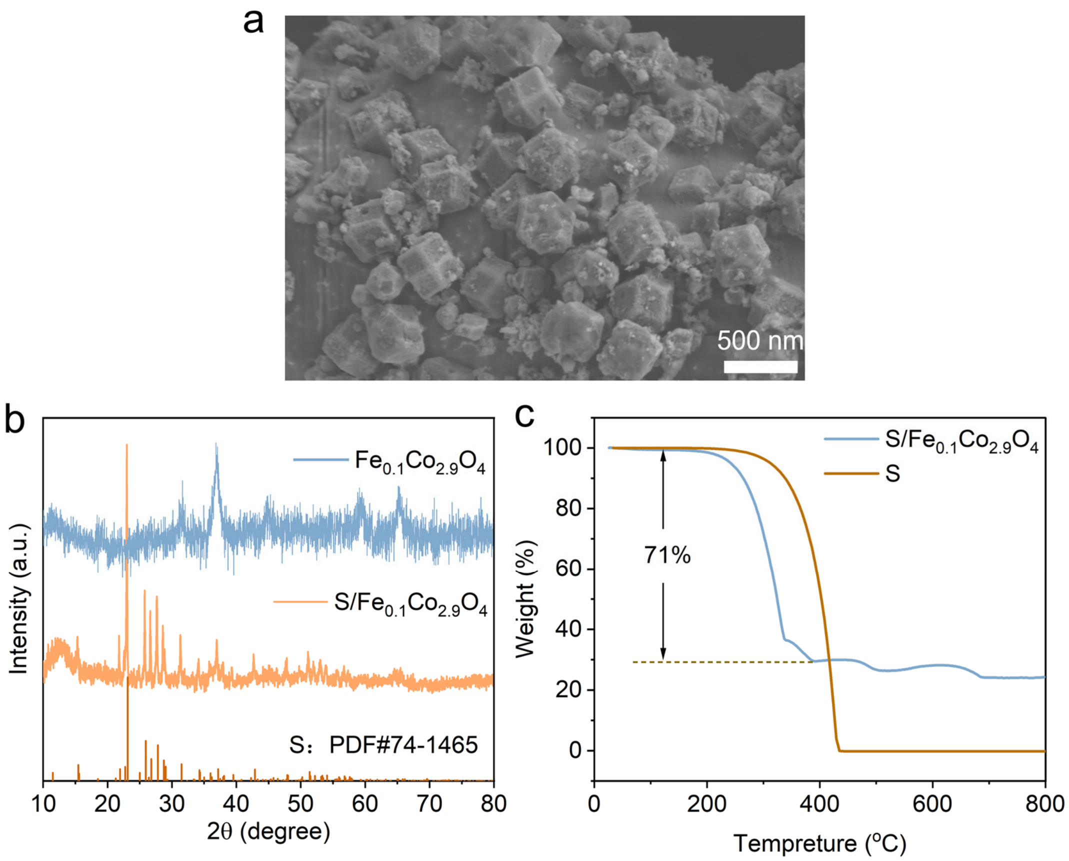

Figure S34. Characterization results of S/$Fe_{0.1}Co_{2.9}O_4$. a) SEM image, b) XRD pattern, and c) TGA curve.

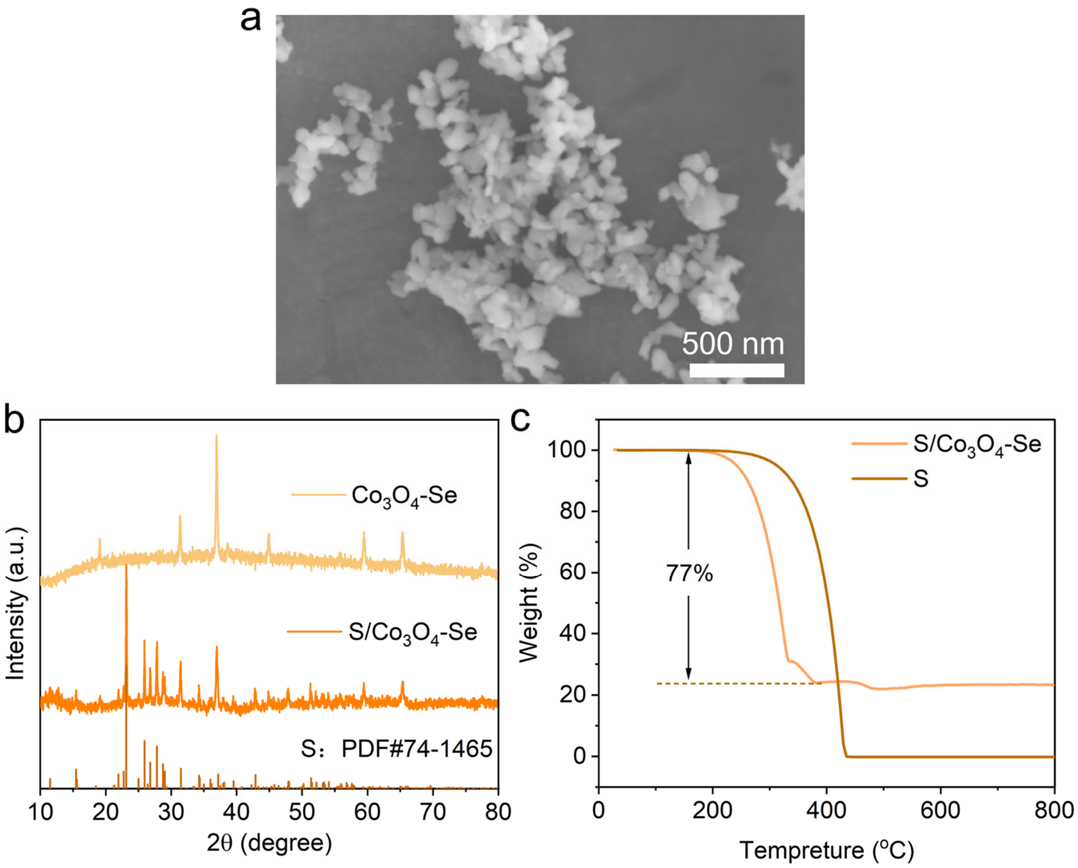

Figure S35. Characterization results of S/Co_3O_4-Se. a) SEM image, b) XRD pattern, and c) TGA curve.

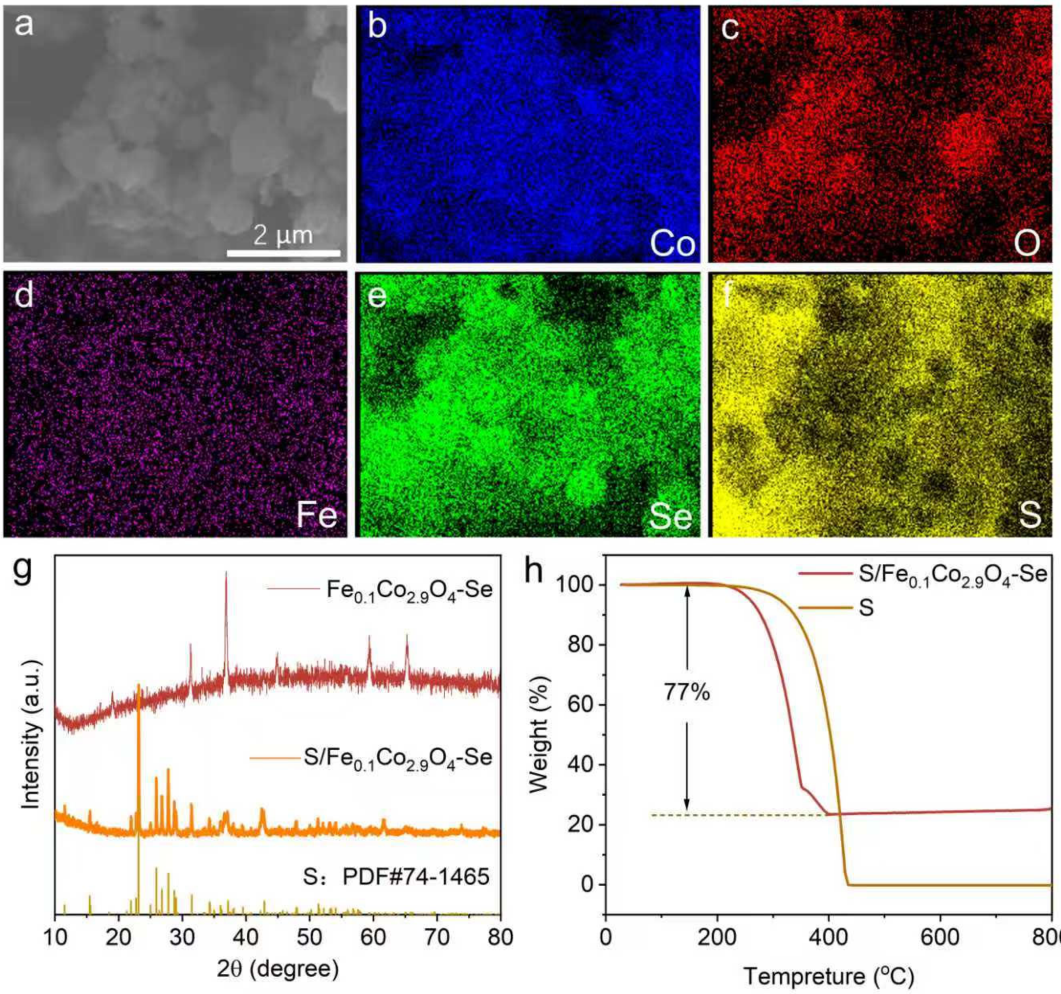

Figure S36. Characterization results of S/$Fe_{0.1}Co_{2.9}O_4$-Se. a) SEM image, b-f) EDS mapping, g) XRD pattern, and h) TGA curve.

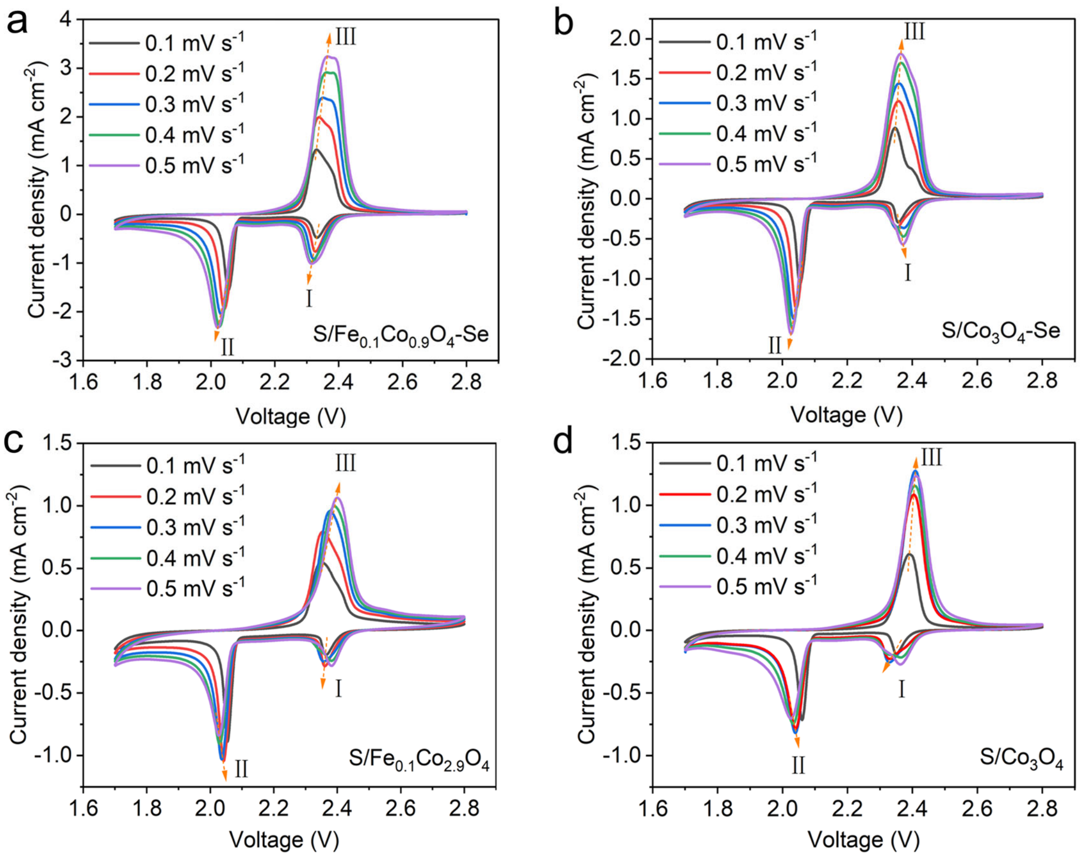

Figure S37. CV curves of LSBs paired with different cathodes. a) S/$Fe_{0.1}Co_{2.9}O_4$-Se, b) S/Co_3O_4-Se, c) S/$Fe_{0.1}Co_{2.9}O_4$ and d) S/Co_3O_4 in the voltage ranges of 1.7-2.8 V (vs. Li/Li^+) at different scan rates from 0.1 to 0.5 mV s^{-1}.

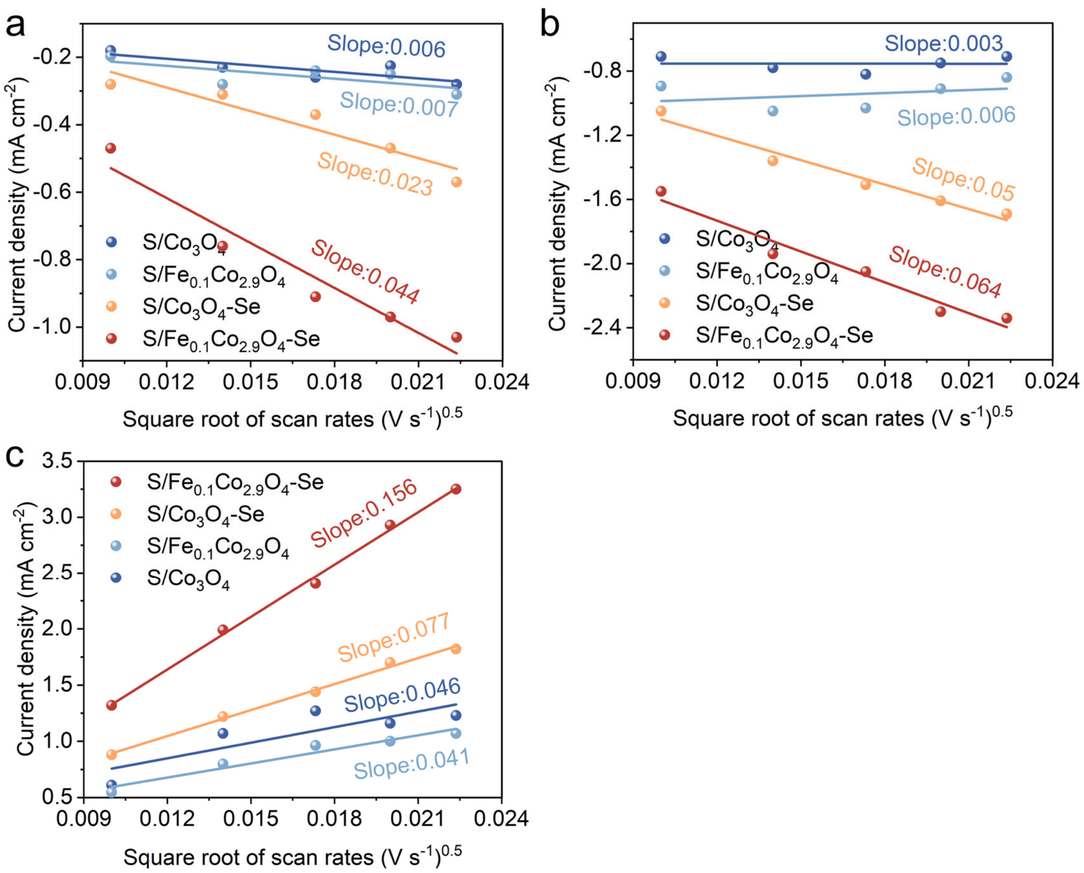

Figure S38. Plots of CV peak currents. a) the first cathodic reduction process (peak I: $S_8 \rightarrow Li_2S_x$), b) the second cathodic reduction process (peak II: $Li_2S_x \rightarrow Li_2S_2/Li_2S$), and c) the anodic oxidation process (peak III: $Li_2S_2/Li_2S \rightarrow S_8$) vs. square root of the scan rates.

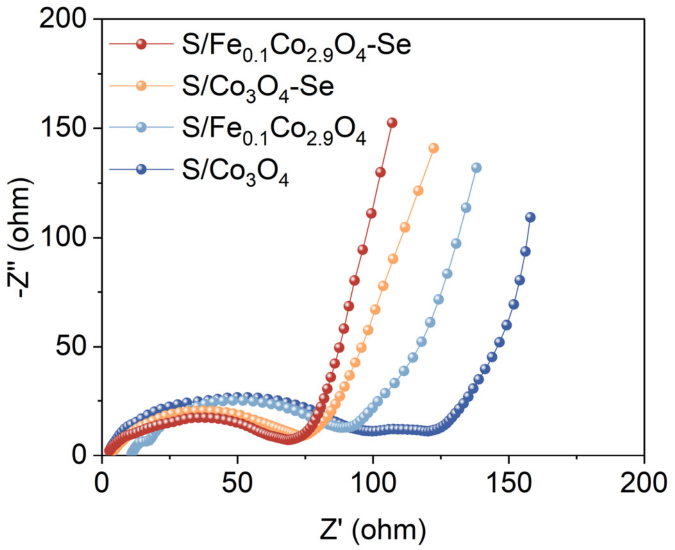

Figure S39. EIS spectra of the prepared samples.

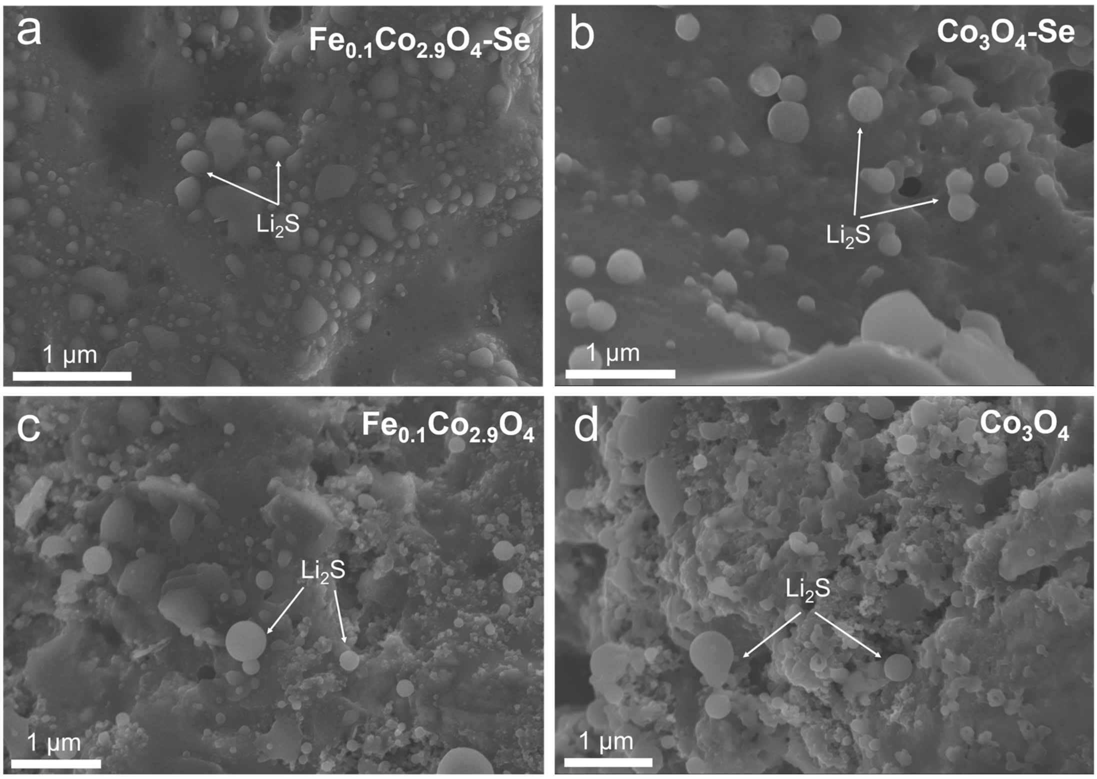

Figure S40. SEM images of Li_2S deposited on different catalyst surfaces. a) $Fe_{0.1}Co_{2.9}O_4$-Se, b) Co_3O_4-Se, c) $Fe_{0.1}Co_{2.9}O_4$, and d) Co_3O_4.

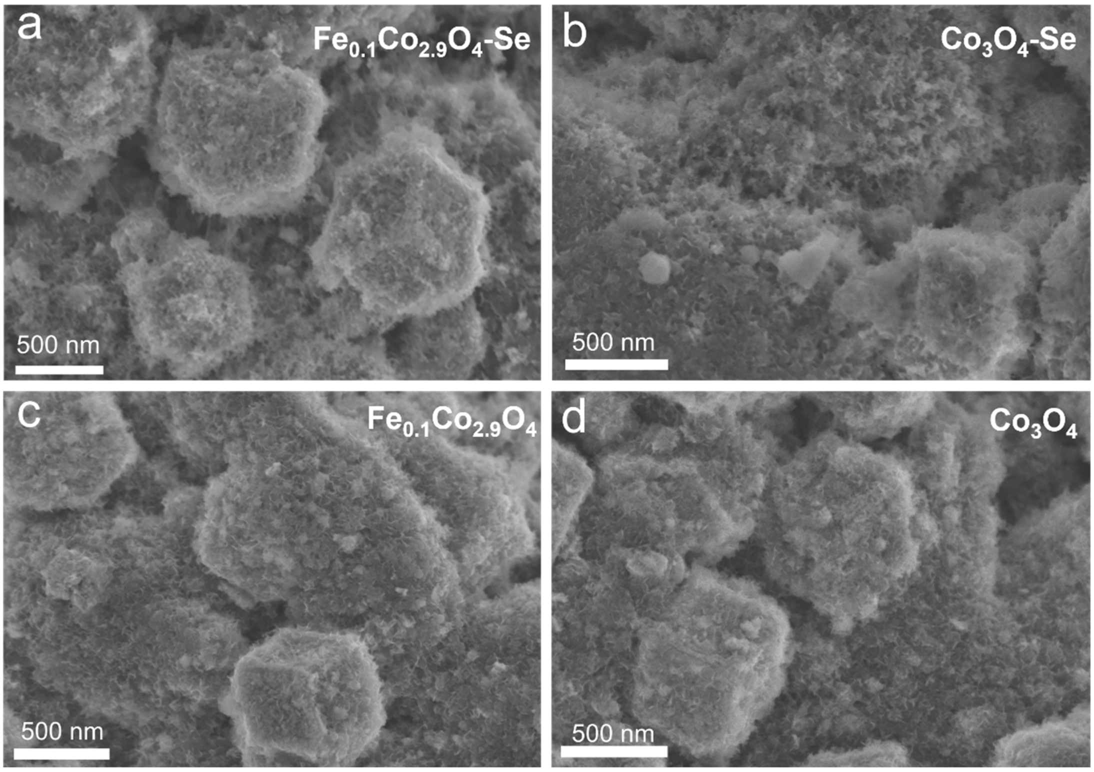

Figure S41. SEM images of Li_2S dissolution on different catalyst surfaces. a) $Fe_{0.1}Co_{2.9}O_4$-Se, b) Co_3O_4-Se, c) $Fe_{0.1}Co_{2.9}O_4$, and d) Co_3O_4.

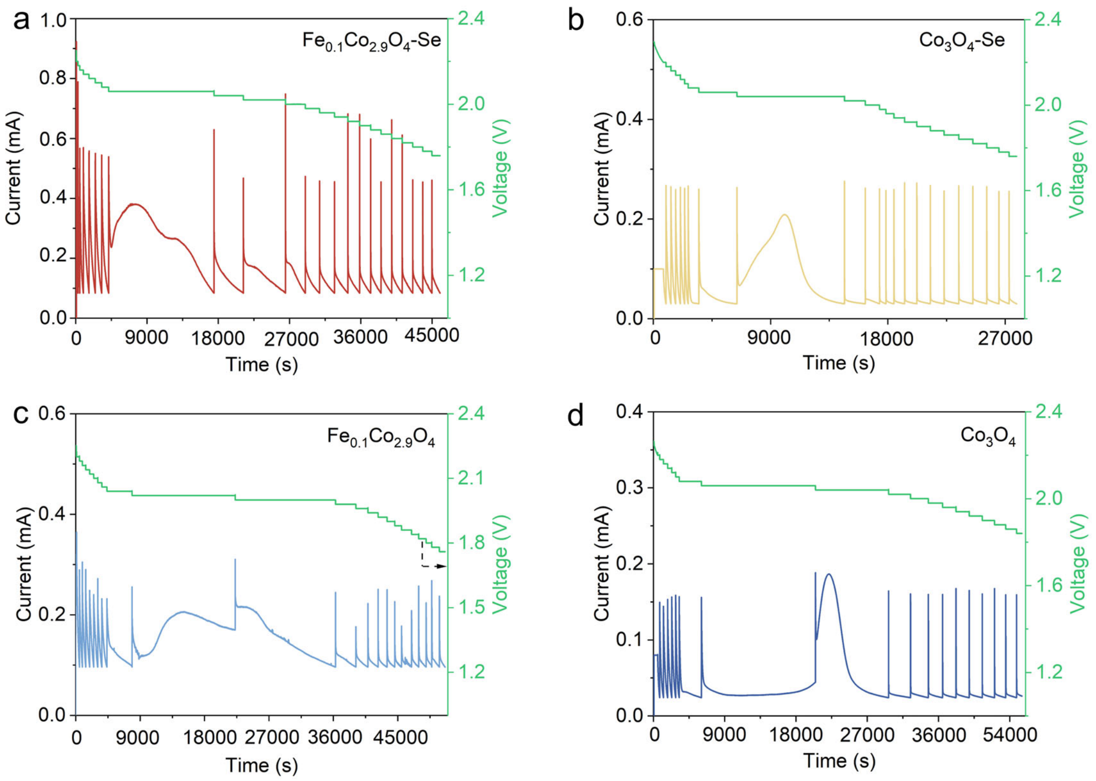

Figure S42. PITT profiles of the Li_2S_8 cells with different catalytic materials, showing the kinetic reaction process of S_8 discharge to Li_2S.

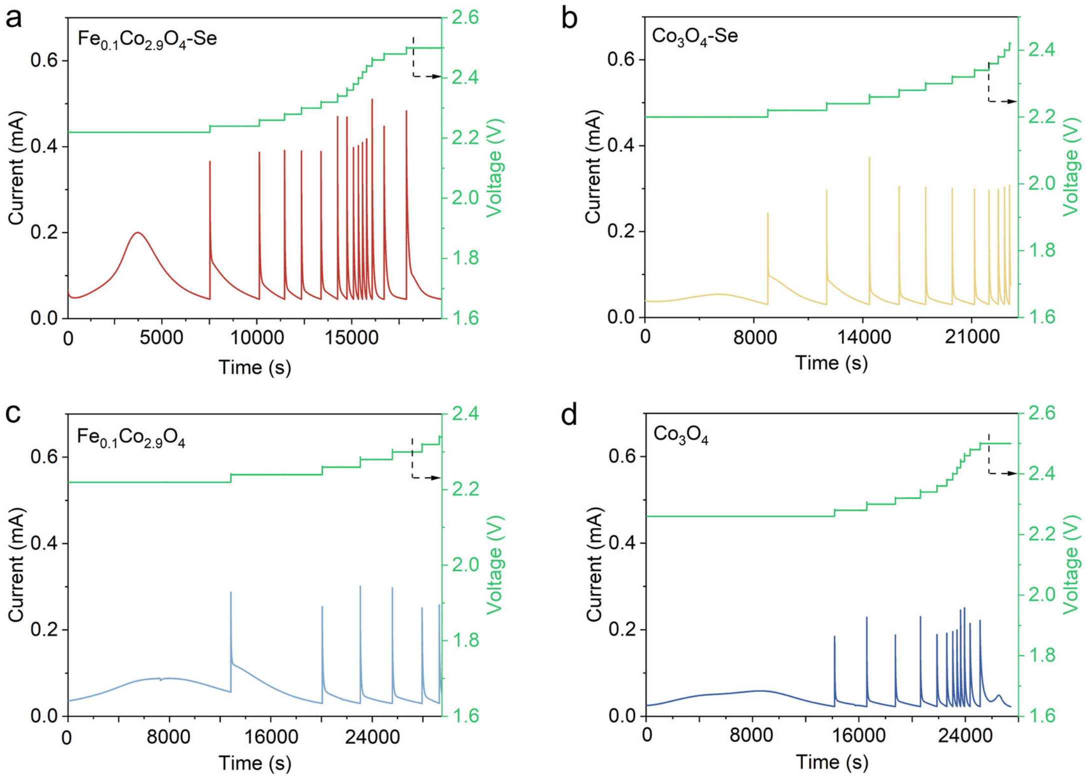

Figure S43. PITT profiles of the Li_2S_8 cells with different catalytic materials, showing the kinetic reaction process of Li_2S charge to S_8.

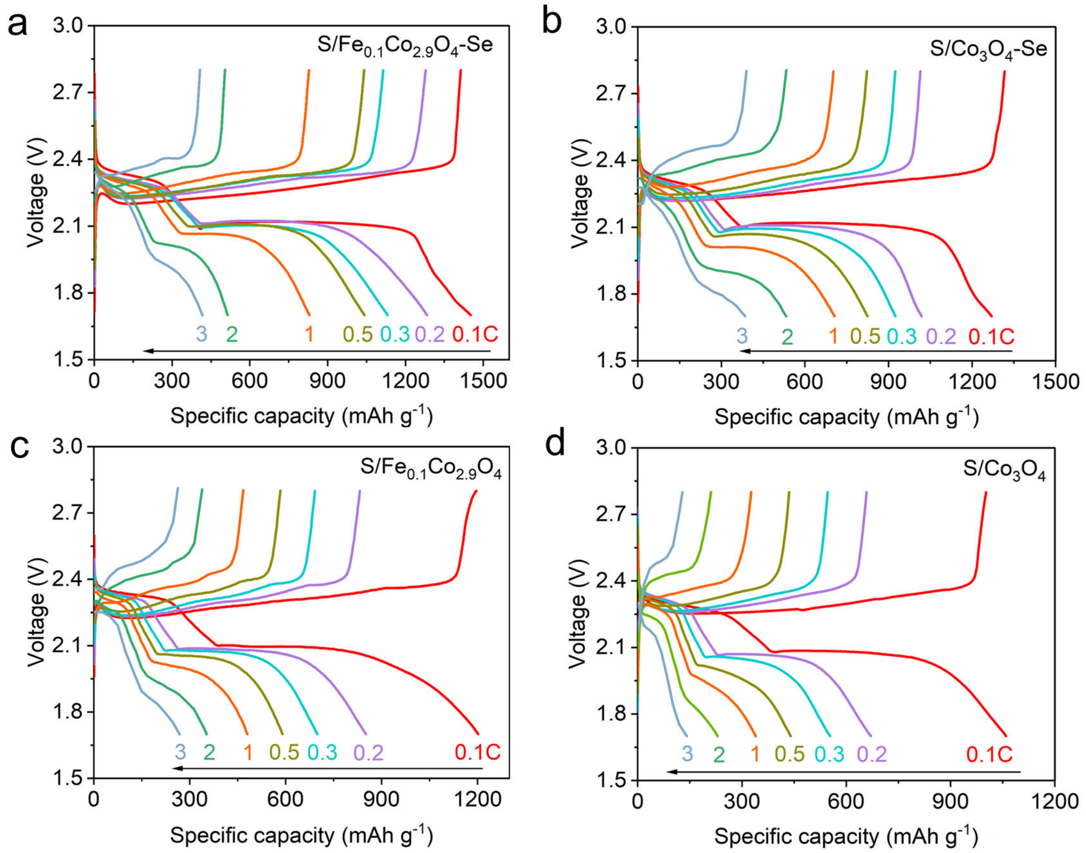

Figure S44. Galvanostatic charge-discharge profiles of LSBs for the prepared catalysts at different rates.

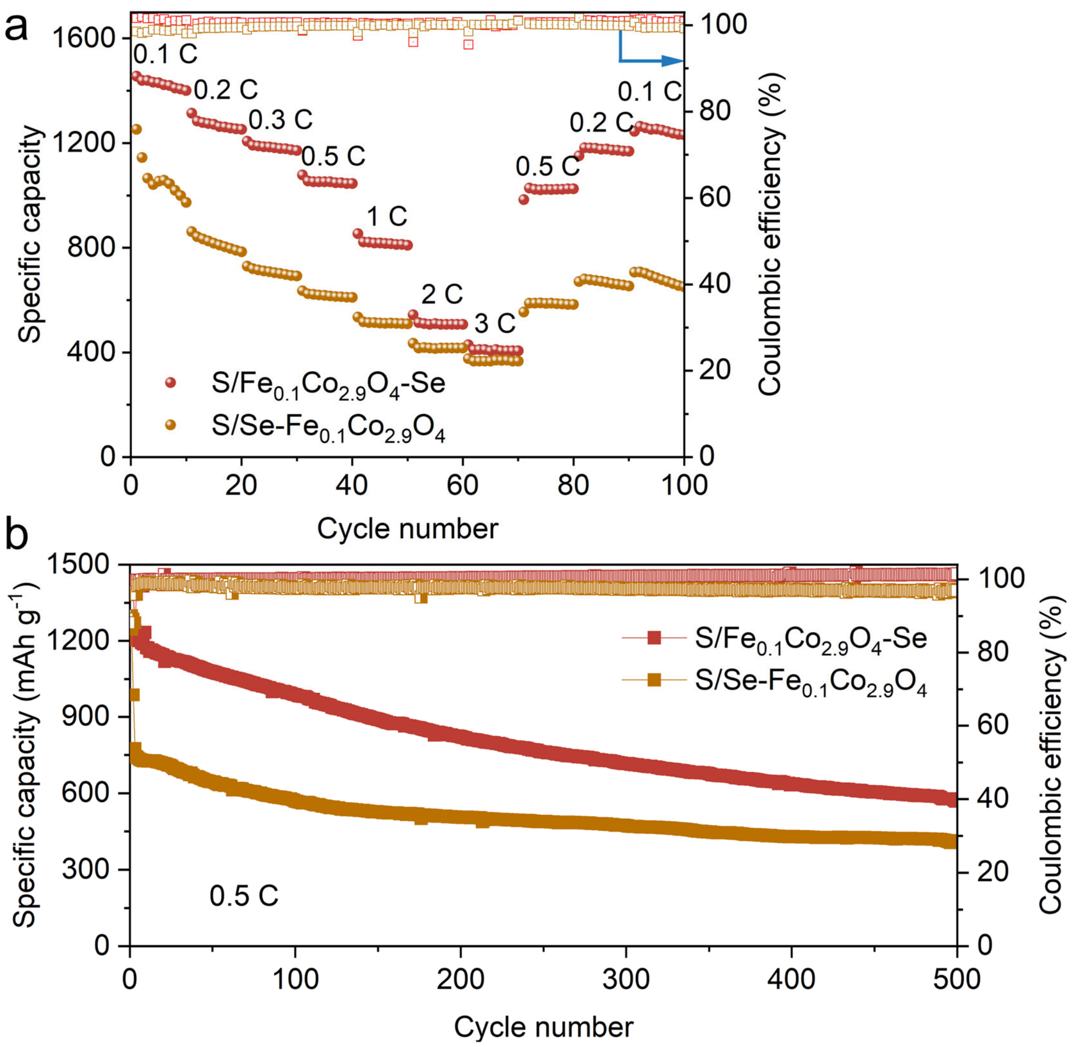

Figure S45. a) Rate performance and b) long cycle performance at 0.5 C of the $S/Fe_{0.1}Co_{2.9}O_4$-Se and S/Se-$Fe_{0.1}Co_{2.9}O_4$ electrodes.

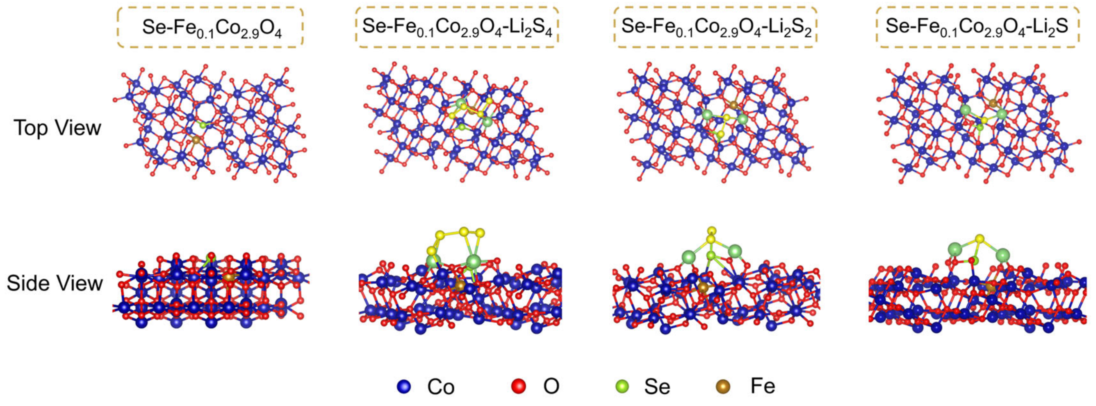

Figure S46. Top and side views for the adsorption configurations of Li_2S_4, Li_2S_2, and Li_2S on the Se-$Fe_{0.1}Co_{2.9}O_4$, respectively.

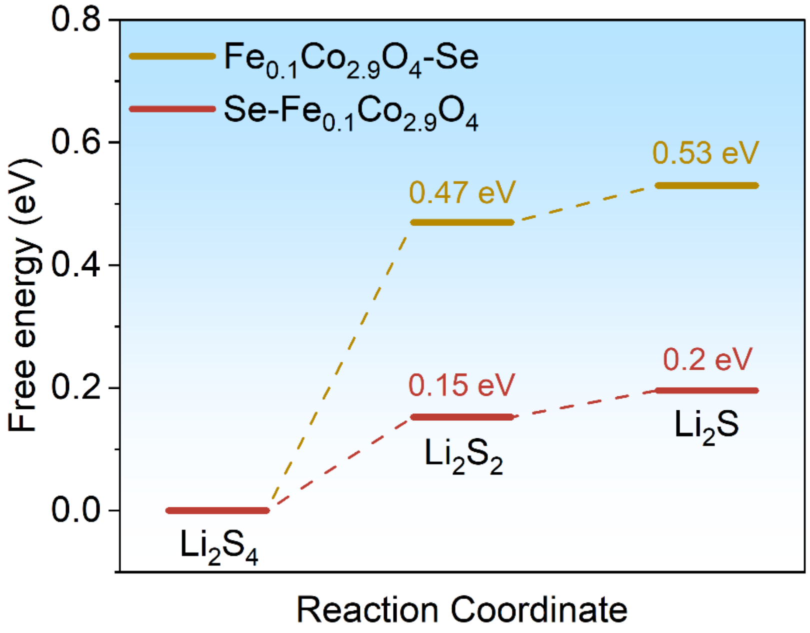

Figure S47. Gibbs free energy values for the reduction of Li_2S_4 to Li_2S on the surfaces of $Fe_{0.1}Co_{2.9}O_4$-Se and Se-$Fe_{0.1}Co_{2.9}O_4$, respectively.

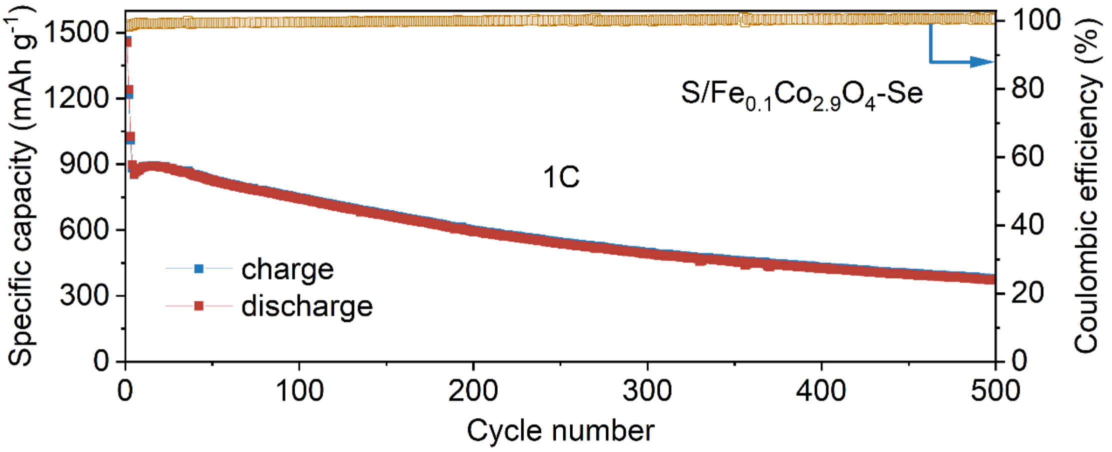

Figure S48. Charge and discharge curves of the $S/Fe_{0.1}Co_{2.9}O_4$-Se-based Li-S battery at 1 C.

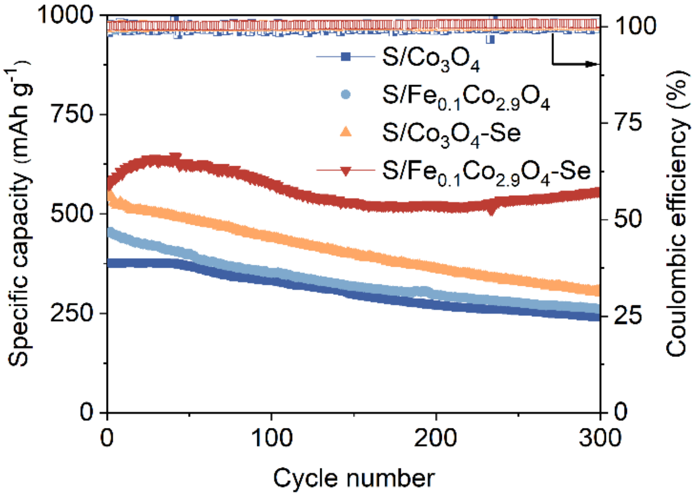

Figure S49. Cycling stability of all electrodes at 2 C.

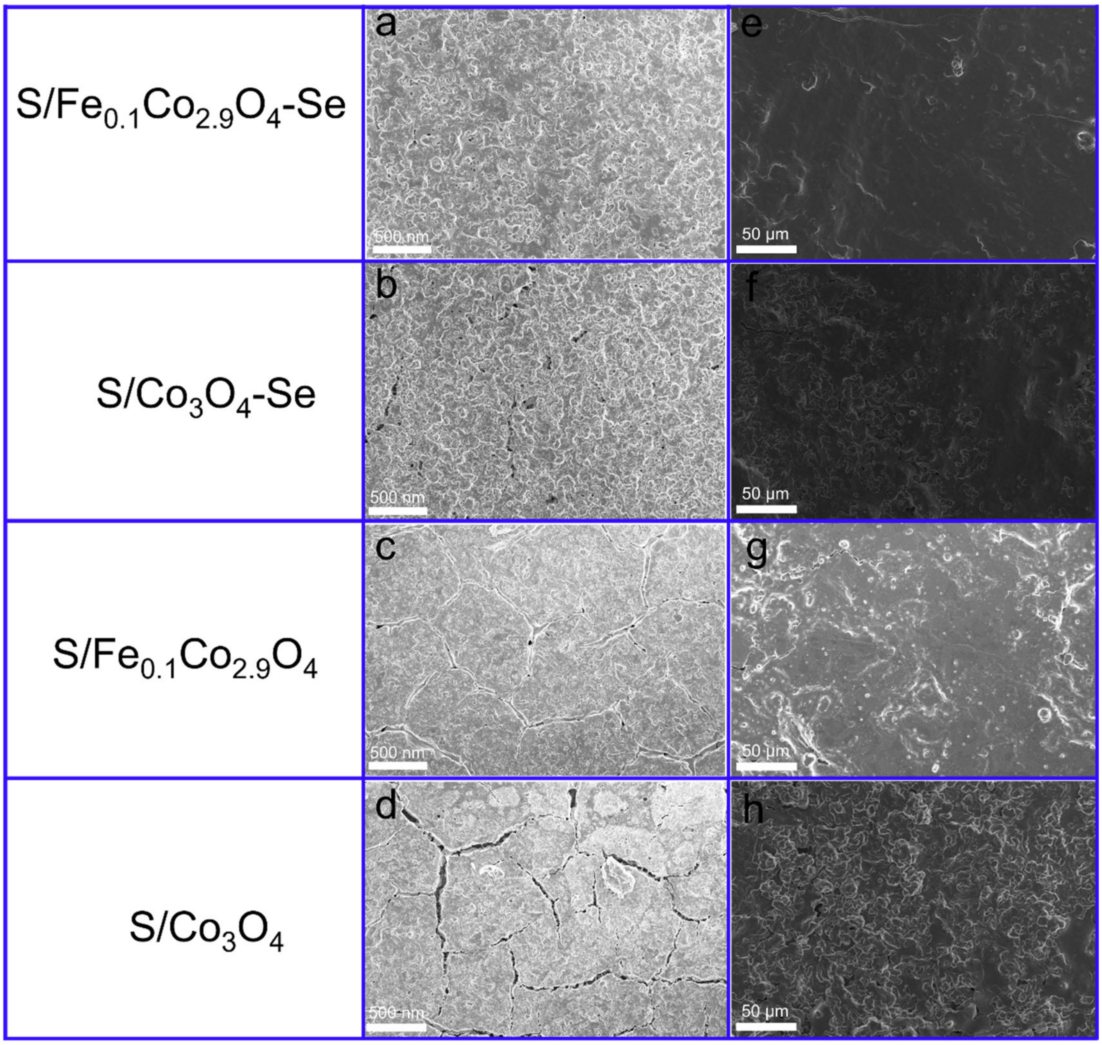

Figure S50. a-d) SEM images of different cathodes after 500 cycles at 0.5 C. e-h) SEM images of different lithium anodes after 500 cycles at 0.5 C.

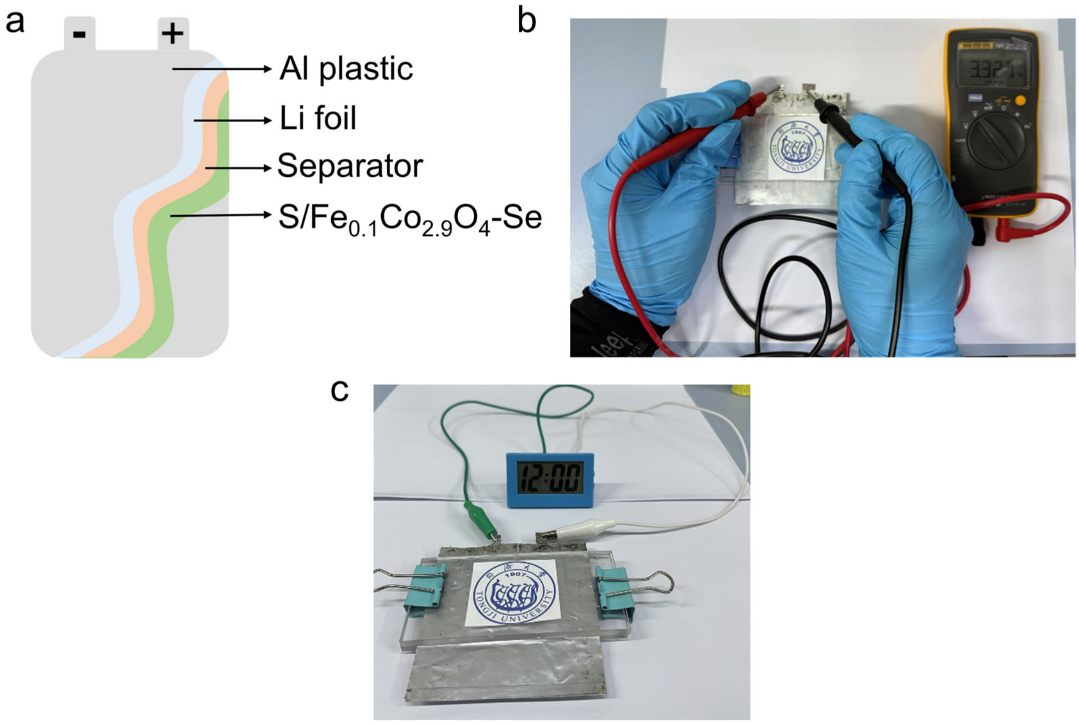

Figure S51. a) A schematic configuration of the structure of the pouch cell. b) Measurement of the open-circuit voltage of the Li-S pouch cell assembled with S/$Fe_{0.1}Co_{2.9}O_4$-Se cathode. c) Optical image of a pouch cell powering the electronic watch.

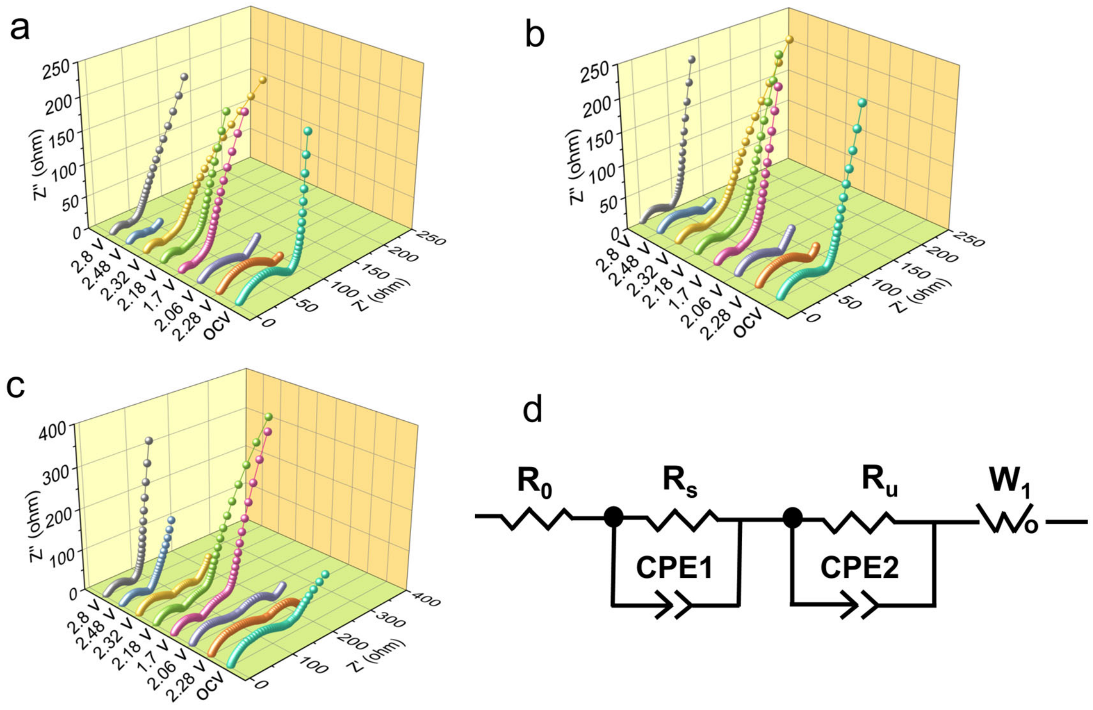

Figure S52. In-situ EIS plots of a) S/Co_3O_4-Se, b) $S/Fe_{0.1}Co_{2.9}O_4$ and c) S/Co_3O_4 during discharge and charge process, respectively. d) Circuit diagram of EIS process simulation.

Table S1. The ICP analysis results of $Fe_{0.1}Co_{2.9}O_4$-Se, Co_3O_4-Se, $Fe_{0.1}Co_{2.9}O_4$ and Co_3O_4.

Material	Composition		
	Co (μg/L)	Fe (μg/L)	Se (μg/L)
$Fe_{0.1}Co_{2.9}O_4$-Se	33.82	1.166	1.91
Co_3O_4-Se	29.54		1.7
$Fe_{0.1}Co_{2.9}O_4$	30.17	1.05	
Co_3O_4	31.72		

Table S2. The atomic percentages of all elements in the XPS full spectra of $Fe_{0.1}Co_{2.9}O_4$-Se, Co_3O_4-Se, $Fe_{0.1}Co_{2.9}O_4$ and Co_3O_4 samples.

Samples	Atomic percentage (at.%)				
	C	O	Fe	Co	Se
$Fe_{0.1}Co_{2.9}O_4$-Se	27.66	43.8	1.23	25.11	2.2
Co_3O_4-Se	32.61	45.37		19.57	2.45
$Fe_{0.1}Co_{2.9}O_4$	28.89	44.45	1.41	25.25	
Co_3O_4	28.43	45.9		25.67	

Table S3. The atomic percentage of Co 2p XPS with different valences in the samples of $Fe_{0.1}Co_{2.9}O_4$-Se, Co_3O_4-Se, $Fe_{0.1}Co_{2.9}O_4$ and Co_3O_4.

Samples	Co^{3+}(%)	Co^{2+}(%)	Co^{2+}/Co^{3+}(%)
$Fe_{0.1}Co_{2.9}O_4$-Se	44.36	55.64	1.25
Co_3O_4-Se	43.72	56.28	1.29
$Fe_{0.1}Co_{2.9}O_4$	46.74	53.26	1.14
Co_3O_4	49.53	50.47	1.02

Table S4. The atomic percentages of O 1s XPS in the samples of $Fe_{0.1}Co_{2.9}O_4$-Se, Co_3O_4-Se, $Fe_{0.1}Co_{2.9}O_4$ and Co_3O_4.

Samples	$O_{(Latt)}$ (%)	$O_{(ads)}$ (%)	$O_{(w)}$ (%)	$O_{(ads)}$/ $O_{(Latt)}$ (%)
$Fe_{0.1}Co_{2.9}O_4$-Se	59.95	34.56	5.5	0.58
Co_3O_4-Se	70.12	21.39	8.48	0.3
$Fe_{0.1}Co_{2.9}O_4$	63.84	27.57	8.6	0.43
Co_3O_4	63.04	20.81	16.15	0.33

Table S5. Structural parameters extracted from the Se *K*-edge EXAFS fitting.

sample	shell	N	R(Å)	σ^2(10^{-3} Å^2)	ΔE_0 (eV)	R factor
$Fe_{0.1}Co_{2.9}O_4$-Se	Se-O	1.4±0.2	1.63±0.0.01	0.0034	4.5±1.2	0.0125
	Se-Se	1±0.2	2.37±0.01	0.002	6.5±0.8	

N: coordination numbers; *R*: bond distance; σ^2: Debye-Waller factors; ΔE_0: the inner potential correction. *R* factor: goodness of fit. S_0^2 was fixed to 1 for $Fe_{0.1}Co_{2.9}O_4$-Se sample fitting.

Table S6. Comparisons of electrochemical performance for the S/$Fe_{0.1}Co_{2.9}O_4$-Se cathode with low-sulfur loading and those reported results in literature for LSBs with Co_3O_4 doped electrodes.

Materials	Sulfur Loading (mg cm^{-2})	Initial capacity at low current (mAh g^{-1})	Initial capacity at high current (mAh g^{-1})	Cycles (rate)/ Decay rate (%)	Ref.
S/$Fe_{0.1}Co_{2.9}O_4$-Se	1.5	1432 (0.1 C)	637 (2 C)	300 (0.2 C) 0.089 %	**This work**
	1.5	1291 (0.2 C)	907 (1 C)	300 (2 C) 0.041 %	
S@Fe(0.1)Co_3O_4	1	1286 (0.2 C)	902.4 (1 C)	150 (0.2 C) 0.17 %	4
S/Fe-Co_3O_4 HHNPs	1	1265 (0.2 C)		100 (0.2 C) 0.391 %	5
Fe-Co_3O_{4-x}(0.25)	1	1346 (0.1 C)	768 (1 C)	80 (0.1 C) 0.25 %	6
S-N-Co_3O_4@N-C	2.13	1223 (0.2 C)		300 (0.2 C) 0.13 %	7
S/W_x-Co_3O_4	1.2	1217 (0.2 C)	857 (1 C)	500 (1 C) 0.052 %	8
S@Co_3O_4-NEGF	3	677 (0.2 C)		100 (0.2 C) 0.1 %	9
Co_3O_4-S	1.3	912 (0.2 C)	603 (2 C)	200 (0.5 C) 0.11 %	10
NCNT@Co-Co_3O_4@S	1	1216 (0.2 C)	801 (1 C)	500 (2 C) 0.023 %	11
S@Co_3O_4/NCNT/LCNT	1.3	1300 (0.2 C)	850 (1 C)	500 (0.5 C) 0.173	12
S@Fe-Co_3O_4	1	1222 (0.2 C)	890 (1 C)	100 (0.2C) 0.187 %	13
P-Co_3O_4/NCNT	1.5	1228 (0.2 C)	900 (1 C)	100 (0.2 C) 0.43 %	14
S-TiO_2/Co_3O_4-CNTs	1	1157 (0.2 C)	803 (1 C)	100 (0.2 C) 0.179 %	15
N-Co_3O_4/S	1.7	1198 (0.2 C)		100 (0.2 C) 0.244 %	16
In_2O_3@NC-Co_3O_4	0.8	1030 (0.2 C)	928 (1 C)	100 (0.2 C) 0.018 %	17
NC@CoP-Co_3O_4	1	1042 (0.2 C)	703 (1 C) 643 (2 C)	200 (1 C) 0.149 %	18

Supporting References

[1] Giannozzi, P.; Baroni, S.; Bonini, N.; Calandra, M.; Car, R.; Cavazzoni, C.; Ceresoli, D.; Chiarotti, G. L.; Cococcioni, M.; Dabo, I.; et al. *J. Phys.: Condens. Matter* **2009**, *21*, 395502.

[2] Perdew, J. P.; Burke, K.; Ernzerhof, M. *Phys. Rev. Lett.* **1996**, *77*, 3865-3868.

[3] Grimme, S. *J. Comput. Chem.* **2006**, *27*, 1787-1799.

[4] W. Wang, Y. Zhao, Y. Zhang, J. Wang, G. Cui, M. Li, Z. Bakenov, X. Wang, *ACS Appl. Mater. Interfaces* **2020**, 12, 12763.

[5] J. Liu, G. Li, D. Luo, J. Li, X. Zhang, Q. Li, H. Li, Y. Zhang, Z. Chen, *Adv. Funct. Mater.* **2023**, 34, 2303357.

[6] J. Li, Z. Wang, T. Zeng, M. Wang, H. Cui, Y. Liu, H. Wei, *Electrochim. Acta* **2023**, 467, 143116.

[7] J. Xu, W. Zhang, Y. Chen, H. Fan, D. Su, G. Wang, *J. Mater. Chem. A* **2018**, 6, 2797.

[8] S. Wang, R. Hu, D. Yuan, L. Zhang, C. Wu, T. Ma, W. Yan, R. Wang, L. Liu, X. Jiang, H. K. Liu, S. X. Dou, Y. Dou, J. Xu, *Carbon Energy* **2023**, 5, e329.

[9] N. Manna, R. Soni, R. S. Young, R. Jervis, T. S. Miller, *Chem. Eur.* **2025**, 12, e202400488

[10] Y. Chen, X. Ji, *J. Alloys Compd.* **2019**, 777, 688.

[11] J. Li, Y. Chen, S. Zhang, W. Xie, S.-M. Xu, G. Wang, M. Shao, *ACS Appl. Mater. Interfaces* **2020**, 12, 49519.

[12] D. Bosubabu, J. Sivaraj, P. Gurunathan, K. Ramesha, *Energy & Fuels* **2020**, 34, 16810.

[13] N. Yan, X. Zhuang, H. Zhang, H. Lu, *Nanomaterials* **2023**, 13, 1612.

[14] Y. Huang, D. Lv, G. Zhang, Y. Cai, Q. Li, H. Wang, Z. Ma, *Energy & Fuels* **2022**, 36, 3339.

[15] W. Qiu, J. Li, Y. Zhang, G. Kalimuldina, Z. Bakenov, *Nanotechnology* **2020**, 32, 075403.

[16] W. Ma, X. Zhang, Y. Meng, J. Zhao, *Ionics* **2021**, 28, 629.

[17] T. Wang, F. Wang, Z. Shi, S. Cui, Z. Zhang, W. Liu, Y. Jin, *ACS Appl. Mater. Interfaces* **2024**, 16, 31158.

[18] X. Zhang, Z. Yu, C. Wang, Y. Gong, B. Ai, L. Zhang, J. Wang, *J. Mater. Sci.* **2021**, 56, 10030.
